# Identification of New Markers of Alcohol-Derived DNA Damage in Humans

**DOI:** 10.3390/biom11030366

**Published:** 2021-02-27

**Authors:** Valeria Guidolin, Erik S. Carlson, Andrea Carrà, Peter W. Villalta, Laura A. Maertens, Stephen S. Hecht, Silvia Balbo

**Affiliations:** 1 Division of Environmental Health Sciences, University of Minnesota, Minneapolis, MN 55455, USA; guido019@umn.edu; 2 Masonic Cancer Center, University of Minnesota, 2231 6th Street SE, Minneapolis, MN 55455, USA; erik_carlson@fas.harvard.edu (E.S.C.); andrea.carra.101184@gmail.com (A.C.); villa001@umn.edu (P.W.V.); maert006@umn.edu (L.A.M.); hecht002@umn.edu (S.S.H.)

**Keywords:** acetaldehyde, DNA adducts, adductomics, mass spectrometry, alcohol

## Abstract

Alcohol consumption is a risk factor for the development of several cancers, including those of the head and neck and the esophagus. The underlying mechanisms of alcohol-induced carcinogenesis remain unclear; however, at these sites, alcohol-derived acetaldehyde seems to play a major role. By reacting with DNA, acetaldehyde generates covalent modifications (adducts) that can lead to mutations. Previous studies have shown a dose dependence between levels of a major acetaldehyde-derived DNA adduct and alcohol exposure in oral-cell DNA. The goal of this study was to optimize a mass spectrometry (MS)-based DNA adductomic approach to screen for all acetaldehyde-derived DNA adducts to more comprehensively characterize the genotoxic effects of acetaldehyde in humans. A high-resolution/-accurate-mass data-dependent constant-neutral-loss-MS^3^ methodology was developed to profile acetaldehyde-DNA adducts in purified DNA. This resulted in the identification of 22 DNA adducts. In addition to the expected *N*^2^-ethyldeoxyguanosine (after NaBH_3_CN reduction), two previously unreported adducts showed prominent signals in the mass spectra. MS^n^ fragmentation spectra and accurate mass were used to hypothesize the structure of the two new adducts, which were then identified as *N*^6^-ethyldeoxyadenosine and *N*^4^-ethyldeoxycytidine by comparison with synthesized standards. These adducts were quantified in DNA isolated from oral cells collected from volunteers exposed to alcohol, revealing a significant increase after the exposure. In addition, 17 of the adducts identified in vitro were detected in these samples confirming our ability to more comprehensively characterize the DNA damage deriving from alcohol exposures.

## 1. Introduction

Globally, annual alcohol consumption has been estimated to be 6.4 L per capita in 2016, and it is projected to increase to 7 L by 2025 [1]. Alcohol is classified by the International Agency for Research on Cancer (IARC) as a Group 1 human carcinogen and is estimated to be responsible for 12.6% of overall cancers [2,3], a percentage expected to increase following the growth in consumption. Alcohol is mainly metabolized in the body by alcohol dehydrogenases (ADHs), which oxidize ethanol to acetaldehyde, followed by detoxification to acetate by aldehyde dehydrogenases (ALDHs) [4]. The variant allele ALDH2*2 encodes for an inactive subunit of the enzyme ALDH2 [4]. Individuals who are heterozygous carriers of this variant, ALDH2*1/*2, have about 10% residual ALDH2 activity and experience side effects like flushing and nausea when ingesting alcohol [4]. These individuals inefficiently detoxify acetaldehyde and are at higher risk for developing alcohol-related esophageal and head and neck cancers [4,5]. These observations contributed to the reclassification of acetaldehyde associated with alcohol consumption as a Group 1 human carcinogen by IARC [3]. Acetaldehyde reacts with DNA bases to produce adducts, which are critical in the carcinogenic process because they can cause miscoding resulting in mutated genes and loss of normal cellular growth-control mechanisms [4]. Although ethanol is mainly metabolized in the liver, the concentration of acetaldehyde in saliva after ingesting ethanol is much higher than in the blood, due to the local metabolism in the oral mucosa and the microflora. Therefore, acetaldehyde genotoxicity may play a specific key role in ethanol-induced carcinogenesis in the oral cavity [4].

The major adduct formed upon reaction of acetaldehyde with DNA is *N*^2^-ethylidenedeoxyguanosine (*N*^2^-ethylidene-dG), which can be analyzed as its more stable reduced version *N*^2^-ethyldeoxyguanosine (*N*^2^-ethyl-dG) after DNA treatment with NaBH_3_CN [4]. Levels of this adduct showed a positive dose-response relationship in oral-cell DNA collected from volunteers who consumed increasing amounts of alcohol [6]. However, several other adducts as well as DNA–DNA crosslinks have been identified in the reactions of acetaldehyde with DNA and, together with modifications at other nucleobases, may also play a role in acetaldehyde’s chemically induced carcinogenesis and epigenetic signaling [7,8,9]. To better understand the role of these other lesions, this study optimized and implemented our ultrasensitive data-dependent acquisition constant-neutral-loss triggered-MS^3^ (DDA-CNL/MS^3^) DNA adductomic method to screen for all acetaldehyde-derived DNA adducts and comprehensively characterize acetaldehyde-derived DNA damage. This approach allowed us to profile acetaldehyde-derived DNA modifications and successfully identify and screen for new markers of alcohol exposure and genotoxicity in humans.

## 2. Materials and Methods

*Caution:* acetaldehyde may cause cancer. It should be handled in a well-ventilated hood with extreme care and with personal protective equipment.

### 2.1. Materials and Chemicals

Acetaldehyde and [ethyl-D_5_]EtNH_3_Cl were purchased from Millipore Sigma (St. Louis, MO, USA). In addition, 6-Chloropurine-2′-deoxyriboside was obtained from Carbosynth (Compton, UK). Water (LC-MS grade), methanol (MeOH, LC-MS grade), acetonitrile (ACN, LC-MS grade), 2-propanol (IPA, LC-MS grade), and formic acid (FA, 98% *v*/*v*) were purchased from Fisher Scientific (Hanover Park, IL, USA). Distilled water was purified by a Milli-Q system (Milford, MA, USA). Deoxyribonuclease I recombinant expressed by *Pichia pastoris* (R-DNase, 10,000 U/mg, phosphodiesterase-1 extracted from *Crotalus adamanteus* (PDE-1, 0.4 U/mg, recombinant alkaline phosphatase expressed by *Pichia pastoris* (R-ALP, 7000 U/mg, calf thymus DNA (CT-DNA, 5 mg), NaBH_3_CN, acetaldehyde, Tris base, double-filtration membrane Amicon Ultra (30 kDa cutoff, 0.5 mL), and single-filtration membrane Microcone (10 kDa cutoff, 0.5 mL) were purchased from Millipore Sigma (St. Louis, MO, USA). Silanized vials (0.3 mL, 1.2 mL, 4 mL, 20 mL) were purchased from ChromTech (Apple Valley, MN, USA). Cell lysis solution, protein precipitation solution, RNase A, and proteinase K were obtained from Qiagen (Hilden, Germany).

### 2.2. General Synthetic Procedures

NMR spectra were recorded on a Bruker 500 MHz spectrometer. Chemical shifts are reported as parts per million (ppm). Residual solvent peaks were used as an internal reference for ^1^H-NMR (7.26 ppm CDCl_3_; 2.50 ppm D_6_-DMSO) and ^13^C-NMR (77.2 ppm CDCl_3_; 39.5 ppm D_6_-DMSO). Peak splitting used the following abbreviations: s = singlet, d = doublet, t = triplet, q = quartet, dd = doublet of doublets, dt = doublet of triplets, ddd = doublet of doublet of doublets, bs = broad singlet, and m = multiplet. All compound structures were evaluated and confirmed with ^1^H, ^13^C, COSY, HSQC, and HMBC experiments. High-resolution mass spectrometry (HRMS) for selected compounds was performed on an Orbitrap Fusion Tribrid mass spectrometer (Thermo Scientific, Waltham, MA, USA) and reported as *m*/*z*. Thin-layer chromatography (TLC) utilized Polygram precoated silica gel TLC plates (40 × 80 mm, 0.2 mm thick) with 254 nm fluorescent indicator. TLC plates were visualized by UV lamp irradiation. Flash chromatography was performed on SiliCycle 60 (70–150) mesh silica gel. Reactions were performed with oven-dried glassware and under an atmosphere of N_2_, unless specified otherwise.

### 2.3. Synthesis of 3′, 5′-bis-O-acetyl-2′-deoxyuridine

To a 25 mL, round-bottom flask equipped with a magnetic stir bar, were added 2′-deoxyuridine (154.9 mg, 0.679 mmol), 4-dimethylaminopyridine (5.6 mg, 0.0458 mmol), triethylamine (275.9 mg, 2.73 mmol, 380 µL), and ACN (3.5 mL). The resulting suspension was treated with acetic anhydride (280.8 mg, 2.75 mmol, 260 µL) and stirred at room temperature for 30 min. The reaction was quenched with MeOH (1 mL) and evaporated in vacuo. The resulting oil was reconstituted in CH_2_Cl_2_ and washed once with brine. The organic layer was dried over MgSO_4_, filtered, and evaporated to a crude foam. Purification by flash column chromatography (1:3:1 hexanes/EtOAc/CH_2_Cl_2_) provided pure product as a white foam (183.3 mg, 86.5%).

### 2.4. Synthesis of 4-chloro-1-N-(3′, 5′-bis-O-acetyl-2′-deoxyribosyl)-2-pyrimidinone

To an oven-dried, two-neck, 25 mL flask equipped with a magnetic stir bar were added 3′,5′-bis-*O*-acetyl-2′-deoxyuridine (293.3 mg, 0.94 mmol) and anhydrous CHCl_3_ (10 mL). The flask was purged with argon thrice and then SOCl_2_ (85.3 mg, 7.17 mmol, 520 µL) and DMF (50 µL) were added. The resulting yellow solution was brought to reflux (~75 °C) for 2 h. After this time, the reaction mixture was cooled to room temperature and quenched with NaHCO_3_ (~5 mL). Once bubbling ceased, the organic layer was collected, and the remaining aqueous layer was extracted once with CH_2_Cl_2_ (10 mL). The pooled organics were dried over MgSO_4_, filtered, and evaporated in vacuo to a yellow oil. Purification by column chromatography (1% → 2% MeOH in CHCl_3_) delivered pure product as an off-white solid (186.5 mg, 60%).

### 2.5. Synthesis of N^4^-ethyldeoxycytidine

4-Chloro-1-*N*-(3′, 5′-bis-*O*-acetyl-2′-deoxyribosyl)-2-pyrimidinone (7.4 mg, 0.0224 mmol), K_2_CO_3_ (30.8 mg, 0.223 mmol), and ACN (1 mL) were combined in a two-dram vial equipped with a magnetic stir bar. The cloudy suspension was treated with EtNH_3_Cl (10.1 mg, 0.124 mmol) and heated to 50 °C for 2 h. After cooling to room temperature, the solvent was removed in vacuo. The residue was then reconstituted in MeOH (1 mL) and stirred at 50 °C for an additional 2 h. The mixture was similarly cooled to room temperature and concentrated to dryness in vacuo. The resulting solid was reconstituted in H_2_O (3 mL) and purified by HPLC (Agilent 1100 Analytical Flow, Agilent Technologies, Palo Alto, CA, USA). Separation was performed using a Luna C18 column (250 × 4.6 mm, 100 A, 40 °C) with a multistep gradient at a flow rate of 1 mL/min using H_2_O and MeOH as solvents A and B, respectively. Beginning at 2% B, the eluent was brought to 27% B over 15 min. This was followed by a wash at 95% B for 2 min and re-equilibration. Detection was accomplished using UV-Vis (254 nm). The product eluted at 16.2 min and was collected in a glass vial. After evaporation in vacuo, pure product was isolated as a white solid (3.72 mg, 65.1%).

### 2.6. Synthesis of [D_5_]N^4^-ethyldeoxycytidine

This compound was produced analogously to *N*^4^-ethyldeoxycytidine (*N*^4^-ethyl-dC), except that [ethyl-D_5_]EtNH_3_Cl was used. The product was isolated as a white solid (5.61 mg, 64.2%).

### 2.7. Synthesis of N^6^-ethyldeoxyadenosine

6-Chloropurine-2′-deoxyribose (11.3 mg, 0.0417 mmol), EtNH_3_Cl (5.1 mg, 0.0626 mmol), iPr_2_EtN (13.3 mg, 0.103 mmol, 18 µL) and DMSO (1 mL) were combined in a two-dram vial equipped with a magnetic stir bar. The solution was heated to 50 °C and stirred for 16 h. The solution was then cooled to room temperature and diluted with H_2_O (1 mL). The product was purified by HPLC (Agilent 1100 Analytical Flow, Agilent Technologies, Palo Alto, CA, USA). Separation was performed using a Luna C18 column (250 × 4.6 mm, 5 A, 25 °C) with a multistep gradient at a flow rate of 1.5 mL/min using H_2_O and MeOH as solvents A and B, respectively. The eluent was held at 2% B for 10 min and then brought to 50% B over 25 min. This was followed by a wash at 95% B for 4 min and re-equilibration. Detection was accomplished using UV-Vis at 254 nm. The product eluted at 22.4 min and was collected in a glass vial. After evaporation in vacuo, pure product was isolated as a white solid (3.60 mg, 30.5%).

### 2.8. Synthesis of [D_5_]N^6^-ethyldeoxyadenosine

This compound was produced analogously to *N*^6^-ethyldeoxyadenosine (*N*^6^-ethyl-dA), except that [ethyl-D_5_]EtNH_3_Cl was used. The product was isolated as a white solid (3.29 mg, 27.8%).

### 2.9. DNA Incubation with Acetaldehyde and Stabilization

The reaction of acetaldehyde with exocyclic amino groups of the DNA nucleobases forms unstable Schiff bases, which may be degraded during DNA hydrolysis [10,11]. To prevent degradation, the DNA was treated with the reducing agent NaBH_3_CN following a previously reported procedure [12]. Similarly, DNA hydrolysis was carried out as previously reported [12]. In brief, CT-DNA (1 mg) was incubated with acetaldehyde (5 mmol) in Tris-10 Mm HCl/5 mM MgCl_2_ buffer (pH ~7) at 37 °C for 24 h. Subsequently, 30 mg NaBH_3_CN was added and the resulting solution incubated at room temperature (RT) overnight. NaBH_3_CN and acetaldehyde negatively impact enzyme activity (unpublished data), resulting in low hydrolysis rates. For this reason, three different protocols of NaBH_3_CN and acetaldehyde removal were evaluated and the one resulting in the best hydrolysis yield was selected (Supplementary Information, SI). Treated DNA was precipitated and desalted via addition of cold IPA, washed with 70% IPA and 100% IPA sequentially, dried under a stream of N_2_, and stored at −20 °C until analysis. The DNA recovery (~98%) for each sample was assessed by UV-Vis-spectrophotometry.

To evaluate concentration-dependent formation of DNA adducts due to acetaldehyde exposure, CT-DNA (1 mg) was incubated with increasing amounts of acetaldehyde (0, 1, 2.5, 5, 25, and 50 mmol) and processed as above. Acetaldehyde concentrations were selected to follow experimental procedures previously reported [8,13].

### 2.10. DNA Hydrolysis and Quantification

DNA was solubilized in 1 mL of 10 mM Tris-HCl/5 mM MgCl_2_ buffer. DNA concentrations were assessed by UV-Vis-spectrophotometry. A total of 250 µg of DNA was hydrolyzed as reported [11]. The digestion yields were assessed by quantifying dG by UPLC-UV (Ultimate 3000, Thermo Scientific, Waltham, MA, USA). The recovery of DNA adducts was evaluated by adding a mixture of isotopically labeled internal standards (100 fmol of [^15^N_5_]*N*^2^-ethyl-dG, [^15^N_5_]*N*^6^-methyl-dA, and [D_4_]POB-dT) into the samples [12].

### 2.11. Sample Purification and Enrichment

Hydrolyzed DNA samples were purified by HPLC fraction collection (FC). The system consisted of an HPLC (Ultimate 3000, Thermo Scientific, Waltham, MA, USA), equipped with a C18 column (4.6 × 250 mm, 100 Ǻ, 5 µm Luna-Phenomenex, Torrance, CA, USA). Two different FC methods were developed and optimized. The first method was used during the initial screening experiments: the instrument was operated at 25 °C with a multistep gradient using H_2_O and MeOH as mobile phase A and B, respectively. The eluent was held at 2% B and 0.5 mL/min for 5 min, brought to 1 mL/min in 1 min, then to 15% B in 24 min, to 35% in 5 min, and finally to 100% B in 5 min. This was followed by a wash at 100% B for 5 min and re-equilibration. Detection was accomplished using the UV-Vis detector set at 190 nm and 254 nm. The unmodified nucleobases were collected separately from the other fractions.

The second method was developed once the standards of the characterized adducts were synthesized to obtain the highest recovery of our analytes. The instrument was operated at 25 °C, performing a multistep gradient at a flow rate of 1 mL/min using H_2_O and MeOH as mobile phase A and B, respectively. The eluent was held at 2% B for 2 min, brought to 12% B in 10 min, then to 15% B in 3 min, to 20% B in 3 min, and finally to 100% B in 3 min. This was followed by a wash at 100% B for 5 min and re-equilibration. As reported above, detection was accomplished using the UV-Vis detector probing two different wavelengths at 190 and 254 nm. Unmodified nucleobases were collected separately from the other fractions. All collected fractions were subsequently dried under reduced pressure and stored at −20 °C until LC-MS analysis.

### 2.12. Oral-Cell DNA Collected from Volunteers Exposed to Known Amounts of Alcohol

Samples were collected as part of a study conducted at the University of Minnesota. The study was approved by the University of Minnesota Human Research Protection Programs Institutional Review Board. Volunteers were enrolled after signing a consent form and evaluation of the eligibility criteria. Medical history and alcohol-drinking history, both in the past 12 months and lifetime, were obtained through questionnaires. Oral rinse samples collected before and 2 h after alcohol exposure (resulting in a blood alcohol concentration (BAC) of 0.11%) were used to isolate DNA for the analysis of acetaldehyde-derived DNA adducts. Details of the study are reported in the SI.

### 2.13. DNA Isolation and Purification from Oral Rinse Samples

A total of 18 oral-rinse samples collected from healthy volunteers, nine before and nine after (2 h) consumption of alcohol, were processed. Samples were centrifuged and the supernatant was removed. The pellet was resuspended in 1 mL of cell lysis solution and treated with proteinase K (24 h, RT), followed by treatment with RNase A (2 h at RT). Proteins were precipitated with 0.3 mL of protein precipitation solution. The supernatant was poured into an equal volume of ice-cold IPA (100%) to precipitate the DNA. Samples were then centrifuged. The supernatant was discarded, and the remaining DNA pellet was washed with 75% and 100% cold IPA. The liquid washes were discarded, and the residual IPA evaporated under a mild N_2_ stream. Dried samples were stored at −20 °C. The extraction yield was assessed by quantifying the DNA using a UV-Vis-spectrophotometer (BioPhotometer, Eppendorf, Hamburg, Germany). DNA hydrolysis and sample purification and enrichment were performed as reported above.

### 2.14. LC Conditions for MS Analysis

Adductomic methods were optimized using an Orbitrap Fusion Tribrid mass spectrometer (Thermo Scientific, Waltham, MA, USA) interfaced to a nanoUPLC (UltiMate 3000 RSLCnano, Thermo Scientific, Waltham, MA, USA) with a NanoFlex ion source (Thermo Scientific, Waltham, MA, USA), operating in positive ionization mode with a voltage of 2.5 kV and an ion tube temperature of 300 °C. The UPLC system was equipped with a 5 µL loop and a reverse-phase column home-packed (silica emitter 230 × 0.075 mm, 15 um orifice, New Objective, Woburn, MA, USA) with C18 stationary phase (5 μm, 100 Ǻ, Luna-Phenomenex, Torrance, CA, USA). The mobile phase consisted of formic acid (0.05% *v*/*v* in H_2_O, phase-A) and ACN (100% *v*/*v*, phase-B).

For the untargeted screening, the eluent was held at 2% B for 2 min, brought to 20% B in 24 min, then to 60% B in 10 min, to 98% B in 1 min, and then maintained at 98% for 4 min. This was followed by a wash at 98% B for 4 min and column re-equilibration. For the targeted analysis, the eluent was held at 2% B for 6 min, brought to 35% B in 14 min, then to 98% B in 2 min, and kept at 98% B for 2 min, followed by column re-equilibration.

### 2.15. DDA-CNL/MS^3^ Gas-Phase Fractionation Method

Purified DNA extracted from rat liver was available from previous studies and was used as the matrix for our method development. DNA was enzymatically hydrolyzed and purified as reported above. A standard mixture of six isotopically labelled DNA adducts ([^15^N_5_]*N*^2^-ethyl-dG, [^15^N_5_]*N*^6^-methyl–dA, [D_4_]*O*^6^-POB-dG, [D_4_]*O*^6^-POB-dT, [D_4_]*O*^6^-PHB-dG, [^15^N_5_]8-OH-PdG (structures in SI)) was prepared and spiked in the matrix previously reconstituted in 20 µL of LC-MS H_2_O prior to LC-MS analysis.

The MS analysis was performed with Orbitrap detection (resolution of 60,000) in: (i) gas-phase fractionation mode with the mass range of interest split into four scan segments (*m*/*z* 197–310, *m*/*z* 305–380, *m*/*z* 375–450, and *m*/*z* 445–750) or (ii) in standard mode with a single scan segment (*m*/*z* 197–750). In each partial or full scan, quadrupole filtering was used with a maximum injection time of 200 ms and an automatic gain control (AGC) setting of 5.0 × 10^4^.

For each scan segment, the top five ions were selected for MS^2^ fragmentation with quadrupole isolation of 1.5 *m*/*z*, using collision induced dissociation (CID) with a normalized collision energy of 30%, maximum injection time of 200 ms, and Orbitrap detection at a resolution of 30,000. An exclusion list of 95 ions (SI) with a mass tolerance of 5 ppm was used, as was dynamic exclusion of 30 s and an intensity threshold of 2.0 × 10^3^. MS^3^ fragmentation was triggered upon observation of the accurate-mass neutral loss of 2′-deoxyribose (-dR: 116.0474 ± 0.0006 *m*/*z*, 5 ppm) upon MS^2^ fragmentation. MS^3^ fragmentation was performed with high-energy collisional dissociation (HCD) with a normalized collision energy of 50%, maximum injection time of 250 ms, and Orbitrap detection at a resolution of 15,000. This gas-phase fractionation MS method was used for DNA-adduct profiling.

### 2.16. Targeted Mass Spectrometry-Based Approach

To attain the highest level of sensitivity in order to investigate the presence of the adducts, previously characterized in the in vitro experiment, in human oral-cell DNA, a targeted MS^2^ analysis was performed with the parent ion masses listed in Table 1, and the internal standard ion masses (*m*/*z* 301.1205, *m*/*z* 394.1911, and *m*/*z* 424.2191). Subsequently for the absolute quantitation of ethyl-adducts, a targeted MS^2^ analysis was performed with eight parent ion masses (*m*/*z* 256.1292, *m*/*z* 261.1605, *m*/*z* 280.1404, *m*/*z* 285.1718, *m*/*z* 296.1353, *m*/*z* 301.1205, *m*/*z* 394.1911, and *m*/*z* 424.2191). The following parameters were set for the analysis: RF lens of 60%, quadrupole isolation window of 1.5 *m*/*z*, HCD of 22%, AGC target of 5 × 10^4^, maximum injection time of 50 ms, Orbitrap resolution of 60,000, and EASY-IC enabled. Frozen DNA samples were thawed and reconstituted in 20 µL H_2_O and analyzed.

### 2.17. Method Validation

The ability of the targeted approach to quantify *N*^2^-ethyl-dG, *N*^6^-ethyl-dA, and *N*^4^-ethyl-dC was evaluated, and the method was validated. The limits of detection (LOD) for the quantitation of *N*^2^-ethyl-dG, *N*^6^-ethyl-dA, and *N*^4^-ethyl-dC were established using standard solutions of adducts. The limits of quantitation (LOQ), accuracy, and precision of the method were determined by analyzing CT-DNA spiked with different amounts of *N*^2^-ethyl-dG (0, 2, 6, 10, 40, 100 fmol), *N*^6^-ethyl-dA (0, 0.2, 0.6, 1, 4, 10 fmol), and *N*^4^-ethyl-dC (0, 2, 6, 10, 40, 100 fmol). Each sample was analyzed in triplicate. Background levels of the adducts in CT-DNA were determined by analyzing three nonspiked samples; these amounts were subtracted from the amounts measured in the spiked samples. LODs and LOQs were calculated using the following equations: LOD = (3.3 × sd/S) and LOD = (10 × sd/S), where sd is standard deviation, S is the slope of the calibration curve, and the multipliers (3.3 and 10) are recommended by International Conference on Harmonization standards [14]. Accuracy was determined by comparing added and measured amounts of the adducts at each level. Precision was determined as intraday coefficients of variation (% CV) for the triplicate samples. Recovery was determined by adding [^15^N_5_]*N*^2^-ethyl-dG (10 fmol), [D_5_]*N*^6^-ethyl-dA (1 fmol), and [D_5_]*N*^4^-ethyl-dC (10 fmol) to CT-DNA, processed as described above and compared to CT-DNA samples with analytes added after processing.

### 2.18. Data Processing and Normalization

Putative DNA adducts were identified from LC-MS^3^ data using Xcalibur 3.0 (Thermo Scientific, Sunnyvale, CA, USA), where only ions which triggered an MS^3^ event and were unique or increasing in the exposed samples were considered. For relative quantification of a putative DNA adduct in a specific sample, the area of the full-scan extracted ion chromatogram (EIC) with a mass tolerance of 5 ppm was used, and the intensity was further normalized using the following: 1) amount of dG (µmol) determined in that specific sample and 2) area of the internal standard EIC.

### 2.19. Statistical Analysis

Statistical analyses were performed using SigmaPlot 12.5 (Systat Software, San Jose, CA, USA, https://systatsoftware.com/products/sigmaplot/ (accessed on 15 January 2021)). The Student’s t-test was used to compare DNA-adduct levels between baseline and 2 h exposure. Statistical significance was set at *p* ≤ 0.05.

## 3. Results

A top-down DNA adductomic approach was used to comprehensively characterize adducts derived from the interaction of acetaldehyde with DNA. Our DDA-CNL/MS^3^ adductomic method can simultaneously screen for multiple DNA adducts by taking advantage of the common structural feature of deoxyribonucleosides: a deoxyribose moiety bound to the nucleobase through a glycosidic bond [15]. This results in a common typical MS fragmentation, which involves the neutral loss of the sugar moiety (116.0474 Da). This common feature is used to program the instrument to trigger further additional MS^3^ fragmentation of the ions showing the corresponding diagnostic neutral loss and to gain additional information for structural identification. In this work, this powerful comprehensive screening technique was optimized to achieve maximum sensitivity before analysis of the samples.

### 3.1. Gas-Phase Fractionation for DNA-Adduct Screening

Gas-phase fractionation (GPF) in LC-MS analysis is defined as the division of the mass range of interest into multiple segments. The ability of GPF to improve the measurement of trace levels of DNA adducts was tested in a sample matrix by analyzing rat-liver DNA spiked with standards. A mixture of six isotopically labelled DNA adducts (structures in SI) were analyzed using a DDA-CNL/MS^3^ method with and without GPF in the full-scan data acquisition. Full-scan data (*m*/*z* 250–750) collected from analysis of a similar sample were first used to evaluate the *m*/*z* mass distribution used to guide the determination of the GPF *m*/*z* precursor windows for testing (Figures S1 and S2). The full-scan range was divided into four *m*/*z* ranges with an overlap of 5 Da (*m*/*z* 197–310, *m*/*z* 305–380, *m*/*z* 375–450, and *m*/*z* 445–750). Comparison of the method with and without GPF was done by comparing the number of ions undergoing MS^2^ and MS^3^ fragmentation, including those for the isotopically labelled standards (Figures S1 and S2).

Across two sample sets, an average of 2083 and 240 ions triggered an MS^2^ and MS^3^ event, respectively, when GPF was used, and 1616 and 210 ions triggered an MS^2^ and MS^3^ event, respectively, when GPF was not used. A total of five and two standards triggered MS^2^ and MS^3^ events when GPF was used. On the other hand, only one standard triggered MS^2^ and MS^3^ events when GPF was not used. Results demonstrated that GPF can be a useful tool for increasing overall detection coverage of our method; therefore, this technique was used to perform the screening in vitro.

### 3.2. Screening of Acetaldehyde-Derived DNA Adducts in Exposed CT-DNA Using LC-HRMS in GPF-DDA-CNL/MS^3^ Scan Mode

To comprehensively profile acetaldehyde-derived DNA adducts, we investigated the in vitro reactivity of acetaldehyde with DNA. DNA samples were subjected to NaBH_3_CN reduction to stabilize any Schiff bases generated, following a previously described procedure [12]. Purified DNA was exposed to acetaldehyde and re-isolated using our optimized procedure involving DNA precipitation with IPA (SI). DNA was then resuspended in Tris buffer, and after enzymatic hydrolysis and sample enrichment, the samples were analyzed by LC-HRMS.

**Figure 1:**
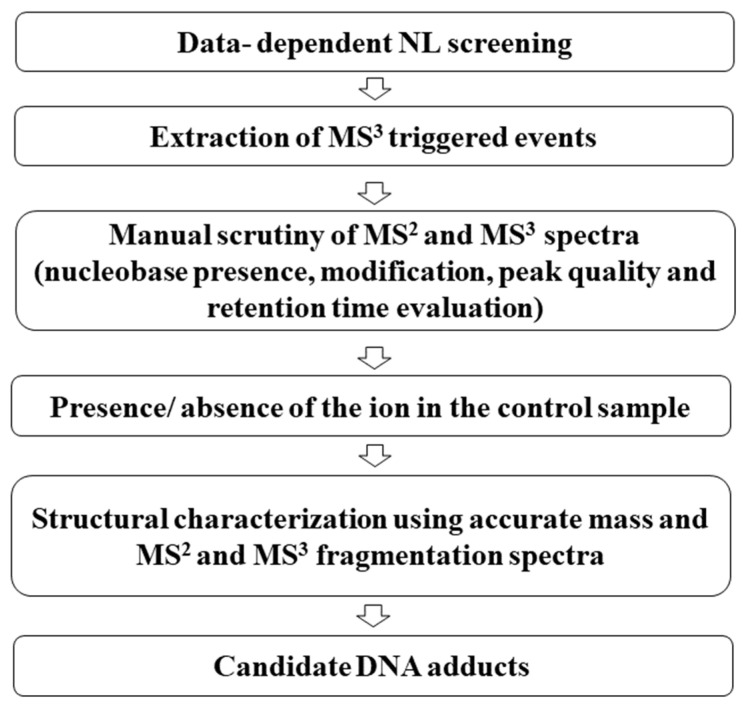
DNA-adductomics data-analysis workflow for DNA-adducts discovery.

A rigorous data-analysis workflow was followed to classify those detected ions which are DNA adducts (Figure 1). The use of our untargeted DNA adductomic approach resulted in the detection of 399 MS^3^-triggering ions in the acetaldehyde-exposed sample. Careful scrutiny of the MS^2^ and MS^3^ spectra for each MS^3^-triggering ion confirmed that the fragments observed were consistent with a DNA adduct and used to exclude any MS^3^-triggering ions resulting from artifacts or false positives. Specifically, for each ion, the MS^3^ spectrum was scrutinized to i) confirm the presence of one of the nucleobases and/or its fragments as product ions and ii) evaluate that the accurate mass corresponding to the modification accounts for a realistic chemical formula. Furthermore, the peak shape of the precursor extracted ion chromatogram (EIC) was evaluated to confirm its Gaussian-like shape and a minimum of four sticks across the peak. Finally, the retention times of the full scan, MS^2^ and MS^3^ spectra were evaluated to confirm that they coincided. Full-scan EICs for all candidate DNA-adduct ions were generated for the exposed and nonexposed samples, and only ions that were uniquely present or increasing (with a signal intensity at least three-fold higher) in the exposed sample were further considered.

**Figure 2:**
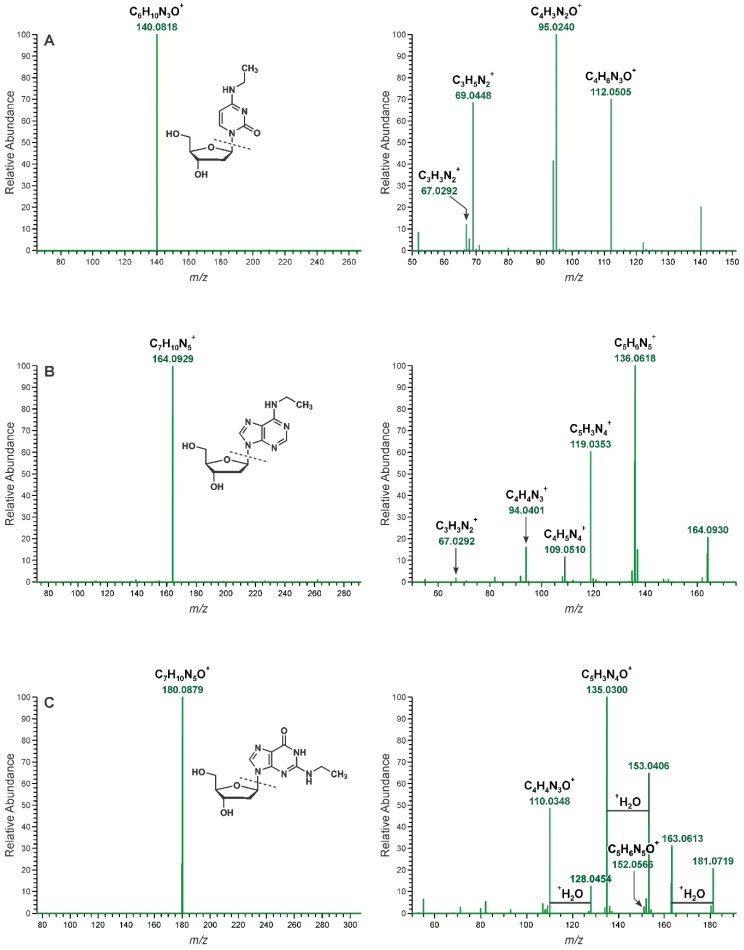
Most intense peaks detected in CT-DNA were assigned to *N*^4^-ethyl-dC (*m*/*z* 256.1292), *N*^6^-ethyl-dA (*m*/*z* 280.1404), and *N*^2^-ethyl-dG (*m*/*z* 296.1353). Panel (**A**): *N*^4^-ethyl-dC MS^2^ and MS^3^ spectra with main fragment structures elucidated. Panel (**B**): *N*^6^-ethyl-dA MS^2^ and MS^3^ spectra with main fragment structures elucidated. Panel (**C**): *N*^2^-ethyl-dG MS^2^ and MS^3^ spectra with main fragment structures elucidated. The three structures were confirmed by comparison with synthetized standards.

This scrutiny of the ions identified by our DNA adductomic approach resulted in the identification of 22 DNA adducts in CT-DNA exposed to acetaldehyde (5 mmol) with NaBH_3_CN reduction. Putative structures were assigned based on MS^2^ and MS^3^ spectra (SI) and ion masses reported in Table 1. The spectra and structural assignments of the three most abundant DNA adducts, based upon the precursor ion signal intensities in the total ion chromatogram (TIC), were confirmed by comparison with synthetized standards and are shown in Figure 2.

Figure 2 shows the MS^2^ and MS^3^ spectra for the three most intense parent ions (*m*/*z* 256.1292, 280.1404, and 296.1353) detected upon exposure of purified DNA to acetaldehyde. The latter is consistent with the known *N*^2^-ethyl-dG (Figure 2, panel C) and the fragmentation spectra agree with those previously reported [16,17,18,19]. The *m*/*z* 256.1292 mass is consistent with that of protonated ethylated deoxycytidine. The MS^2^ spectrum (Figure 2, panel A) is dominated by a single ion (*m*/*z* 140.0818) with a mass consistent with the neutral loss of deoxyribose (116.0474 Da). The MS^3^ spectrum contains an ion which can be assigned to protonated cytosine (*m*/*z* 112.0505) and two ions assignable to cytosine fragment ions (*m*/*z* 95.0240 and 69.0448) [18]. Similarly, the *m*/*z* 280.1404 ion is consistent with protonated ethylated deoxyadenosine [18]. Its MS^2^ spectra is consistent with the neutral loss of the deoxyribose, and the MS^3^ spectra is consistent with the loss of the ethyl group from the M^+^-116 fragment and adenine-specific fragment ions (*m*/*z* 136.0618, 119.0353, and 109.0510.) The scrutiny of these spectra and resulting fragments supported the synthesis of *N*^4^-ethyl-dC and *N*^6^-ethyl-dA, which were used to confirm the structure of these two adducts.

### 3.3. DNA-Adduct Characterization and Synthesis of Stable Isotope-Labeled Standards

**Figure 3:**
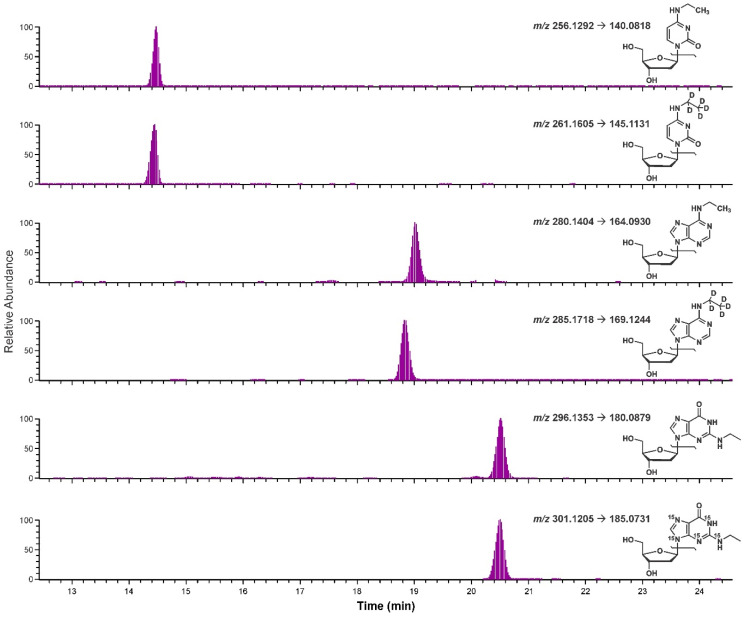
Chromatograms resulting from co-injection of standards with the CT-DNA sample exposed to 5 mmol acetaldehyde. From the top, *N*^4^-ethyl-dC (*m*/*z* 256.1292), [D_5_]*N*^4^-ethyl-dC (*m*/*z* 261.1605), *N*^6^-ethyl-dA (*m*/*z* 280.1404), [D_5_]*N*^6^-ethyl-dA (*m*/*z* 285.1718), *N*^2^-ethyl-dG (*m*/*z* 296.1353), and [^15^N_5_]*N*^2^-ethyl-dG (*m*/*z* 301.1205).

*N*^6^-ethyl-dA, [D_5_]*N*^6^-ethyl-dA, *N*^4^-ethyl-dC, and [D_5_]*N*^4^-ethyl-dC were individually synthesized as reported in Materials and Methods [20,21,22,23]. Solid-phase extraction (SPE) purification was not successful and therefore purification was performed using RP-HPLC-UV (254 nm). The adducts were characterized via HRMS infusion, proton and carbon NMR, COSY, and HMQC analysis (Table S1). The amounts of the synthesized compounds were determined by qNMR [24]. The synthesized standards were used to confirm the identity of the analytes detected in our in vitro experiment. A standard solution of the isotopically labelled compounds ([D_5_]*N*^4^-ethyl-dC, [D_5_]*N*^6^-ethyl-dA, and [^15^N_5_]*N*^2^-ethyl-dG) was co-injected with the previously analyzed CT-DNA sample. The analysis was conducted with a high-resolution targeted approach, monitoring the common loss of 2′-deoxyribose. The retention times of the isotopically labelled standards were consistent with those of the analytes formed by incubation of acetaldehyde and DNA (Figure 3). No chromatographic shoulders or satellite peaks were observed. In addition to co-elution, high-resolution accurate mass and fragmentation of the standards and sample analytes further confirmed the identity of the DNA adducts in the sample (Figure 3).

### 3.4. Concentration-Dependent DNA-Adduct Generation

**Figure 4:**
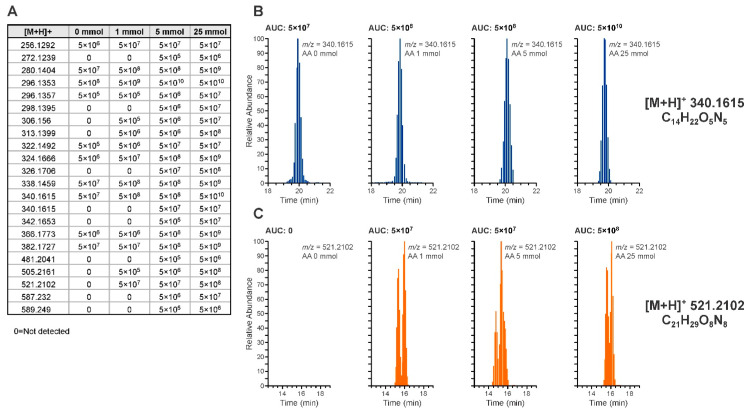
Concentration-dependent formation of the 22 DNA adducts identified in CT-DNA exposed to acetaldehyde. Panel (**A**): ions detected during the adductomic screening and the average signal intensities at increasing acetaldehyde concentrations (0, 1, 5, and 25 mmol). Chromatograms from two representative DNA adducts among those identified are reported illustrating the increase in signal intensity measured as the area under the curve. Panel (**B**): extracted ion chromatogram of *m*/*z* 340.1615 at increasing acetaldehyde concentrations. Panel (**C**): extracted ion chromatogram of *m*/*z* 521.2102 at increasing acetaldehyde concentrations.

The relationship between acetaldehyde concentration and the generation of the 22 DNA candidate adducts reported in Table 1 was tested (Figure 4 and Figure S3). This experiment was performed to investigate the contribution of exogenous acetaldehyde to the formation of the detected adducts.

Adduct levels (intensity of the adduct at a given acetaldehyde concentration) were observed to generally increase with acetaldehyde concentration for all the detected modifications (Figure 4), and some adducts were present in the unexposed DNA. In Figure 4, panels B and C, there were two representative examples of DNA-adduct signals increasing with increasing acetaldehyde exposure. Suggested structures of the two ions are reported in the SI. While the ion *m*/*z* 340.1615 has been hypothesized to be a dG adduct based on its accurate mass and presence of guanine fragmentation in the spectra, for the same reasons, the ion *m*/*z* 521.2102 has been hypothesized to be a dC–dG crosslink.

In the case of the ethyl-adducts, N^2^-ethyl-dG showed the most intense instrumental response reaching a signal plateau at high acetaldehyde concentrations (Figure S3). N^4^-ethyl-dC gave the least intense instrumental response (Figure S3). For all three adducts, a baseline level was observed in calf thymus DNA.

### 3.5. Screening of Acetaldehyde DNA Adducts in Human Oral Cells

Oral-rinse samples were obtained from nine healthy volunteers before and 2 h after exposure to a dose of alcohol, calculated on the weight and sex of each individual [25], resulting in a blood alcohol level of 0.11%, measured 1 h after the dose. DNA was isolated from the oral-rinse samples and processed. Extracted DNA from three participants was pooled to reduce subject-specific variability and increase the amount of DNA to be analyzed, resulting in a total of three DNA samples collected before alcohol exposure and three DNA samples collected after the exposure.

DNA was treated with NaBH_3_CN, and isotope-labeled ethyl-adducts were added as internal standards. Enzyme hydrolysis was performed, and sample clean-up and enrichment via HPLC-fraction collection was completed.

For the NanoLC-HRMS^2^ method, 22 ions detected during the in vitro screening were targeted. The AUC of each putative adduct was normalized by the internal standards AUC and the amount of dG (µmol) measured in the sample.

Out of the 22 DNA adducts detected in vitro and monitored in human oral cells, 17 were detected in the oral DNA samples; six were only detected in the oral cells exposed to alcohol; eight significantly increased in the exposed samples compared to the non-exposed (*p* ≤ 0.05); and three increased, but with a variability resulting in a nonsignificant difference (*p* > 0.05) (Table 2). Finally, five of the adducts were not detected in any of the oral-cell DNA samples (Table 2).

### 3.6. Quantitation of N^4^-ethyl-dC, N^6^-ethyl-dA and N^2^-ethyl-dG in Human Oral Cells

*N*^4^-ethyl-dC and *N*^6^-ethyl-dA were characterized and standards were synthesized. Levels of these adducts were quantified together with those of *N*^2^-ethyl-dG in oral-cell DNA samples from alcohol-exposed volunteers. The method for quantitation of the ethyl-adducts was validated for this study. LODs of 0.13, 0.017, and 0.10 fmol on-column for *N*^4^-ethyl-dC, *N*^6^-ethyl-dA, and *N*^2^-ethyl-dG, respectively, were achieved. The concentration ranges for the calibration curves and validation experiments were chosen to cover the range of the levels of adducts found in human oral-cell samples. The calibration curves showed good linearity within the low concentration range (R^2^ > 0.99). The assay accuracy was calculated as a percentage of the added amount of adducts to 50 μg of CT-DNA and the average accuracies were 122, 137, and 128% (n = 5) for *N*^4^-ethyl-dC, *N*^6^-ethyl-dA, and *N*^2^-ethyl-dG, respectively, and good linearity was observed across the tested concentration ranges (Figure S18). Recoveries averaged 46, 41, and 72%. The estimated LOQ in DNA were 33, 1.8, and 37 fmol/μmol dG, respectively.

**Figure 5:**
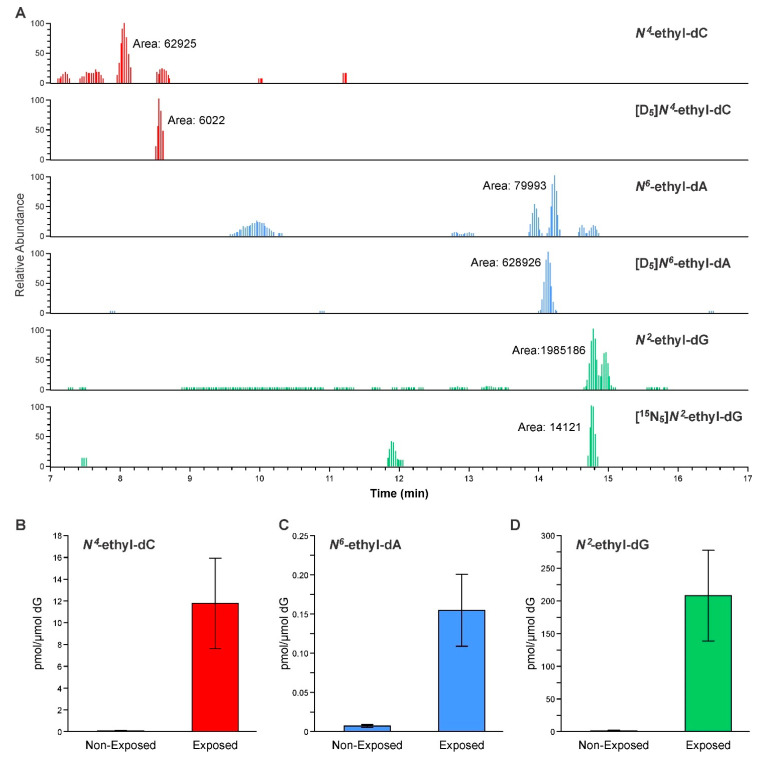
Panel (**A**): NanoLC-HRMS^2^ chromatograms of ethyl-adducts in human oral-cell DNA isolated from alcohol-exposed volunteers. Amounts of *N*^4^-ethyl-dC (panel (**B**)) volunteers, *N*^6^-ethyl-dA (panel (**C**)) volunteers, and *N*^2^-ethyl-dG (panel (**D**)) (pmol/μmol dG) in human oral-cell DNA of nonexposed and alcohol-exposed volunteers.

Representative examples of extracted ion chromatograms for the ethyl-adducts quantitation using NanoLC-HRMS^2^ are shown in Figure 5 (Panel A). *N*^4^-ethyl-dC, *N*^6^-ethyl-dA, and *N*^2^-ethyl-dG were detected in all samples and the peaks of the analytes co-eluted with their corresponding internal standards. The amounts of these ethyl-adducts in oral-cell DNA before and after alcohol exposure are shown in Figure 5 (Panels B–D). The average levels of *N*^4^-ethyl-dC, *N*^6^-ethyl-dA, and *N*^2^-ethyl-dG in samples after exposure were 12, 0.16, and 208 pmol/µmol dG, respectively, whereas in samples before exposure they were 0.08, 0.007, and 1.30 pmol/μmol dG, respectively. The levels of the three adducts all showed a significant increase in the samples collected after alcohol exposure (Figure 5). A negative control using buffer and a positive control using CT-DNA were included and worked up in the same way together with the other samples to ensure data quality. No contamination was observed in the negative controls.

## 4. Discussion

Our work presents a method for comprehensive analysis of acetaldehyde-derived DNA adducts in oral-cell DNA from volunteers exposed to alcohol. The method was used to screen DNA exposed to acetaldehyde using a high-resolution/accurate-mass data-dependent constant-neutral-loss-MS^3^ (DDA-CNL/MS^3^) DNA adductomic approach resulting in the identification of 22 acetaldehyde-derived DNA modifications—some of which have not been described before.

This work was done to expand upon previous studies that characterized reactions of acetaldehyde with DNA and focused on the major covalent binding occurring to dG, while only suggesting the occurrence of reactions with dC and dA [7,8,13]. These previous studies were done using less-selective, and therefore often less-sensitive, technologies in comparison to the ones used in this study, limiting the ability to perform the simultaneous identification and quantitation of multiple modifications.

The LOD of the DDA-CNL/MS^3^ method is limited either by the ion capacity of the Orbitrap to detect ions in the full-scan acquisition or the rate at which MS^2^ spectra can be acquired. Background ion signal in DNA adductomic analyses is many orders of magnitude higher than the DNA-adduct signals which results in low injection times, and thereby limiting the number of analyte ions entering the Orbitrap for detection [26]. To mitigate this issue, the sensitivity of the standard full-scan DDA-CNL/MS^3^ method was enhanced by the implementation of a procedure called “gas-phase fractionation”(GPF), an approach which has been used in the fields of proteomics, metabolomics, and lipidomics to enhance the detection of low levels of analytes of interest [27,28,29]. The use of GPF breaks the full-scan range of interest into multiple small ranges resulting in longer injection times by limiting the mass range of ions sampled in a given full-scan detection event. In this study, a comparison of the performance of the standard method with that of a GPF version was done where the full-scan range was divided into four segments, with overlaps of 5 Da. The GPF version outperformed the standard method as summarized in Figures S1 and S2, with an average of 2083 (MS^2^) and 240 (MS^3^) triggered ions for the GPF method, and 1616 (MS^2^) and 210 (MS^3^) triggered ions for the standard method. Five and two spiked-in DNA adducts triggered MS^2^ and MS^3^ events, respectively, with the GPF method, compared to one spiked-in DNA adduct for both MS^2^ and MS^3^ events when the standard method was used. These results demonstrate that GPF can be a useful tool for increasing overall detection coverage of this method; therefore, this technique was used to perform in vitro screening.

The optimized method was used to comprehensively profile acetaldehyde-DNA adducts in CT-DNA exposed to acetaldehyde and treated with NaBH_3_CN to reduce and stabilize any Schiff bases which formed. Following a rigorous data analysis (Figure 1), 22 DNA adducts were detected and the high-quality spectra and the accurate masses obtained allowed for the assignments of chemical formulas and putative structures, including many dA, dC, dT, and dG adducts (Table 1 and SI). Several of the assigned DNA adducts were previously described, including *N*^2^-ethyl-dG (*m*/*z* 296.1357) and 1,*N*^2^-propano-dG (*m*/*z* 338.1459) which were detected in vivo [6,30,31,32]. Additionally, Wang and coworkers first demonstrated the generation of *N*^2^-dimethyldioxane-dG (*m*/*z* 382.1727) and of the crosslink *m*/*z* 587.2324 [8]. The crosslink *m*/*z* 589.2420 has also been reported as reduction product of *m*/*z* 587.2324 [9,33,34,35]. Our DNA adductomic screening analysis identified new DNA adducts including crosslinks involving dC and monoadducts involving dA, dT, and dC.

Acetaldehyde is ubiquitous and is a product of physiological processes; therefore, acetaldehyde-derived DNA adducts can be detected in samples not exposed to exogenous sources of this genotoxic compound. To characterize the role that exogenous acetaldehyde has on the formation of the 22 observed DNA adducts, the DNA-adduct ion signals were measured in CT-DNA exposed to increasing amounts of acetaldehyde (0, 1, 2.5, 5, 25, and 50 mmol). All adducts showed higher ion intensities as the dose of acetaldehyde augmented.

The 22 adducts were included in a more sensitive, targeted method for the investigation of acetaldehyde-derived DNA damage in human samples. Among the DNA adducts detected, those resulting from the reduction of Schiff bases formed upon reaction of acetaldehyde with the *N*^2^ position of dG, *N*^6^ position of dA, and *N*^4^ of dC corresponded to the most intense signals. The *N*^2^-ethyl-dG-attributed ion signal was the highest followed by those of the adducts of dA and dC, in accordance with the observations by Vaca et al., which reported a reactivity order of dG > dA > dC [7]. The identities of these adducts were confirmed by comparison with synthetic standards. The *N*^2^-ethyl-dG synthetic standard was readily available [6], while *N*^6^-ethyl-dA and *N*^4^-ethyl-dC were synthesized. Isotopically labelled versions of the compounds were synthetized as well for quantitation using an isotope dilution method. Accuracy, precision, and limit of detection were determined for this newly developed quantitative method for *N*^2^-ethyl-dG, *N*^6^-ethyl-dA, and *N*^4^-ethyl-dC, which was combined with the targeted detection of the other 19 adducts identified in the initial screening.

The method was tested to investigate alcohol-derived DNA adducts in oral-cell DNA isolated from oral rinses collected from healthy volunteers before and after consumption of an alcohol dose resulting in a 0.11% BAC. The increase in acetaldehyde concentration in saliva of the study participants after consumption of the alcohol dose was measured by LC-MS. Results from this analysis have been reported in a previous manuscript focusing on the method used for this analysis [36]. In this study, levels of acetaldehyde in saliva were found to increase four- to seven-fold compared to baseline, in samples collected 1 h after the dose. This measurement confirmed that the dose administered indeed resulted in an increase in the levels of acetaldehyde in the saliva of our study participants.

The results of our work further confirmed the role of alcohol-derived acetaldehyde in the induction of DNA damage in the oral cavity, demonstrated by the increase in the levels of the three major acetaldehyde-derived DNA adducts *N*^4^-ethyl-dC, *N*^6^-ethyl-dA, and *N*^2^-ethyl-dG and by the increase in the signal intensity and presence of the other identified adducts in the samples collected after alcohol exposure. These results confirm and expand upon our earlier studies that found a significant increase in the levels of *N*^2^-ethyl-dG at exposures from alcohol doses resulting in a BAC of 0.03% [6]. Additionally, the levels of *N*^2^-ethyl-dG we measured in samples collected before the alcohol dose (1.30 pmol/μmol dG) were consistent with previously reported levels from a similar study measuring the same adduct in samples collected from volunteers before alcohol exposure (1.85 pmol/µmol dG) [5].

This is to our knowledge the first application of a DNA adductomic approach for the comprehensive characterization of acetaldehyde-derived DNA adducts and the first application of a targeted adductomic approach for the investigation of alcohol-related DNA damage in the oral cavity. Previously published studies focused on the detection of alcohol-derived DNA damage using nonspecific methodologies like ^32^P-postlabelling or on the quantitation via LC-MS of *N*^2^-ethyl-dG and *N*^2^-propano-dG in peripheral white-blood-cell DNA of alcoholics [37,38,39]. Indeed, our previous work on oral-cell DNA from volunteers exposed to increasing amounts of alcohol focused exclusively on the quantitation of *N*^2^-ethyl-dG [6].

Our method allowed for the detection of two new acetaldehyde-derived DNA adducts in oral-cell DNA. Furthermore, three putative crosslinks were detected in oral cavity cells. Crosslinks are known to be potentially highly mutagenic [9,34]. Recently, a study evaluating acetaldehyde crosslink repair assessed the presence of error-prone mechanisms of repair of these adducts, involving the Fanconi Anemia pathways and fork-convergence mechanisms [34]. There is a need for further investigations on the relationship between acetaldehyde-DNA modifications and mutations in humans, especially in susceptible populations, and these studies will benefit dramatically from more comprehensive methods like the one we have developed.

In our work, DNA was treated with NaBH_3_CN to stabilize the Schiff bases. The use of a reducing agent effectively stabilized the imines formed on dG, dA, and dC; however, the treatment may prevent or reduce the chances of detecting other DNA adducts, limiting the ability for this method to truly identify all DNA adducts generated by the reaction with acetaldehyde [33,34]. This may require a parallel screening of samples processed without the reducing agent. Therefore, the analysis of samples not treated with NaBH_3_CN is currently ongoing.

Another potential limitation of the work presented is the unknown contribution of bacterial cells in the oral-rinse samples collected. Future investigations should involve a quantitative analysis of bacterial DNA contaminating the samples and evaluation of the use of antibacterial rinses before sample collection. Finally, only three adducts identified in our screening experiment were fully characterized by comparison with internal standards. Further studies will allow the full characterization of the other DNA modifications included in our targeted method, with priority given to crosslink DNA adducts detected in the oral DNA samples.

## 5. Conclusions

In conclusion, our improved DNA adductomic approach allowed the profiling of acetaldehyde-derived DNA adducts in vitro and the creation of a targeted method for the quantitation of the three major acetaldehyde-derived DNA adducts and screening of the 19 other putative DNA adducts. We report for the first time the detection of multiple DNA adducts in volunteers exposed to a specific alcohol dose and the characterization and quantitation of two new acetaldehyde-derived DNA adducts, *N*^4^-ethyl-dC, and *N*^6^-ethyl-dA.

## Figures and Tables

**Table 1:** Precursor ion (*m*/*z*), MS^2^ and MS^3^ spectra base peaks for each putative DNA adduct (MS^2^ and MS^3^ spectra are reported in the Supplementary Information (SI), together with the hypothesized structures) and presence in literature. Data were obtained analyzing DNA incubated with acetaldehyde (5 mmol).

Precursor Ion (*m*/*z*)	MS^2^ Base Peak (*m*/*z*)	MS^3^ Base Peak (*m*/*z*)	Previously Reported (Y/N)
256.1292	140.0817	95.0240	N
272.1239	156.0766	95.0604	N
280.1405	164.0930	136.0618	N
296.1357	180.0878	135.0300	Y [8]
296.1357	180.0878	145.0508	N
298.1395	182.0922	112.0506	N
306.1560	190.1086	136.0616	N
313.1399	197.0918	127.0502	N
322.1492	206.1036	162.0773	N
324.1666	208.1189	148.0616	N
326.1706	210.1225	138.0661	N
338.1459	222.0982	135.0300	Y [8]
340.1615	224.1140	135.0300	Y [7]
340.1615	224.1140	136.0506	N
342.1653	226.1181	112.0504	N
366.1773	250.1293	180.0880	N
382.1727	266.1241	178.0722	Y [8]
481.2041	370.1601	112.0505	N
505.2161	278.1244	112.0505	N
521.2102	178.0723	112.0505	N
587.2320	355.1374	204.0878	Y [8]
589.2490	473.1998	195.0989	Y [8]

**Table 2:** For each ion monitored, the average of the AUC was calculated in nonexposed and exposed oral-cell DNA and normalized by the ISs AUC and µmol of dG. The adducts that were only present after exposure are labelled as “+”, while those present in samples before and after exposure were labelled as “*”, when the levels increased significantly after exposure (*p* ≤ 0.05).

[M + H]+	Average Non-Exposed (AUC/µmol dG)	Average Exposed (AUC/µmol dG)	
272.1240	30.8	2520	*
256.1292 (*N*^4^-ethyl-dC)	2.66	392	*
280.1404 (*N*^6^-ethyl-dA)	0.235	5.15	*
296.1353 (*N*^2^-ethyl-dG)	43.2	6930	*
296.1353	1.15	93.5	*
298.1397	2.10	129	
306.1560	-	-	
313.1394	-	2.48	**+**
322.1509	40.8	1410	*
324.1666	0.0001	12.3	
326.1710	-	-	
338.1458	-	183	**+**
340.1615	-	-	
340.1615	0.561	32.7	
342.1659	-	47.4	**+**
366.1771	1.39	43.5	*
382.172	81.0	10,800	*
481.2041	-	-	
505.2153	-	1.06	**+**
521.2102	-	1.94	**+**
587.2320	-	-	
589.2477	-	0.533	**+**

* *p* ≤ 0.05, + only in exposed.

## Data Availability

The data presented in this study are available in the article and Supplementary Material.

## Supplementary Materials

The following are available online at https://www.mdpi.com/2218-273X/11/3/366/s1. Figure S1: Structures of isotopically labelled standards used for method testing: [^15^N_5_]*N*^2^-ethyl -dG, 2: [^15^N_5_]*N*^6^-methyl –dA, 3: [^15^N_5_]8-OH-PdG, 4: [D_4_]*O*^6^-POB-dT, 5: [D_4_]*O*^6^-POB-dG, and 6: [D_4_]*O*^6^-PHB-dG). Figure S2. Sample mass spectrum acquired with a DDA-CNL/MS^3^ and mass ranges (red lines) chosen for four *m*/*z* windows GPF test (197–310, 305–380, 375–450, and 445–750). Figure S3. Concentration-dependent formation of adducts (from the left to the right *N*^4^-ethyl-dC, *N*^6^-ethyl-dA, and *N*^2^-ethyl-dG) in the reaction of acetaldehyde with CT-DNA. Data are based on area of each adduct peak as determined by DDA-CNL/MS^3^. Figure S4. ^1^H (top) and ^13^C (bottom) NMR spectra of *N*^6^-ethyl-dA. Figure S5. ^1^H (top) and ^13^C (bottom) NMR spectra of [D_5_]*N*^6^-ethyl-dA. Figure S6. ^1^H (top) and ^13^C (bottom) NMR spectra of *N*^4^-ethyl-dC. Figure S7. ^1^H (top) and ^13^C (bottom) NMR spectra of [D_5_]*N*^4^-ethyl-dC. Figure S8. COSY (top) and HSQC (bottom) NMR spectra of *N*^6^-ethyl-dA. Figure S9. COSY (top) and HSQC (bottom) NMR spectra of [D_5_]*N*^6^-ethyl-dA. Figure S10. COSY (top) and HSQC (bottom) NMR spectra of *N*^4^-ethyl-dC. Figure S11. COSY (top) and HSQC (bottom) NMR spectra of [D_5_]*N*^4^-ethyl-dC. Table S1. *N*^4^-ethyl-dC, D_5_-*N*^4^-ethyl-dC, *N*^6^-ethyl-dA, and D_5_-*N*^6^-ethyl-dA proton and carbon NMR signals in D_5_-DMSO. Table S2. 3′, 5′-bis-O-acetyl-2′-deoxyuridine, 4-chloro-1-N-(3′, 5′-bis-O-acetyl-2′-deoxyribosyl)-2-pyrimidinoneproton and carbon NMR signals in CDCl_3_. Figure S12. MS^2^ spectra of *N*^4^-ethyl-dC (256.1292 *m*/*z*). Figure S13. MS^2^ spectra of [D_5_]*N*^4^-ethyl-dC (261.1605 *m*/*z*). Figure S14. MS^2^ spectra of *N*^6^-ethyl-dA (280.1404 *m*/*z*). Figure S15. MS^2^ spectra of [D_5_]*N*^6^-ethyl-dA (285.1718 *m*/*z*). Figure S16. MS^2^ spectra of *N*^2^-ethyl-dG (296.1353 *m*/*z*). Figure S17. MS^2^ spectra of [^15^N_5_]*N*^2^-ethyl-dG (301.1205 *m*/*z*). Figure S18. Relationship between added amount and measure amount in CT-DNA of A: *N*^2^-ethyl-dG, B: *N*^6^-ethyl-dA, and C: *N*^4^-ethyl-dC. Table S3. Peak area of ethyl-adducts in alcohol-exposed and nonexposed oral cells. Table S4. Average (fmol/µmol dG), standard deviation (STD), and *p*-value of ethyl-adducts in alcohol-exposed and nonexposed oral cells.
