# Supplementary material for: Identification of New Markers of Alcohol-Derived DNA Damage in Humans
Source: Biomolecules. 2021 Feb 27;11(3):366. doi: 10.3390/biom11030366 (PMC7997542; doi:10.3390/biom11030366)
Supplement: Supplementary file 1 [file biomolecules-11-00366-s001.pdf]

Supporting Information

Identification of new markers of alcohol-derived DNA damage in humans.

Valeria Guidolin[1,2], Erik S. Carlson[2], Andrea Carra'[2], Peter W. Villalta[2], Laura A. Maertens[2], Stephen S. Hecht[2] and Silvia Balbo[1,2*]

1 Division of Environmental Health Sciences, University of Minnesota, Minneapolis, MN 55455, USA; guido019@umn.edu (V.G.);

2 Masonic Cancer Center, University of Minnesota, 2231 6th Street SE, Minneapolis, MN 55455, USA; erik_carlson@fas.harvard.edu (E.S.C.), andrea.carra.101184@gmail.com (A.C.), villa001@umn.edu (P.W.V.), maert006@umn.edu (L.A.M.), hecht002@umn.edu (S.S.H.);

* Correspondence: balbo006@umn.edu (S.B.);

Received: date; Accepted: date; Published: date

Contents

Evaluation of the removal of $NaBH_3CN$ and acetaldehyde from the sample......2

Quantification of dG by LC/UV......2

Human samples and alcohol study......2

Exclusion List from LC-MS methods......3

DDA-CNL/MS^3 gas-phase fractionation method testing......4

MS^2, MS^3 spectra and possible structures of DNA adducts detected using LC-HRMS in GPF-DDA-CNL/MS^3 scan mode in acetaldehyde-exposed CT-DNA, after $NaBH_3CN$ treatment.......5

Concentration-dependent DNA adduct generation......27

NMR characterization of *N*^{6}-ethyl-dA, [D_5]*N*^{6}-ethyl-dA, *N*^{4}-ethyl-dC and [D_5]*N*^{4}-ethyl-dC......28

HRMS characterization of *N*^{6}-ethyl-dA, [D_5]*N*^{6}-ethyl-dA, *N*^{4}-ethyl-dC and [D_5]*N*^{4}-ethyl-dC......37

Quantitation of N^4-ethyl-dC, N^6-ethyl-dA and N^2-ethyl-dG in human oral cells..41

Evaluation of the removal of $NaBH_3CN$ and acetaldehyde from the sample

$NaBH_3CN$ may negatively impact enzyme activity, yielding low hydrolysis rates. For this reason, 3 different protocols for the removal of $NaBH_3CN$ were evaluated. In the first protocol, samples were filtered using double filtration membranes (Amicon Ultra, 30K cut-off) before addition of the enzymes. In the second protocol, the DNA was first precipitated with cold IPA and subsequently filtered using double filtration membranes (Amicon Ultra, 30K cut-off). Finally, in the third protocol, the DNA was precipitated with cold 100% IPA and washed with 75% cold IPA and 100% cold IPA. DNA recovery and levels of acetaldehyde and $NaBH_3CN$ left in the sample were considered as criteria for final protocol selection. Acetaldehyde removal from the sample was evaluated by measuring the level of acetaldehyde in the solution from which the DNA was extracted. Acetaldehyde was derivatized with DNPH [1]. Subsequently, it was quantified by UPLC. The instrument was operated at 40°C, performing a multi-step gradient using H_2O and MeOH as mobile phase A and B, respectively, with a flow rate of 10 µL/min. The autosampler was set up to inject 1 µL of sample. The eluent was held at 2% B for 5 min, increased to 30% B in 5 min followed by a 2 min hold, then increased to 50% in 8 min followed by a 5 min hold, further increased to 70% B in 5 min followed by a 5 min hold, increased to 80% in 4 min followed by a 4 min hold, and finally increased to 95% B in 1 min. This step was followed by a wash at 95% B for 5 min and re-equilibration of the column. Detection was accomplished using the UV-Vis detector set at 360 nm. $NaBH_3CN$ removal from the sample was evaluated by drying and weighing the vial in which the DNA was precipitated or filtered. DNA recovery was assessed by absorbance using a UV/Vis-spectrophotometer. (Eppendorf, Hauppauge, NY, USA). The spectrophotometer was used in the absorbance optical mode, monitoring the 260 nm and the 280 nm wavelengths. The third protocol, in which the DNA was precipitated with cold 100% IPA and then washed with 75% cold IPA and 100% cold IPA, was demonstrated to be the most effective for the parameters considered leading to a complete removal of $NaBH_3CN$, acetaldehyde and full DNA recovery.

Quantification of dG by LC/UV

The chromatographic separation was performed on a C18-column (0.3 x 100 mm, 100 Å, 2 µm Acclaim-Thermo Scientific, Waltham, MA) operating at 40 °C with a flow rate of 5 µL/min. The mobile phase consisted of H_2O and MeOH. The autosampler was set to inject 1 µL of sample. The elution program involved an isocratic step at 2 % MeOH (5 min), followed by a linear gradient of 1.5 %/min (25 min, MeOH) and a second isocratic step at 95 % of MeOH (5 min). At the end of the elution program, the LC-system was equilibrated at isocratic conditions (2 % MeOH) for 20 min. The UV detector operated at 10 Hz, probing the absorbance of dG at 254 nm wavelength.

Human samples and alcohol study

Healthy volunteers between the ages of 21 and 50 years old, were recruited to participate in an alcohol hangover pilot study. Participants had to be non-smokers, had not to have used any tobacco products (cigarettes, cigars, pipe, or smokeless tobacco), marijuana, or e-cigarettes in the last six months and not be of East Asian ethnicity or descent (Japanese, Chinese, and Koreans) [2,3]. They also had to drink alcohol on a regular basis, but could not have a history of alcohol abuse or a family history of alcoholism. On average, they had to consume at least one alcoholic drink per week and had consumed four drinks over a 4 h period

in the last six months. Since participants were going to be given a dose of alcohol that would lead to legal intoxication, it was necessary that they have experience consuming a similar dose of alcohol prior to their participation in the study. Participants also had to be of stable mental and physical health, could not have insulin-dependent diabetes, could not be taking any medications that interacted with alcohol, and could not have lesions or cuts in their mouth or other periodontal issues. They also had to agree to refrain from the use of street drugs and the consumption of alcohol outside of the study for the five days duration of the study. Urine samples were used to perform pregnancy tests for female participants to ensure they were not pregnant.

Eligible participants were asked to attend an orientation visit on Day 1 of the study, where they filled out questionnaires regarding their demographics, medical/medication use, alcohol use, tobacco use history, and typical hangover symptoms. They also completed a battery of cognitive tests (Trail A, Trail B, 2 Letter Search, Arithmetic, Zero N-back, 2 N-back) twice. Participants were provided a pre-pasted toothbrush to brush their teeth 20 min prior to giving an oral rinse sample. Oral rinse samples were collected by swishing 15 mL of 0.9% saline solution from cheek to cheek 30 times and spitting the wash into a collection tube. Blood and urine samples were also collected, and blood pressure, heart rate and weight were measured. The weight and sex of the participants were used to calculate their alcohol dose (Figure S1).

Participants had to refrain from drinking alcohol for Days 2 and 3, and then report to the clinic on the morning of Day 4 for a 14-h session. Participants were asked to brush their teeth prior to arriving at the clinic and to only have water after brushing. Baseline samples of saliva, oral rinse, urine, blood, and cheek brushing were collected, and the battery of cognitive tests was performed. The dose was a mixture of vodka/tonic water (1:2 w/w) containing 10 mL of lime juice. Participants were given 1 h to drink the dose but were encouraged to drink it in about 45 min because the longer participants took to consume their dose the less likely they were to reach the target blood alcohol concentration (0.11%). However, participants were never encouraged to drink faster than what they felt was comfortable. The alcohol dose was administered to 18 participants, and three participants received a placebo drink of tonic water and lime juice.

One h after finishing their dose of alcohol, participants were given a breathalyzer test to measure their blood alcohol concentration (BAC) and were asked to provide a saliva sample. Cheek brushing samples were collected at 2 and 6 h post alcohol dose, and blood and urine samples were collected at 2, 6, 12, and 24 h post dose. Oral rinse samples were collected at 2, 6, 8, 12, 16, 24, and 36 h after dosing. Participants were given a hangover self-assessment and the final battery of cognitive tests at 12 h post dose. At this time they were also given a breathalyzer test to confirm that their blood alcohol concentration had returned to zero and that they could therefore be released from the clinic.

Exclusion List from LC-MS methods

Ions present in the exclusion list used for the mass spectrometry method for screening DNA adducts: 228.0979, 243.0975, 245.1244, 250.0798, 252.1091, 260.1241, 265.0795, 266.0538, 268.104, 269.1357, 274.0911, 281.0534, 285.1306, 290.065, 290.086, 306.0599, 356.1677, 361.1231, 371.1674, 376.1228, 377.0971, 380.179, 385.1344386.1671, 391.1225, 392.0967, 395.1786, 396.1739, 400.134, 401.1083, 401.1293, 404.1902, 407.0964, 409.1456, 411.1735, 416.108, 416.1289, 417.1032, 419.1535, 420.1851, 425.1195, 425.1405, 432.1029, 436.18, 441.1144, 441.1354, 455.1885, 457.1094, 470.1881, 470.1881, 472.215, 477.1704, 479.1997, 479.1997, 485.1878, 485.1878, 487.2147, 492.1701, 493.1444, 494.1994, 494.1994, 495.1946, 495.1946, 496.2263, 501.1817, 502.2144, 503.211, 503.211, 507.1698, 508.144, 510.1943, 510.1943, 511.2259, 512.2212, 516.1813, 517.1556, 517.1766, 519.2059, 519.2059, 520.2375, 523.1437, 525.1929, 527.2208, 532.1553, 532.1762, 533.1505, 535.2008, 536.2324, 541.1668, 541.1878, 548.1502, 552.2273, 557.1617, 557.1827, 573.1567.

DDA-CNL/MS³ gas-phase fractionation method testing

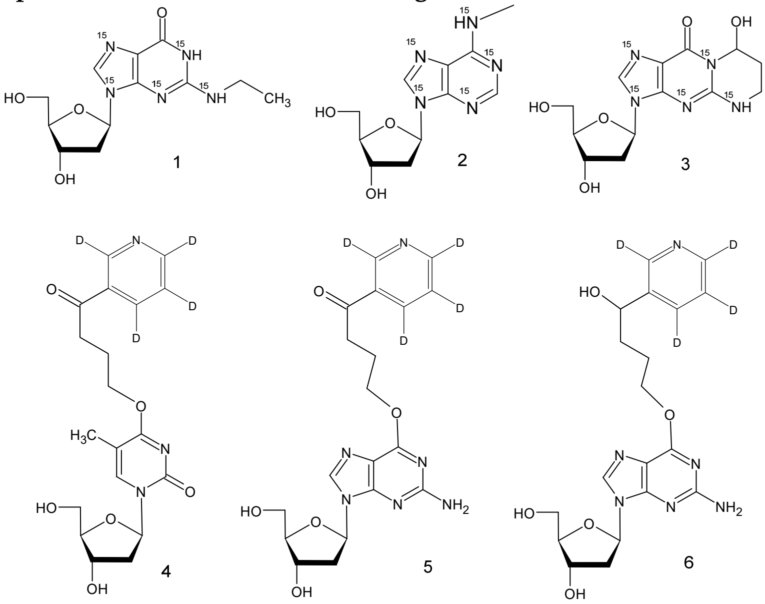

Figure S1. Structures of isotopically-labelled standards used for method testing: [$^{15}N_5$]N^2-ethyl -dG, 1: [$^{15}N_5$]N^6-methyl–dA, 2: [$^{15}N_5$]8-OH-PdG, 3: [D_4]O^4-POB-dT, 4: [D_4]O^6-POB-dG, 5: [D_4]O^6-PHB-dG, 6.

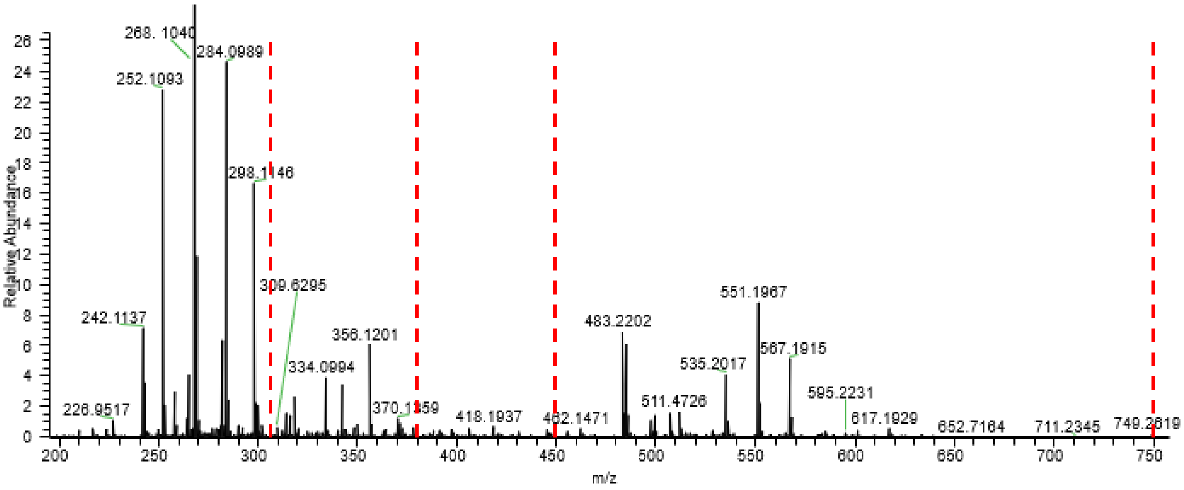

Figure S2. Sample mass spectrum acquired with a DDA-CNL/MS³ and mass ranges (red lines) chosen for 4 *m/z* windows GPF test (*m/z* 197-310, 305- 380, 375-450, 445-750).

MS[2], MS[3] spectra and possible structures of DNA adducts detected using LC-HRMS in GPF-DDA-CNL/MS[3] scan mode in acetaldehyde-exposed CT-DNA, after $NaBH_3CN$ treatment.

Only the structures of *N*[4]-ethyl-dC, *N*[6]-ethyl-dA, and *N*[2]-ethyl-dG were confirmed by comparison to synthetic standards in this study. The other structures are proposals only and require confirmation by comparison to independently synthesized standards and/or further spectral data.

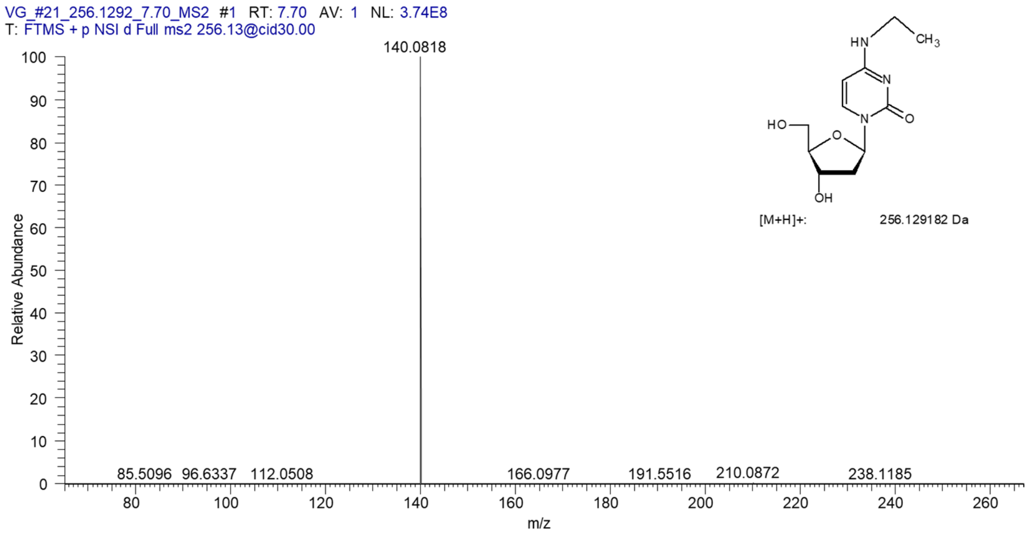

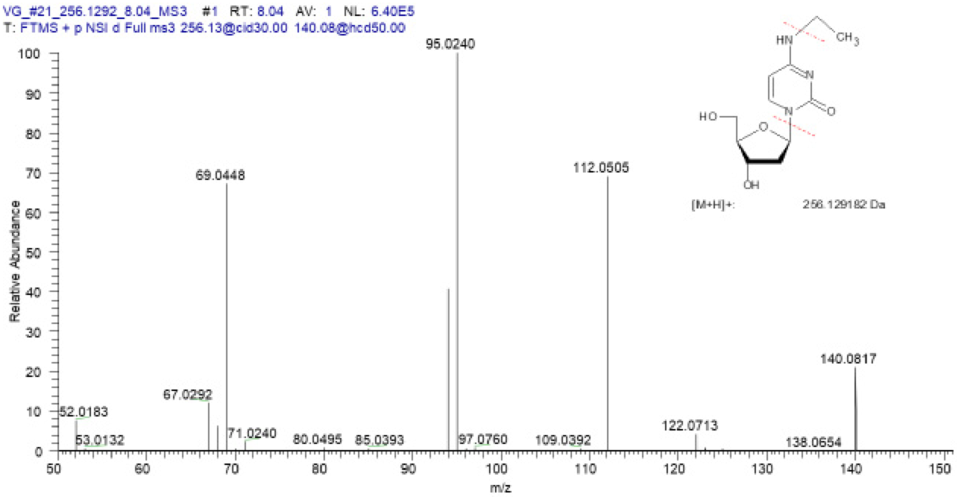
VG_#21_256.1292_8.04_MS3 #1 RT: 8.04 AV: 1 NL: 6.40E5
T: FTMS + p NSI d Full ms3 256.13@cid30.00 140.08@hcd50.00
Relative Abundance
m/z
95.0240
69.0448
112.0505
140.0817
67.0292
52.0183
53.0132
71.0240
80.0495
85.0393
97.0760
109.0392
122.0713
138.0654
[M+H]+: 256.129182 Da

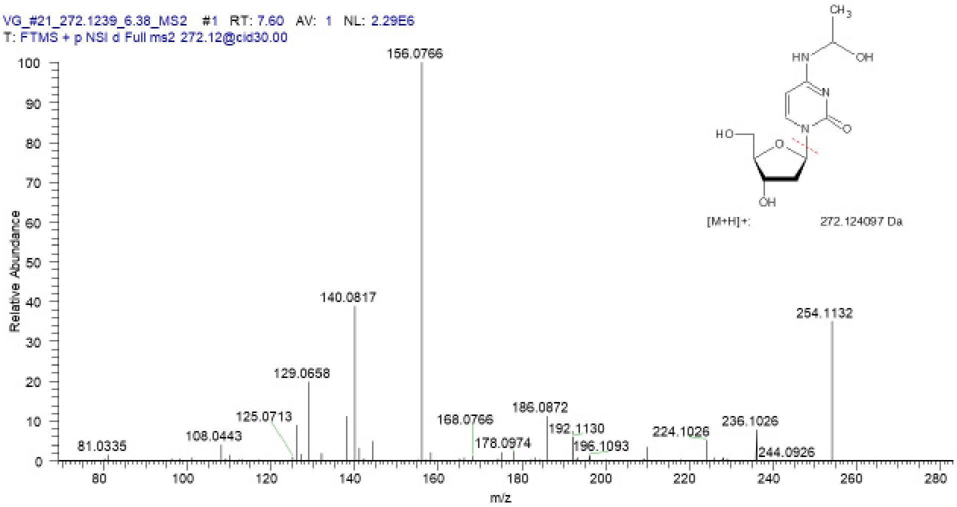
VG_#21_272.1239_6.38_MS2 #1 RT: 7.60 AV: 1 NL: 2.29E6
T: FTMS + p NSI d Full ms2 272.12@cid30.00
Relative Abundance
m/z
156.0766
140.0817
254.1132
129.0658
125.0713
108.0443
81.0335
168.0766
186.0872
178.0974
192.1130
196.1093
224.1026
236.1026
244.0926
[M+H]+: 272.124097 Da

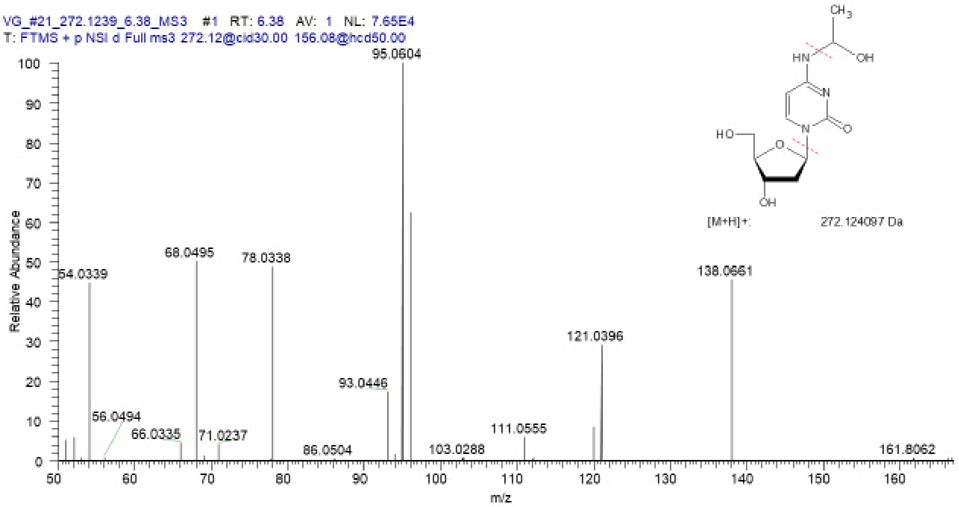
VG_#21_272.1239_6.38_MS3 #1 RT: 6.38 AV: 1 NL: 7.65E4
T: FTMS + p NSI d Full ms3 272.12@cid30.00 156.08@hcd50.00
Relative Abundance
m/z
95.0604
54.0339
68.0495
78.0338
138.0661
121.0396
93.0446
56.0494
66.0335
71.0237
86.0504
103.0288
111.0555
161.8062
[M+H]+: 272.124097 Da

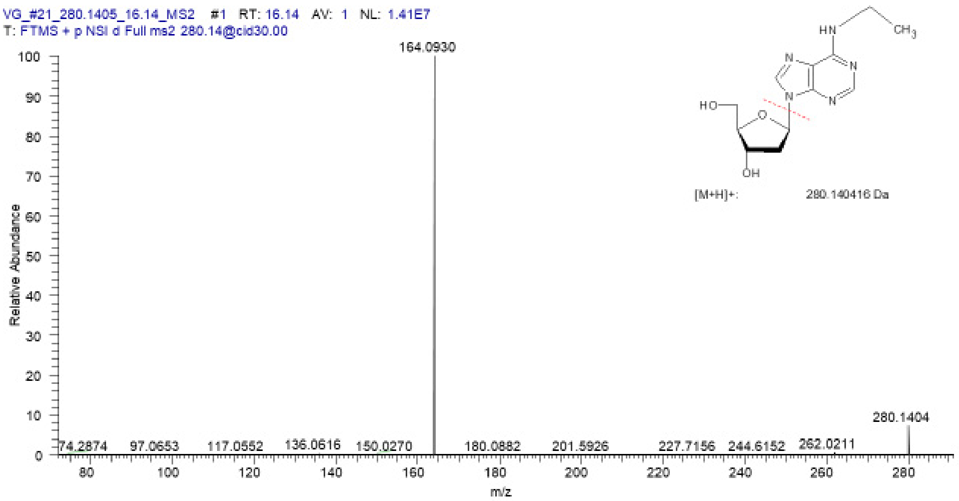
VG_#21_280.1405_16.14_MS2 #1 RT: 16.14 AV: 1 NL: 1.41E7
T: FTMS + p NSI d Full ms2 280.14@cid30.00
Relative Abundance
m/z
164.0930
74.2874
97.0653
117.0552
136.0616
150.0270
180.0882
201.5926
227.7156
244.6152
262.0211
280.1404
[M+H]+: 280.140416 Da

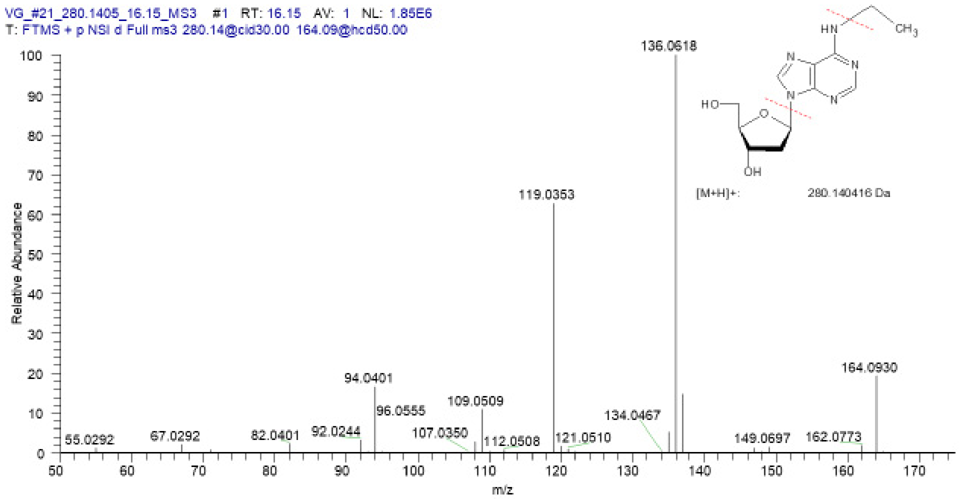
VG_#21_280.1405_16.15_MS3 #1 RT: 16.15 AV: 1 NL: 1.85E6
T: FTMS + p NSI d Full ms3 280.14@cid30.00 164.09@hcd50.00
Relative Abundance
m/z
136.0618
119.0353
94.0401
96.0555
109.0509
107.0350
112.0508
121.0510
134.0467
164.0930
149.0697
162.0773
55.0292
67.0292
82.0401
92.0244
HN
CH3
N
HO
O
OH
[M+H]+:
280.140416 Da

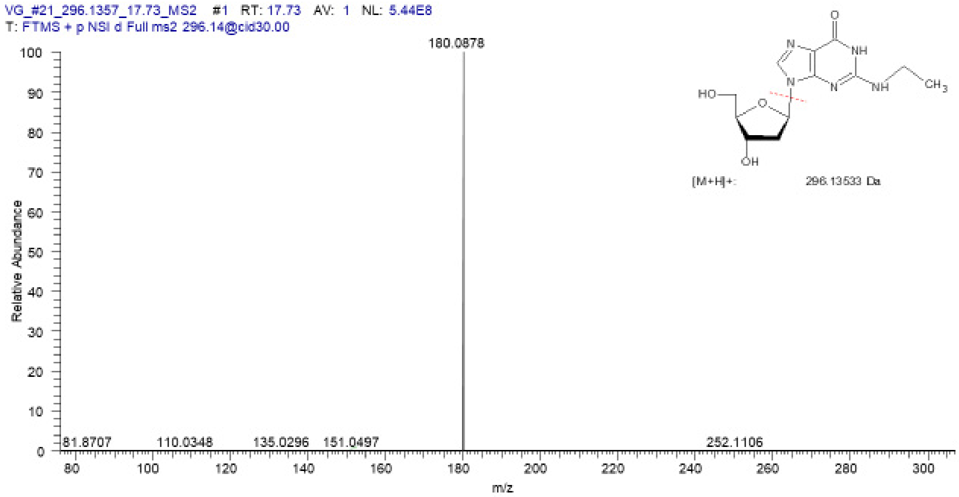
VG_#21_296.1357_17.73_MS2 #1 RT: 17.73 AV: 1 NL: 5.44E8
T: FTMS + p NSI d Full ms2 296.14@cid30.00
Relative Abundance
m/z
180.0878
81.8707
110.0348
135.0296
151.0497
252.1106
O
NH
NH
CH3
HO
OH
[M+H]+:
296.13533 Da

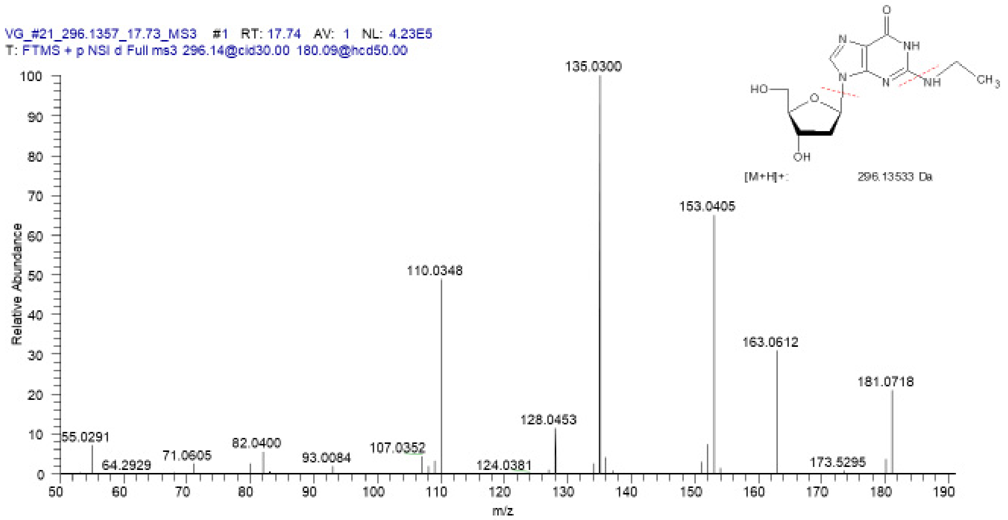
VG_#21_296.1357_17.73_MS3 #1 RT: 17.74 AV: 1 NL: 4.23E5
T: FTMS + p NSI d Full ms3 296.14@cid30.00 180.09@hcd50.00
Relative Abundance
m/z
135.0300
153.0405
110.0348
163.0612
181.0718
128.0453
55.0291
82.0400
107.0352
71.0605
93.0084
64.2929
124.0381
173.5295
[M+H]+: 296.13533 Da

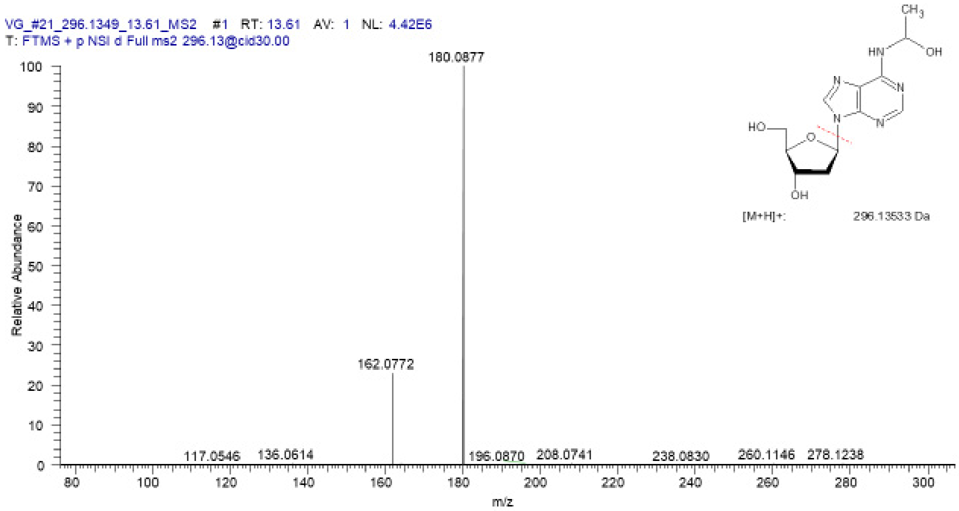
VG_#21_296.1349_13.61_MS2 #1 RT: 13.61 AV: 1 NL: 4.42E6
T: FTMS + p NSI d Full ms2 296.13@cid30.00
Relative Abundance
m/z
180.0877
162.0772
117.0546
136.0614
196.0870
208.0741
238.0830
260.1146
278.1238
[M+H]+: 296.13533 Da

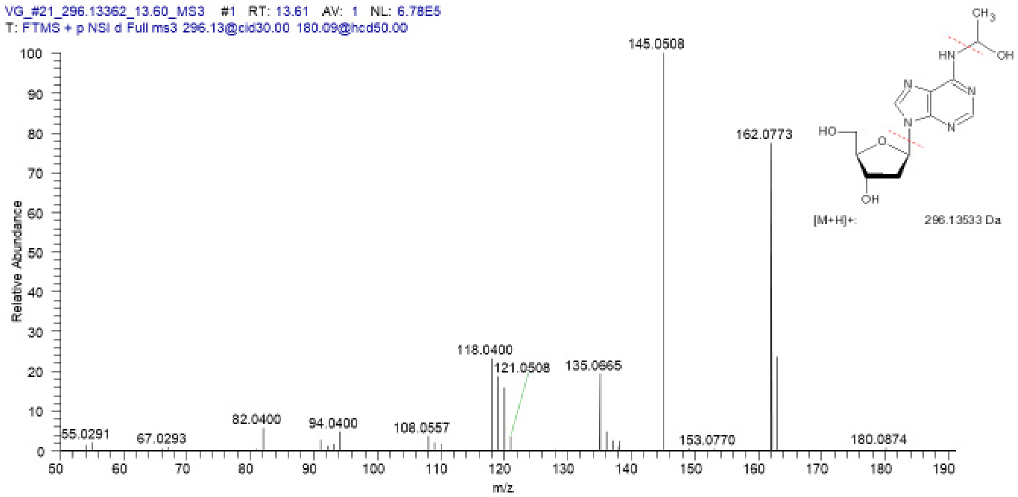
VG_#21_296.13362_13.60_MS3 #1 RT: 13.61 AV: 1 NL: 6.78E5
T: FTMS + p NSI d Full ms3 296.13@cid30.00 180.09@hcd50.00
145.0508
162.0773
118.0400
121.0508
135.0665
82.0400
94.0400
108.0557
55.0291
67.0293
153.0770
180.0874
Relative Abundance
m/z
[M+H]+: 296.13533 Da

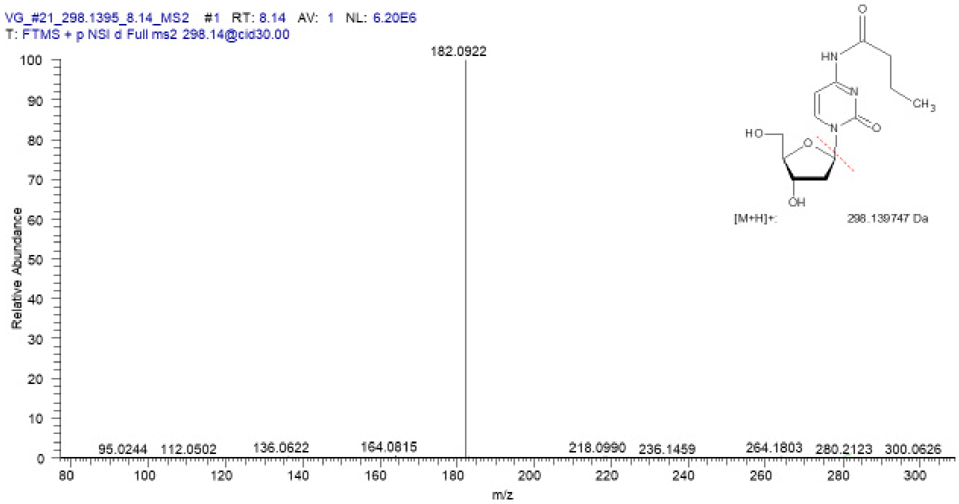
VG_#21_298.1395_8.14_MS2 #1 RT: 8.14 AV: 1 NL: 6.20E6
T: FTMS + p NSI d Full ms2 298.14@cid30.00
182.0922
95.0244
112.0502
136.0622
164.0815
218.0990
236.1459
264.1803
280.2123
300.0626
Relative Abundance
m/z
[M+H]+: 298.139747 Da

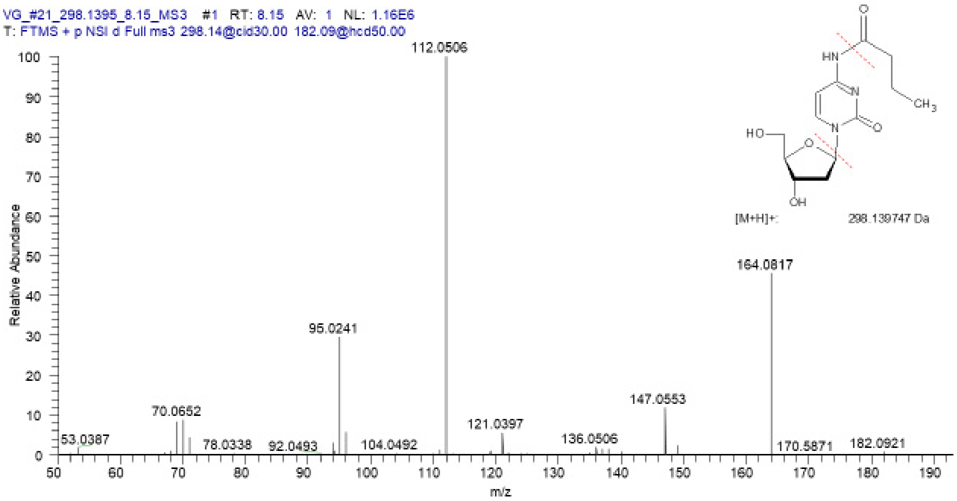
VG_#21_298.1395_8.15_MS3 #1 RT: 8.15 AV: 1 NL: 1.16E6
T: FTMS + p NSI d Full ms3 298.14@cid30.00 182.09@hcd50.00
Relative Abundance
m/z
112.0506
164.0817
95.0241
147.0553
70.0652
121.0397
136.0506
53.0387
78.0338
92.0493
104.0492
170.5871
182.0921
HO
O
N
N
O
HN
O
CH3
OH
[M+H]+:
298.139747 Da

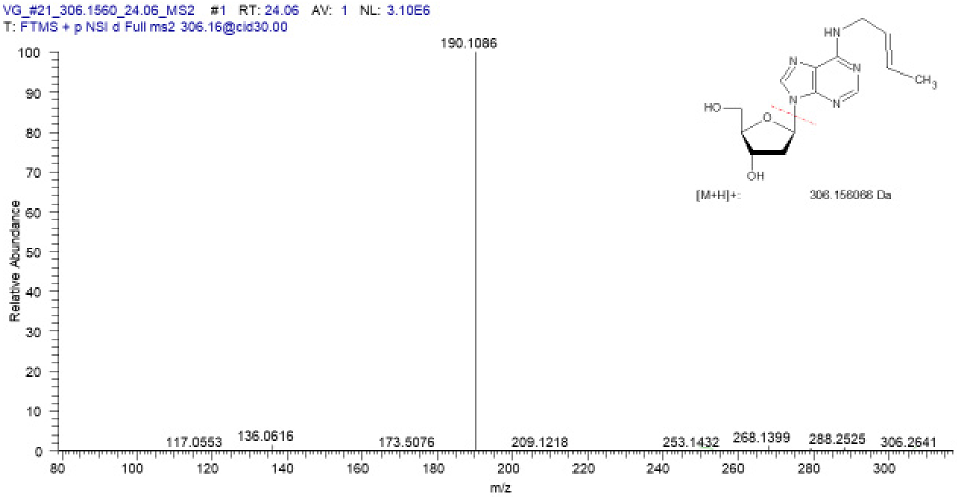
VG_#21_306.1560_24.06_MS2 #1 RT: 24.06 AV: 1 NL: 3.10E6
T: FTMS + p NSI d Full ms2 306.16@cid30.00
Relative Abundance
m/z
190.1086
117.0553
136.0616
173.5076
209.1218
253.1432
268.1399
288.2525
306.2641
HN
N
N
N
N
CH3
HO
O
OH
[M+H]+:
306.156066 Da

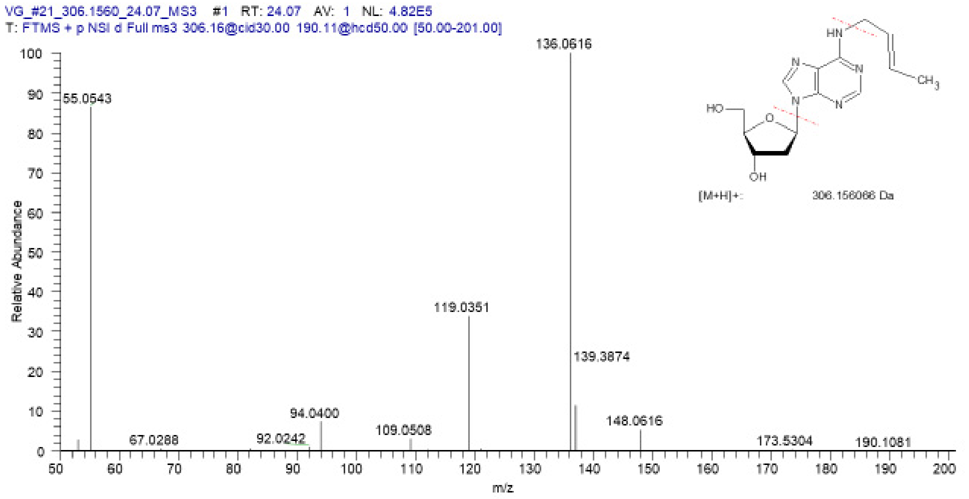

VG_#21_306.1560_24.07_MS3 #1 RT: 24.07 AV: 1 NL: 4.82E5
T: FTMS + p NSI d Full ms3 306.16@cid30.00 190.11@hcd50.00 [50.00-201.00]
136.0616
55.0543
119.0351
139.3874
94.0400
148.0616
109.0508
67.0288
92.0242
173.5304
190.1081
Relative Abundance
m/z
HN
N
CH3
HO
O
OH
[M+H]+: 306.156066 Da

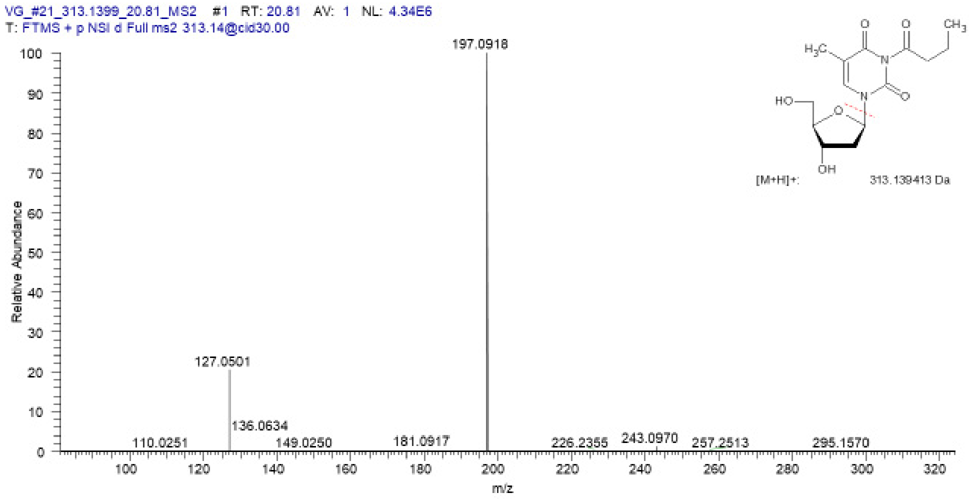

VG_#21_313.1399_20.81_MS2 #1 RT: 20.81 AV: 1 NL: 4.34E6
T: FTMS + p NSI d Full ms2 313.14@cid30.00
197.0918
127.0501
136.0634
110.0251
149.0250
181.0917
226.2355
243.0970
257.2513
295.1570
Relative Abundance
m/z
O
CH3
H3C
N
HO
OH
[M+H]+: 313.139413 Da

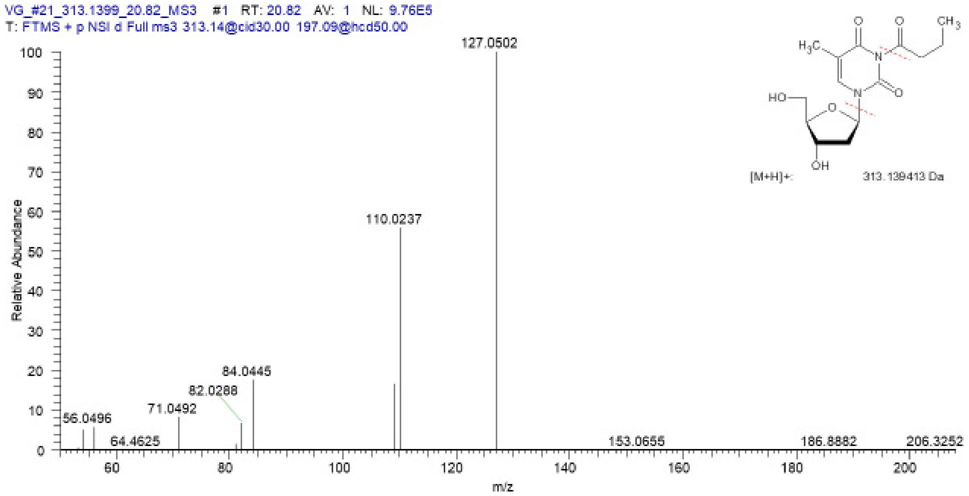
VG_#21_313.1399_20.82_MS3 #1 RT: 20.82 AV: 1 NL: 9.76E5
T: FTMS + p NSI d Full ms3 313.14@cid30.00 197.09@hcd50.00
127.0502
110.0237
84.0445
82.0288
71.0492
56.0496
64.4625
153.0655
186.8882
206.3252
Relative Abundance
m/z
[M+H]+: 313.139413 Da

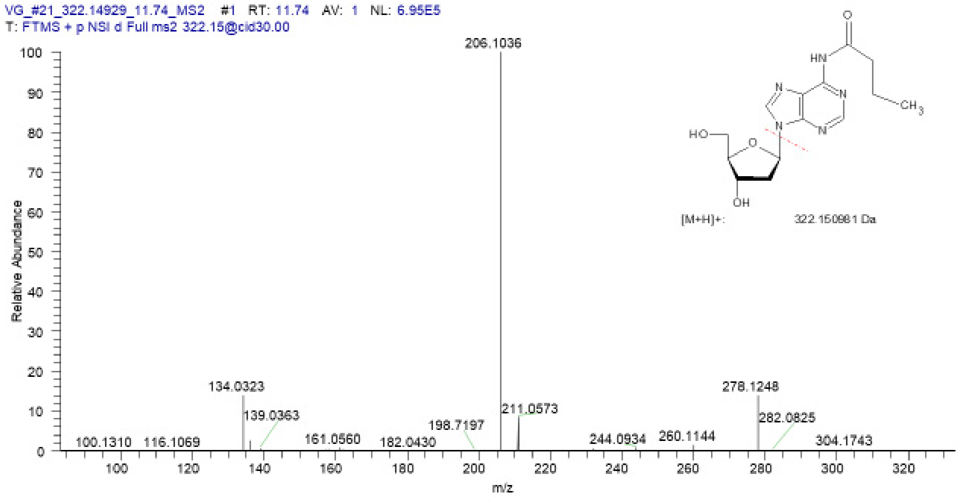
VG_#21_322.14929_11.74_MS2 #1 RT: 11.74 AV: 1 NL: 6.95E5
T: FTMS + p NSI d Full ms2 322.15@cid30.00
206.1036
134.0323
139.0363
278.1248
211.0573
282.0825
198.7197
100.1310
116.1069
161.0560
182.0430
244.0934
260.1144
304.1743
Relative Abundance
m/z
[M+H]+: 322.150981 Da

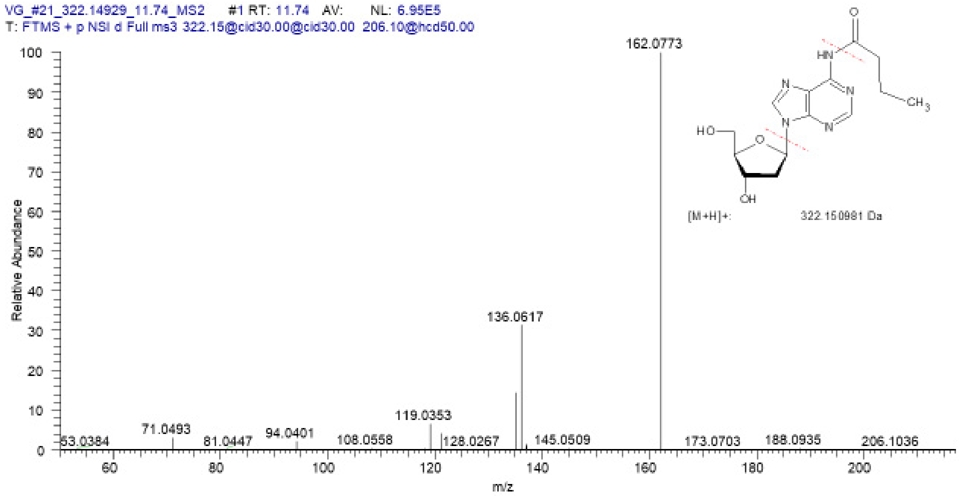

VG_#21_322.14929_11.74_MS2 #1 RT: 11.74 AV: NL: 6.95E5
T: FTMS + p NSI d Full ms3 322.15@cid30.00@cid30.00 206.10@hcd50.00
Relative Abundance
m/z
162.0773
136.0617
119.0353
71.0493
94.0401
53.0384
81.0447
108.0558
128.0267
145.0509
173.0703
188.0935
206.1036
[M+H]+:
322.150981 Da

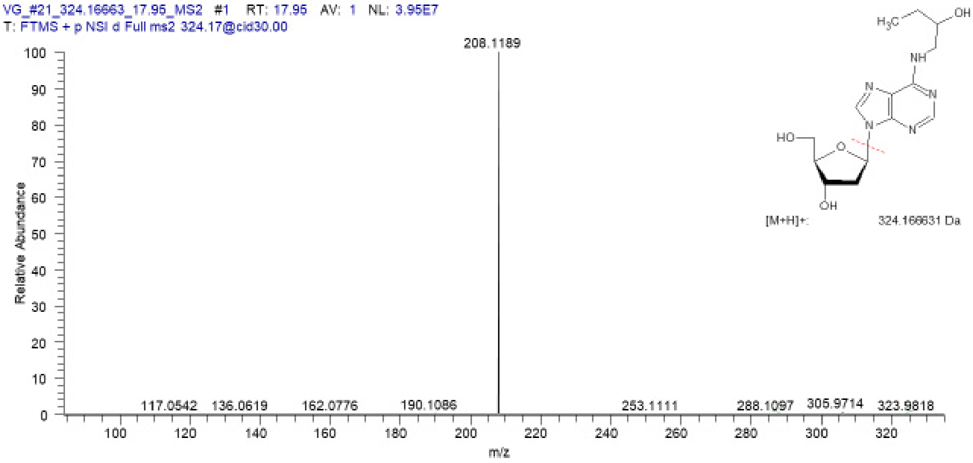

VG_#21_324.16663_17.95_MS2 #1 RT: 17.95 AV: 1 NL: 3.95E7
T: FTMS + p NSI d Full ms2 324.17@cid30.00
Relative Abundance
m/z
208.1189
117.0542
136.0619
162.0776
190.1086
253.1111
288.1097
305.9714
323.9818
[M+H]+:
324.166631 Da

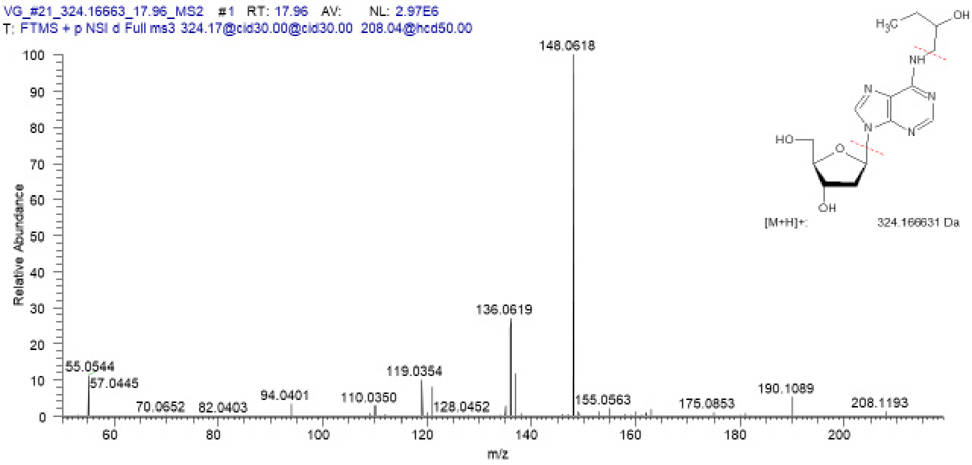
VG_#21_324.16663_17.96_MS2 #1 RT: 17.96 AV: NL: 2.97E6
T: FTMS + p NSI d Full ms3 324.17@cid30.00@cid30.00 208.04@hcd50.00
Relative Abundance
m/z
148.0618
136.0619
119.0354
55.0544
57.0445
70.0652
82.0403
94.0401
110.0350
128.0452
155.0563
175.0853
190.1089
208.1193
[M+H]+:
324.166631 Da

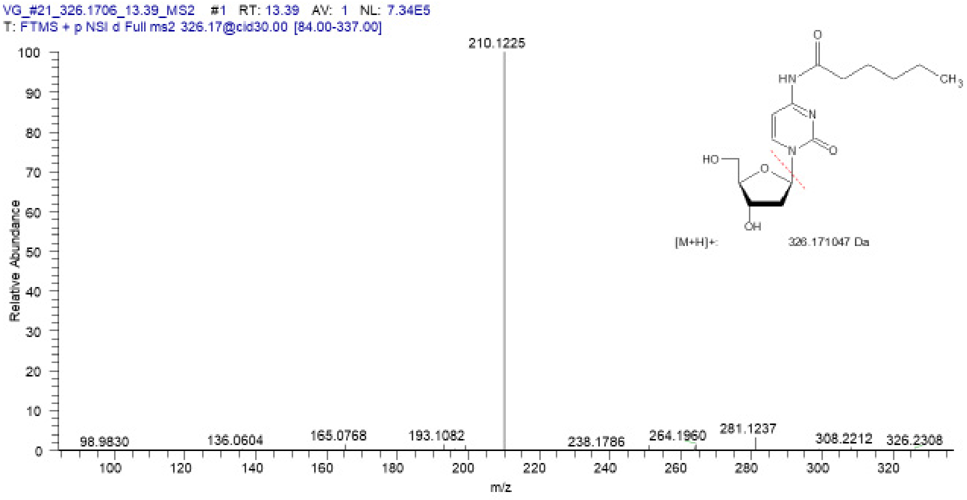
VG_#21_326.1706_13.39_MS2 #1 RT: 13.39 AV: 1 NL: 7.34E5
T: FTMS + p NSI d Full ms2 326.17@cid30.00 [84.00-337.00]
Relative Abundance
m/z
210.1225
98.9830
136.0604
165.0768
193.1082
238.1786
264.1960
281.1237
308.2212
326.2308
[M+H]+:
326.171047 Da

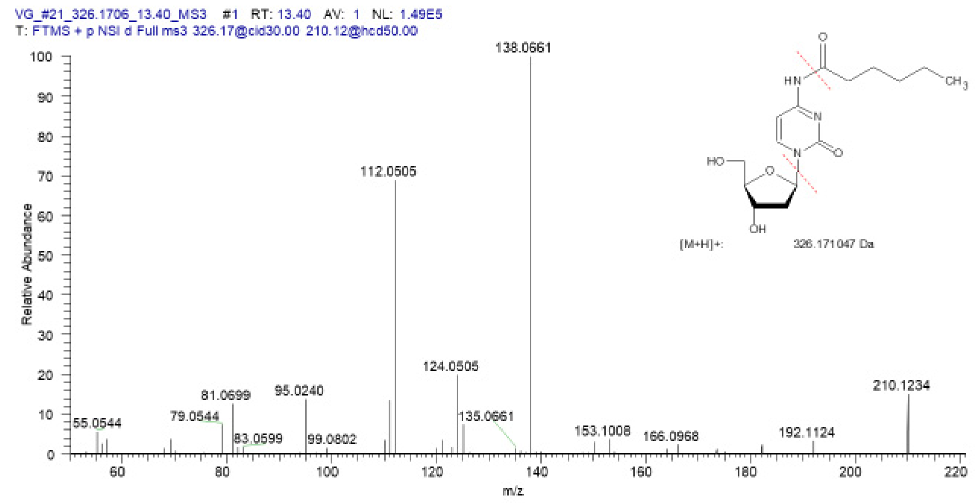
VG_#21_326.1706_13.40_MS3 #1 RT: 13.40 AV: 1 NL: 1.49E5
T: FTMS + p NSI d Full ms3 326.17@cid30.00 210.12@hcd50.00
138.0661
112.0505
124.0505
210.1234
81.0699
95.0240
55.0544
79.0544
83.0599
99.0802
135.0661
153.1008
166.0968
192.1124
Relative Abundance
m/z
[M+H]+: 326.171047 Da

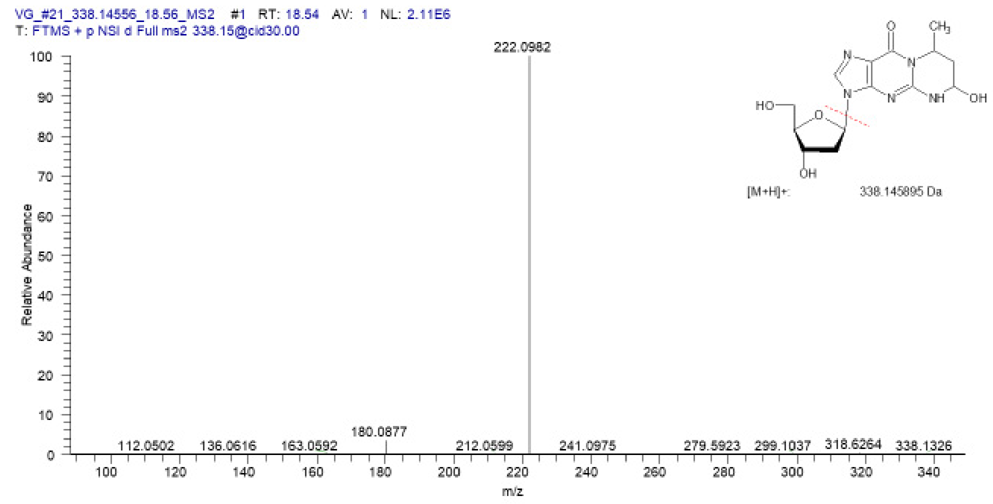
VG_#21_338.14556_18.56_MS2 #1 RT: 18.54 AV: 1 NL: 2.11E6
T: FTMS + p NSI d Full ms2 338.15@cid30.00
222.0982
180.0877
112.0502
136.0616
163.0592
212.0599
241.0975
279.5923
299.1037
318.6264
338.1326
Relative Abundance
m/z
[M+H]+: 338.145895 Da

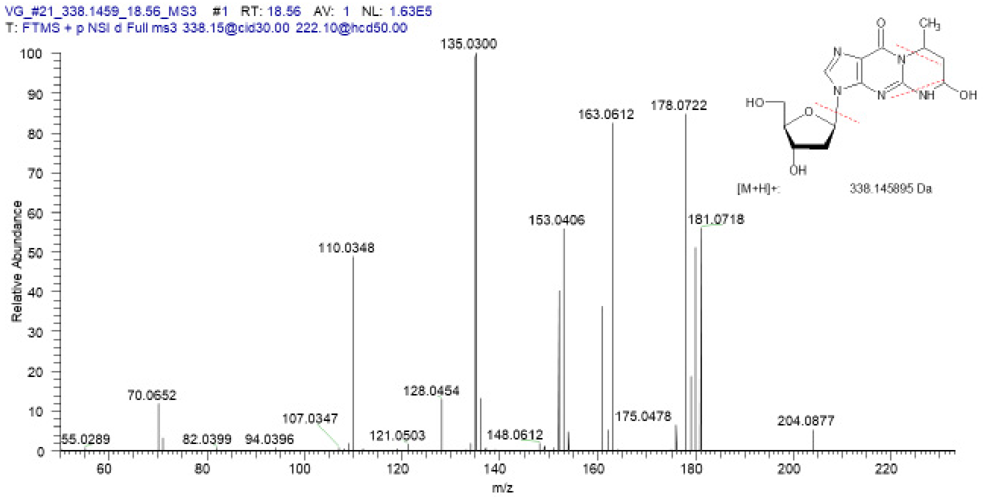
VG_#21_338.1459_18.56_MS3 #1 RT: 18.56 AV: 1 NL: 1.63E5
T: FTMS + p NSI d Full ms3 338.15@cid30.00 222.10@hcd50.00
Relative Abundance
m/z
135.0300
163.0612
178.0722
153.0406
181.0718
110.0348
70.0652
128.0454
107.0347
55.0289
82.0399
94.0396
121.0503
148.0612
175.0478
204.0877
[M+H]+:
338.145895 Da

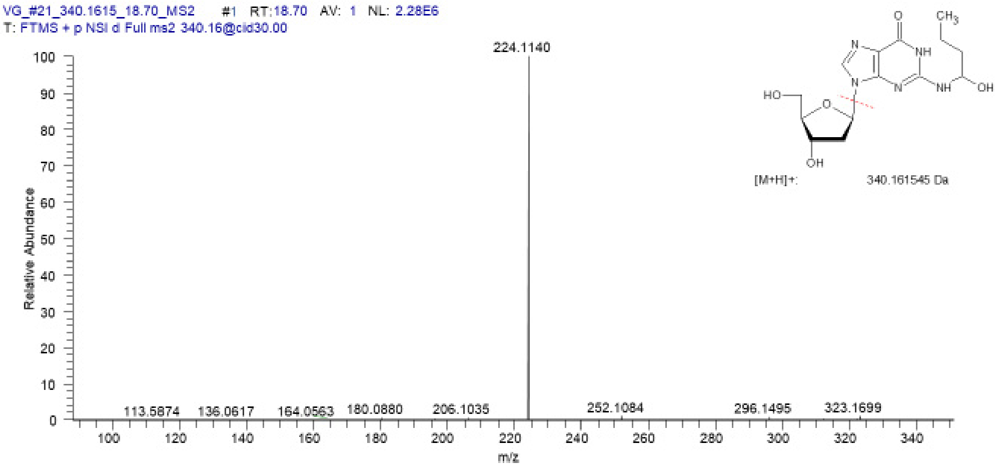
VG_#21_340.1615_18.70_MS2 #1 RT:18.70 AV: 1 NL: 2.28E6
T: FTMS + p NSI d Full ms2 340.16@cid30.00
Relative Abundance
m/z
224.1140
113.5874
136.0617
164.0563
180.0880
206.1035
252.1084
296.1495
323.1699
[M+H]+:
340.161545 Da

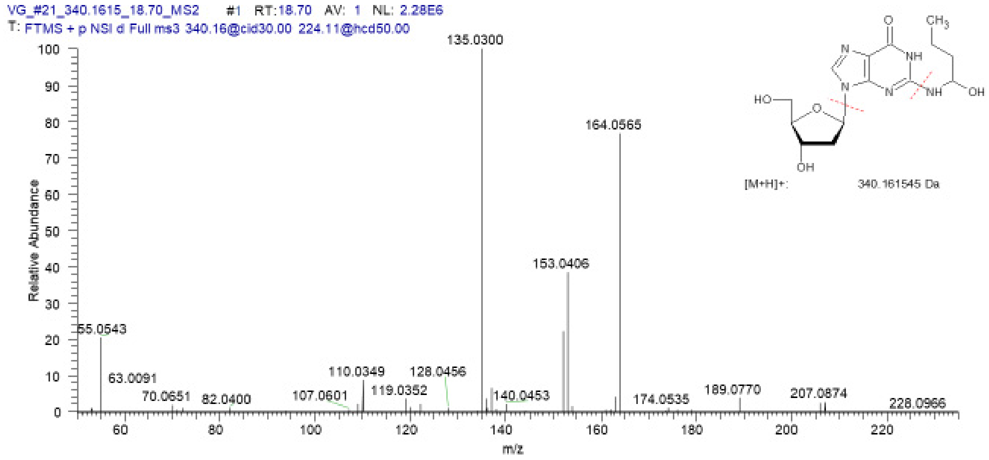
VG_#21_340.1615_18.70_MS2 #1 RT:18.70 AV: 1 NL: 2.28E6
T: FTMS + p NSI d Full ms3 340.16@cid30.00 224.11@hcd50.00
135.0300
164.0565
153.0406
55.0543
63.0091
70.0651
82.0400
107.0601
110.0349
119.0352
128.0456
140.0453
174.0535
189.0770
207.0874
228.0966
Relative Abundance
m/z
[M+H]+: 340.161545 Da

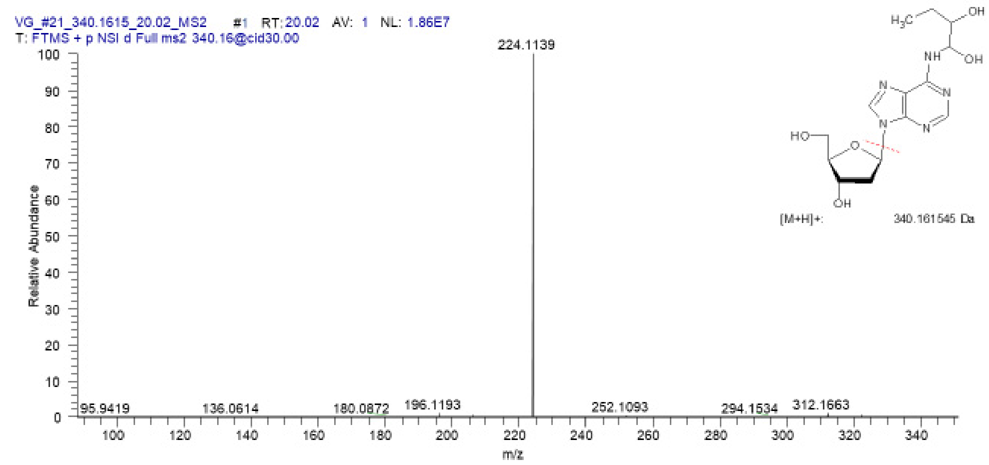
VG_#21_340.1615_20.02_MS2 #1 RT:20.02 AV: 1 NL: 1.86E7
T: FTMS + p NSI d Full ms2 340.16@cid30.00
224.1139
95.9419
136.0614
180.0872
196.1193
252.1093
294.1534
312.1663
Relative Abundance
m/z
[M+H]+: 340.161545 Da

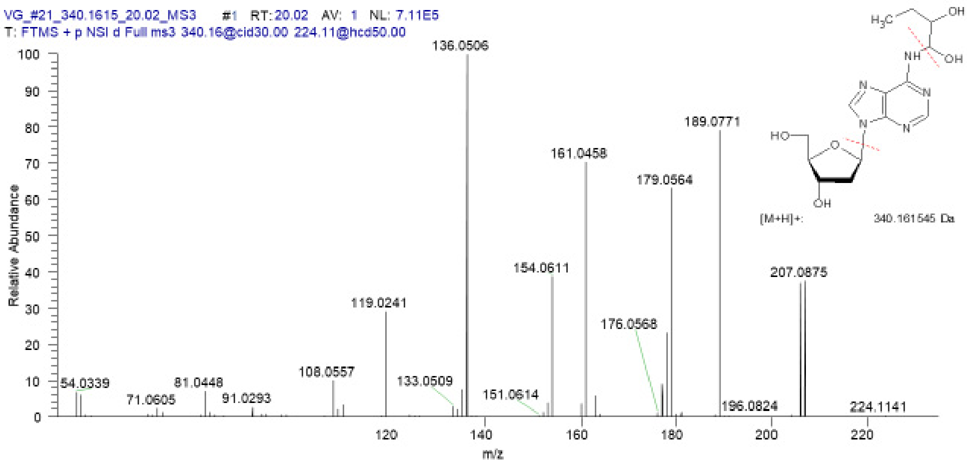
VG_#21_340.1615_20.02_MS3 #1 RT: 20.02 AV: 1 NL: 7.11E5
T: FTMS + p NSI d Full ms3 340.16@cid30.00 224.11@hcd50.00
Relative Abundance
m/z
136.0506
189.0771
161.0458
179.0564
154.0611
207.0875
119.0241
176.0568
108.0557
133.0509
151.0614
54.0339
81.0448
71.0605
91.0293
196.0824
224.1141
[M+H]+: 340.161545 Da

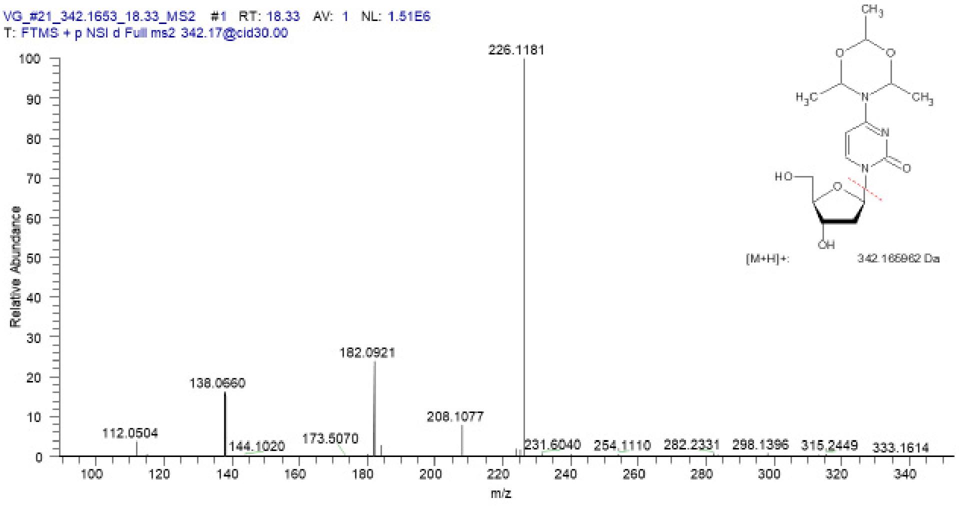
VG_#21_342.1653_18.33_MS2 #1 RT: 18.33 AV: 1 NL: 1.51E6
T: FTMS + p NSI d Full ms2 342.17@cid30.00
Relative Abundance
m/z
226.1181
182.0921
138.0660
208.1077
112.0504
144.1020
173.5070
231.6040
254.1110
282.2331
298.1396
315.2449
333.1614
[M+H]+: 342.165962 Da

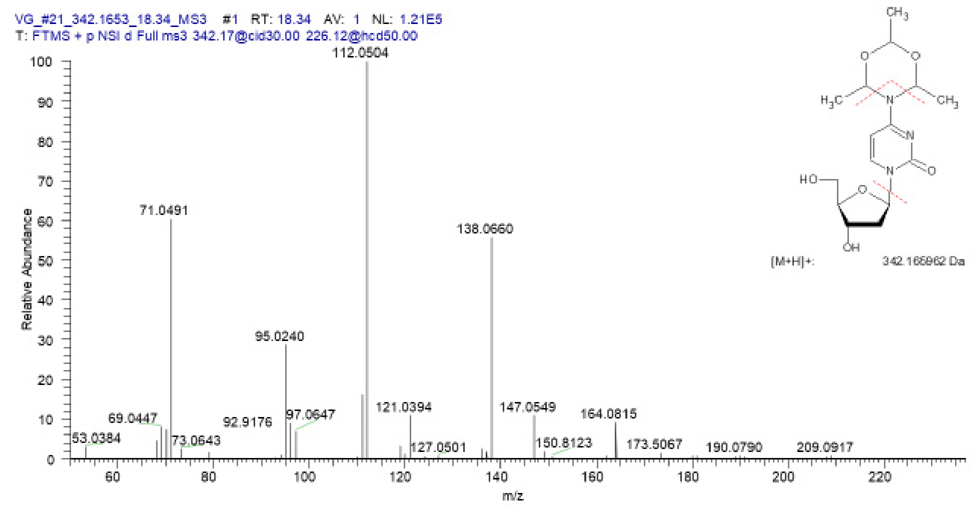
VG_#21_342.1653_18.34_MS3 #1 RT: 18.34 AV: 1 NL: 1.21E5
T: FTMS + p NSI d Full ms3 342.17@cid30.00 226.12@hcd50.00
112.0504
71.0491
138.0660
95.0240
69.0447
53.0384
73.0643
92.9176
97.0647
121.0394
127.0501
147.0549
150.8123
164.0815
173.5067
190.0790
209.0917
Relative Abundance
m/z
[M+H]+: 342.165962 Da

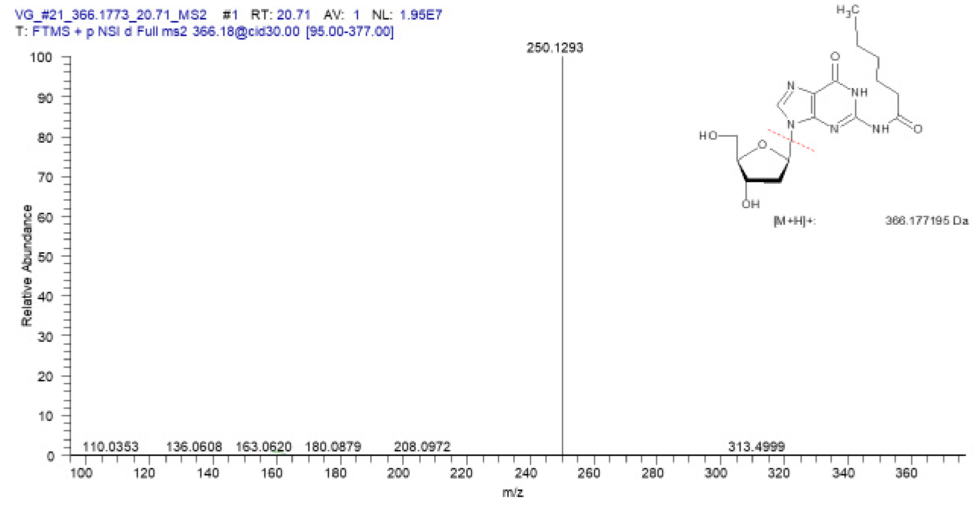
VG_#21_366.1773_20.71_MS2 #1 RT: 20.71 AV: 1 NL: 1.95E7
T: FTMS + p NSI d Full ms2 366.18@cid30.00 [95.00-377.00]
250.1293
110.0353
136.0608
163.0620
180.0879
208.0972
313.4999
Relative Abundance
m/z
[M+H]+: 366.177195 Da

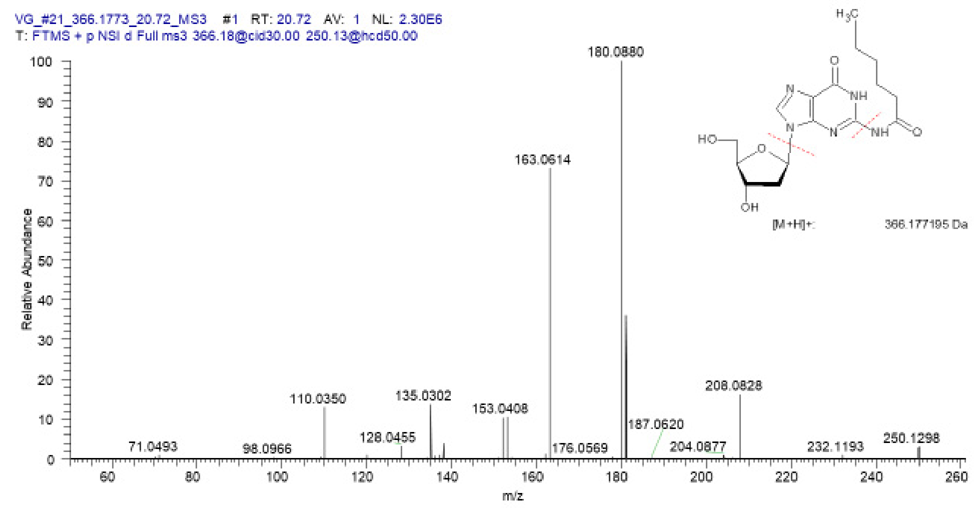
VG_#21_366.1773_20.72_MS3 #1 RT: 20.72 AV: 1 NL: 2.30E6
T: FTMS + p NSI d Full ms3 366.18@cid30.00 250.13@hcd50.00
Relative Abundance
m/z
180.0880
163.0614
208.0828
110.0350
135.0302
153.0408
128.0455
187.0620
71.0493
98.0966
176.0569
204.0877
232.1193
250.1298
[M+H]+: 366.177195 Da

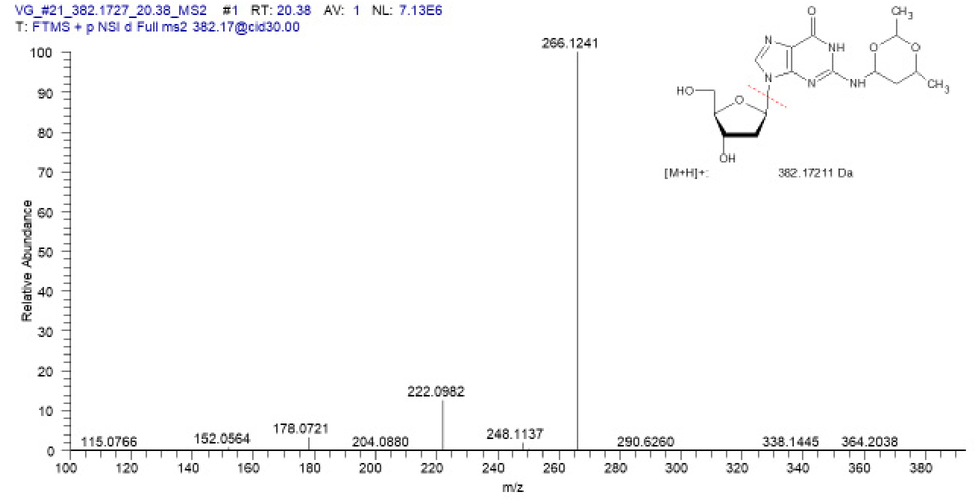
VG_#21_382.1727_20.38_MS2 #1 RT: 20.38 AV: 1 NL: 7.13E6
T: FTMS + p NSI d Full ms2 382.17@cid30.00
Relative Abundance
m/z
266.1241
222.0982
178.0721
248.1137
115.0766
152.0564
204.0880
290.6260
338.1445
364.2038
[M+H]+: 382.17211 Da

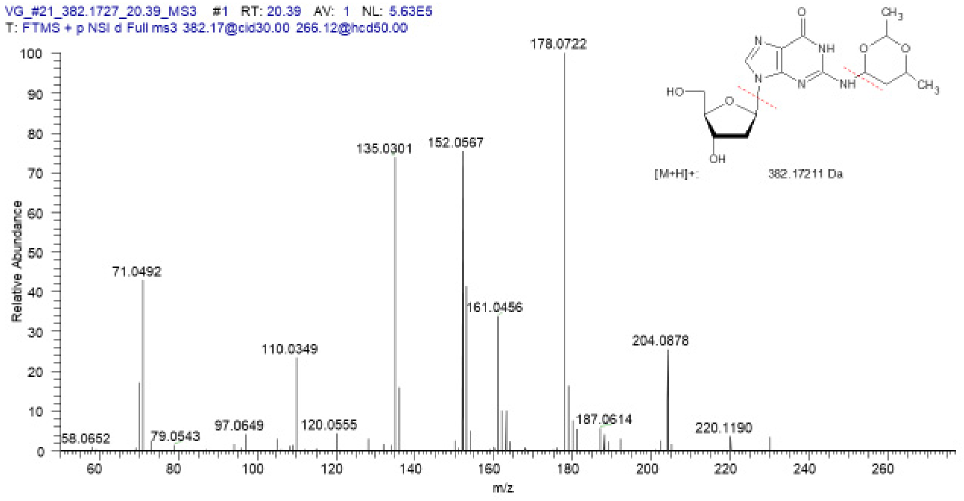
VG_#21_382.1727_20.39_MS3 #1 RT: 20.39 AV: 1 NL: 5.63E5
T: FTMS + p NSI d Full ms3 382.17@cid30.00 266.12@hcd50.00
Relative Abundance
m/z
178.0722
152.0567
135.0301
71.0492
161.0456
110.0349
204.0878
187.0614
220.1190
120.0555
97.0649
79.0543
58.0652
[M+H]+:
382.17211 Da

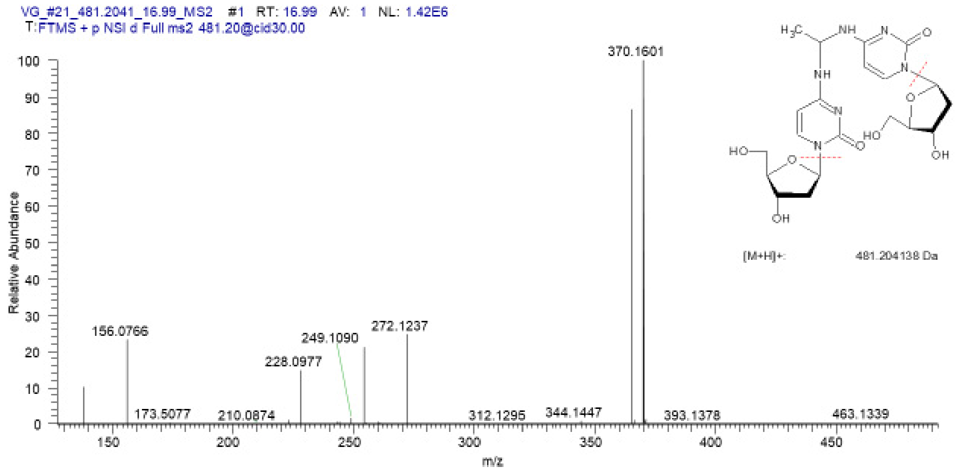
VG_#21_481.2041_16.99_MS2 #1 RT: 16.99 AV: 1 NL: 1.42E6
T:FTMS + p NSI d Full ms2 481.20@cid30.00
Relative Abundance
m/z
370.1601
156.0766
249.1090
272.1237
228.0977
173.5077
210.0874
312.1295
344.1447
393.1378
463.1339
[M+H]+:
481.204138 Da

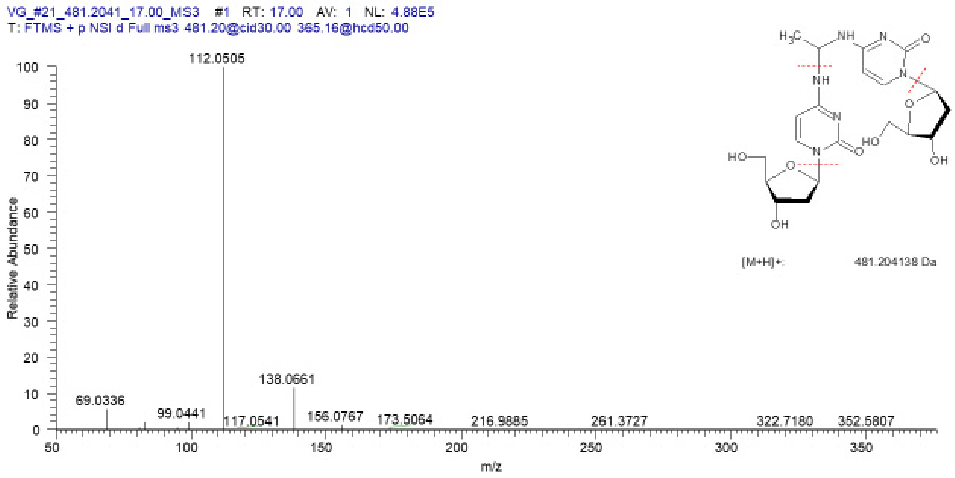
VG_#21_481.2041_17.00_MS3 #1 RT: 17.00 AV: 1 NL: 4.88E5
T: FTMS + p NSI d Full ms3 481.20@cid30.00 365.16@hcd50.00
Relative Abundance
112.0505
138.0661
69.0336
99.0441
117.0541
156.0767
173.5064
216.9885
261.3727
322.7180
352.5807
m/z
[M+H]+:
481.204138 Da

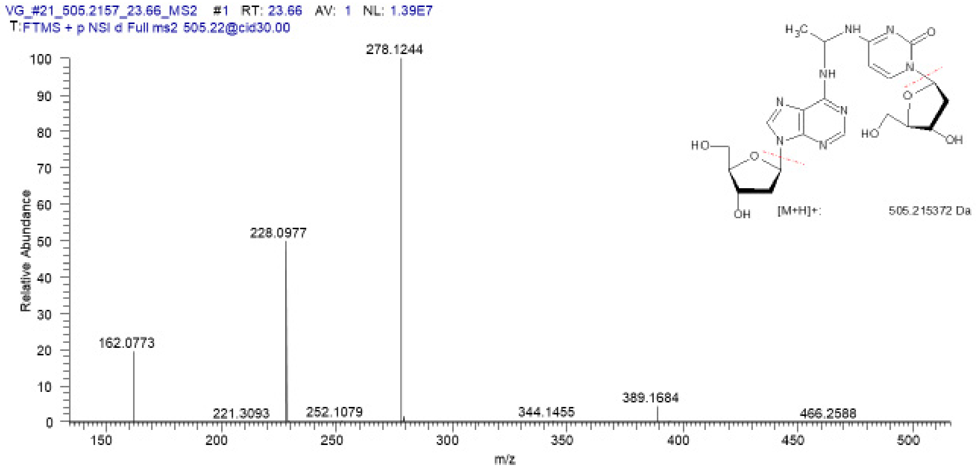
VG_#21_505.2157_23.66_MS2 #1 RT: 23.66 AV: 1 NL: 1.39E7
T:FTMS + p NSI d Full ms2 505.22@cid30.00
Relative Abundance
278.1244
228.0977
162.0773
221.3093
252.1079
344.1455
389.1684
466.2588
m/z
[M+H]+:
505.215372 Da

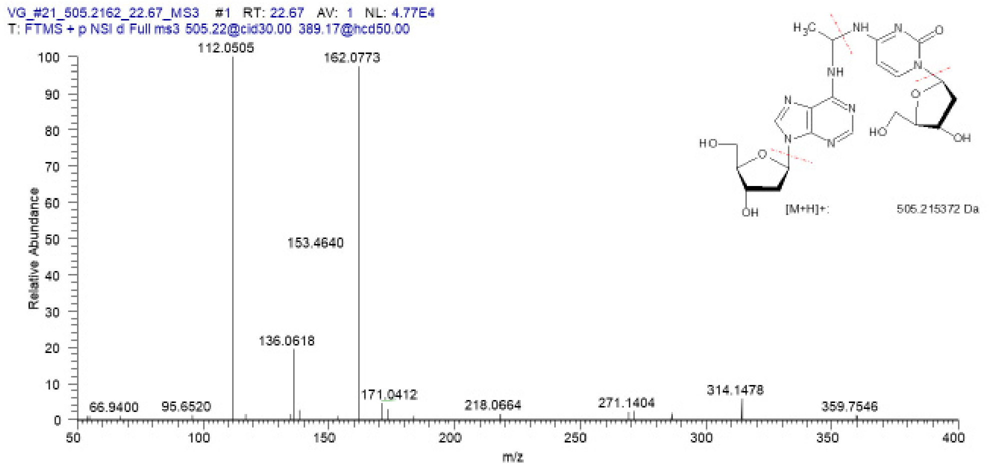
VG_#21_505.2162_22.67_MS3 #1 RT: 22.67 AV: 1 NL: 4.77E4
T: FTMS + p NSI d Full ms3 505.22@cid30.00 389.17@hcd50.00
112.0505
162.0773
153.4640
136.0618
171.0412
218.0664
271.1404
314.1478
359.7546
66.9400
95.6520
Relative Abundance
m/z
[M+H]+:
505.215372 Da

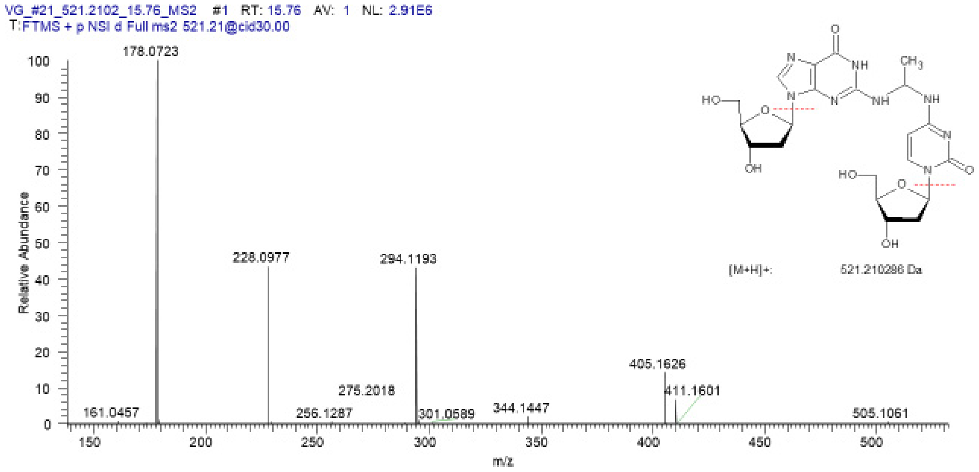
VG_#21_521.2102_15.76_MS2 #1 RT: 15.76 AV: 1 NL: 2.91E6
T: FTMS + p NSI d Full ms2 521.21@cid30.00
178.0723
228.0977
294.1193
405.1626
275.2018
411.1601
161.0457
256.1287
301.0589
344.1447
505.1061
Relative Abundance
m/z
[M+H]+:
521.210286 Da

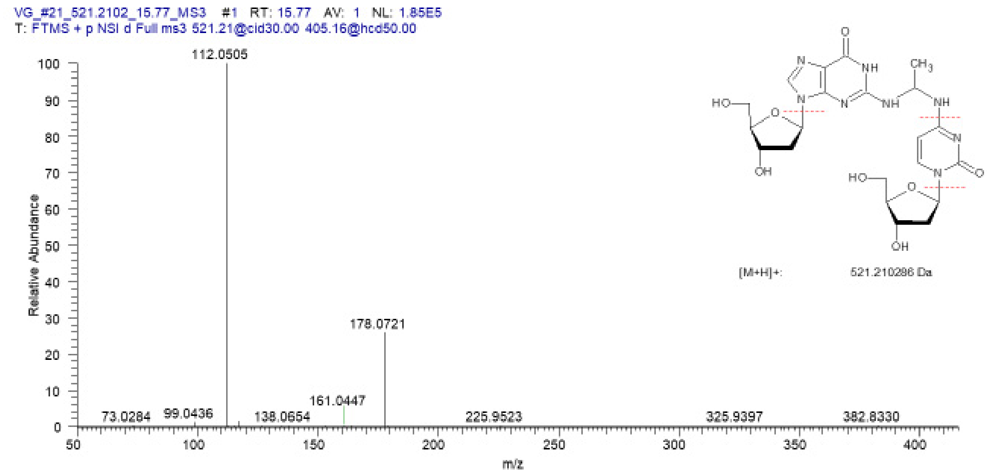

VG_#21_521.2102_15.77_MS3 #1 RT: 15.77 AV: 1 NL: 1.85E5
T: FTMS + p NSI d Full ms3 521.21@cid30.00 405.16@hcd50.00
112.0505
178.0721
161.0447
73.0284
99.0436
138.0654
225.9523
325.9397
382.8330
Relative Abundance
m/z
[M+H]+:
521.210286 Da

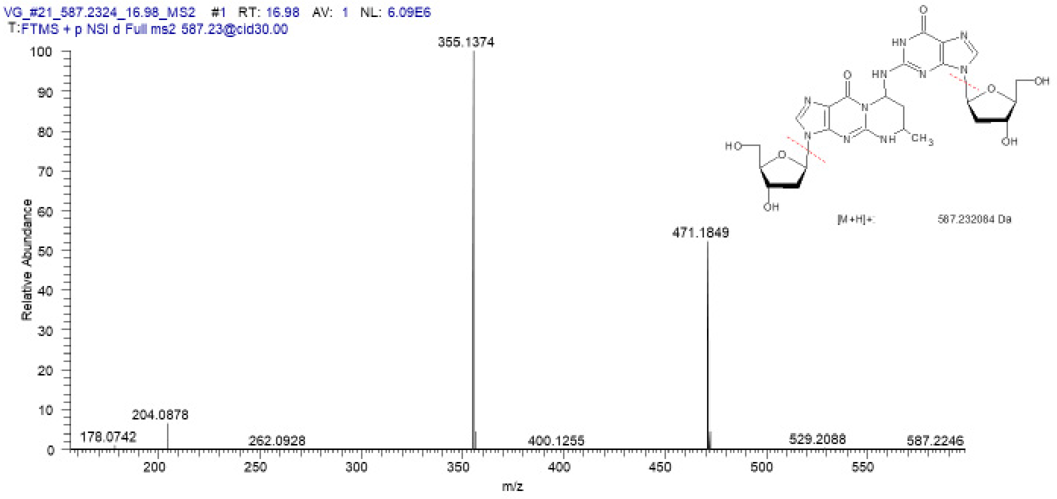

VG_#21_587.2324_16.98_MS2 #1 RT: 16.98 AV: 1 NL: 6.09E6
T:FTMS + p NSI d Full ms2 587.23@cid30.00
355.1374
471.1849
204.0878
178.0742
262.0928
400.1255
529.2088
587.2246
Relative Abundance
m/z
[M+H]+:
587.232084 Da

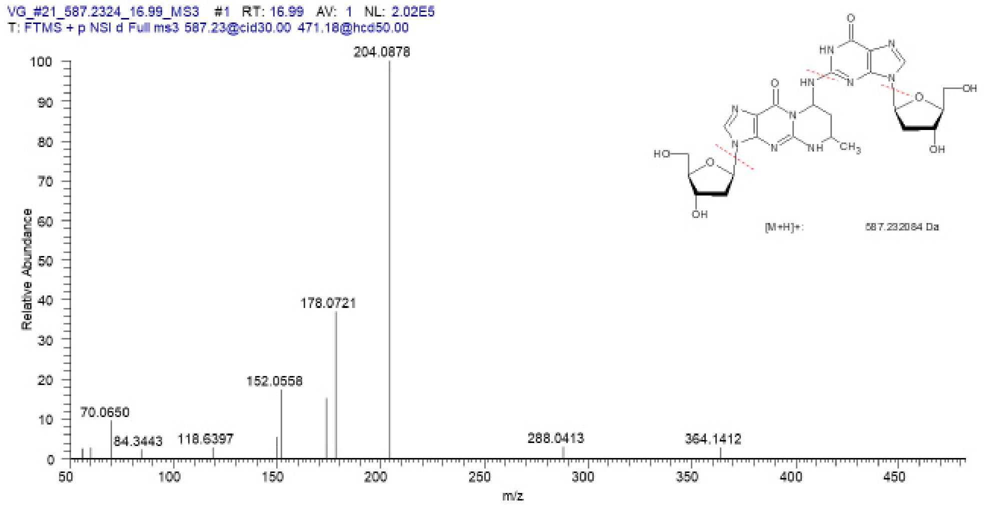
VG_#21_587.2324_16.99_MS3 #1 RT: 16.99 AV: 1 NL: 2.02E5
T: FTMS + p NSI d Full ms3 587.23@cid30.00 471.18@hcd50.00
204.0878
178.0721
152.0558
70.0650
84.3443
118.6397
288.0413
364.1412
Relative Abundance
m/z
[M+H]+: 587.232084 Da

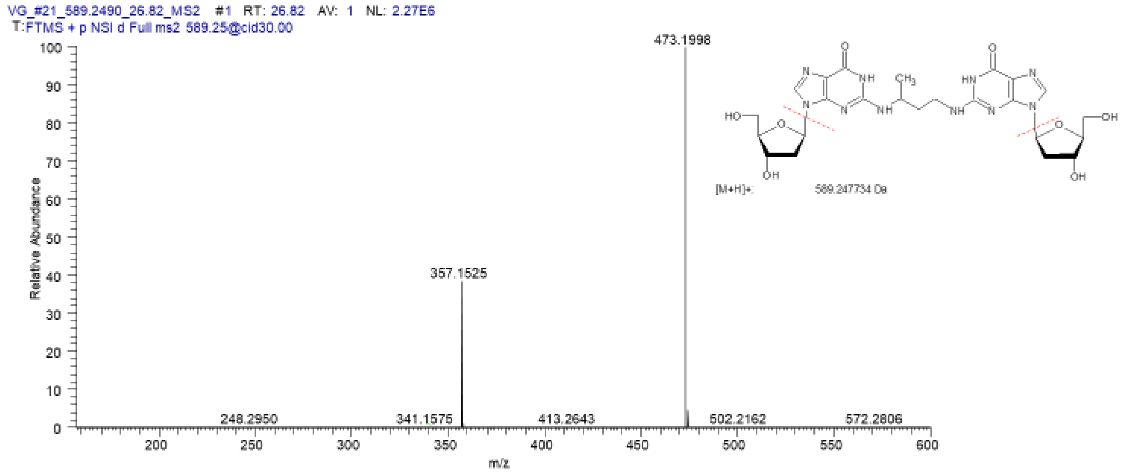
VG_#21_589.2490_26.82_MS2 #1 RT: 26.82 AV: 1 NL: 2.27E6
T:FTMS + p NSI d Full ms2 589.25@cid30.00
473.1998
357.1525
248.2950
341.1575
413.2643
502.2162
572.2806
Relative Abundance
m/z
[M+H]+: 589.247734 Da

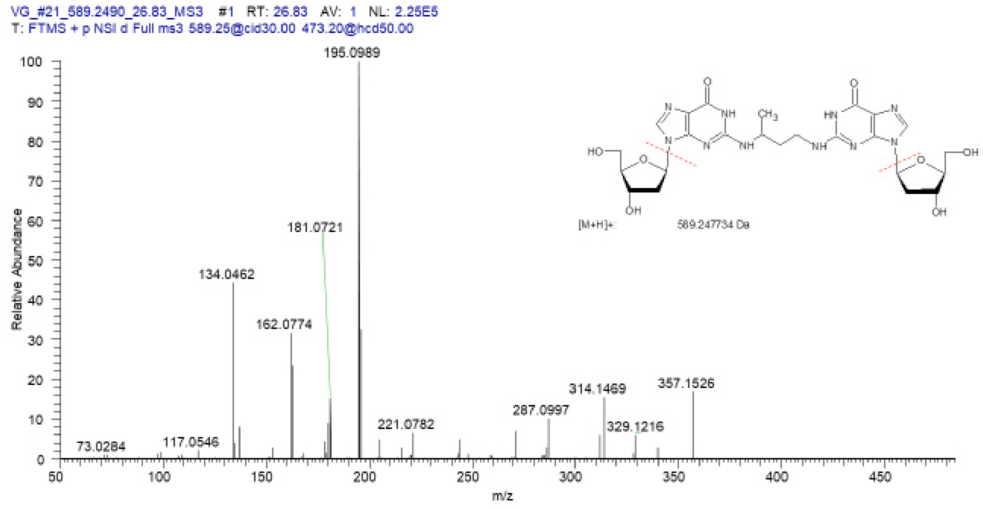

Concentration-dependent DNA adduct generation

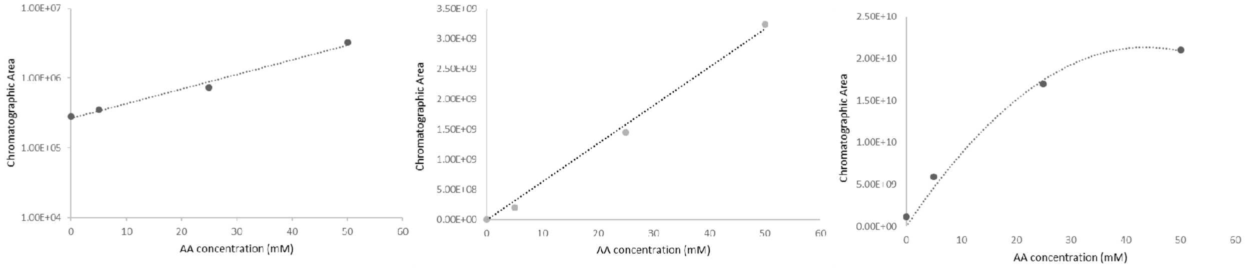

Figure S3. Concentration-dependent formation of adducts (from left to right *N*[4]-ethyl-dC, *N*[6]-ethyl-dA and *N*[2]-ethyl-dG) in the reaction of acetaldehyde with CT-DNA. Data are based on area of each adduct peak as determined by DDA-CNL/MS[3].

NMR characterization of N^6-ethyl-dA, [D$_5$]N^6-ethyl-dA, N^4-ethyl-dC and [D$_5$]N^4-ethyl-dC

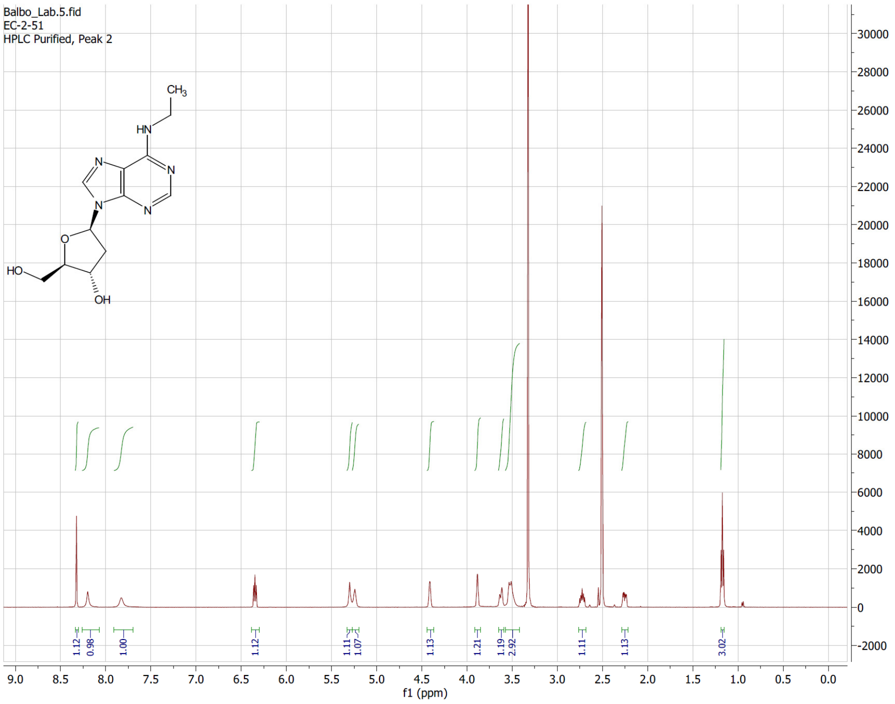

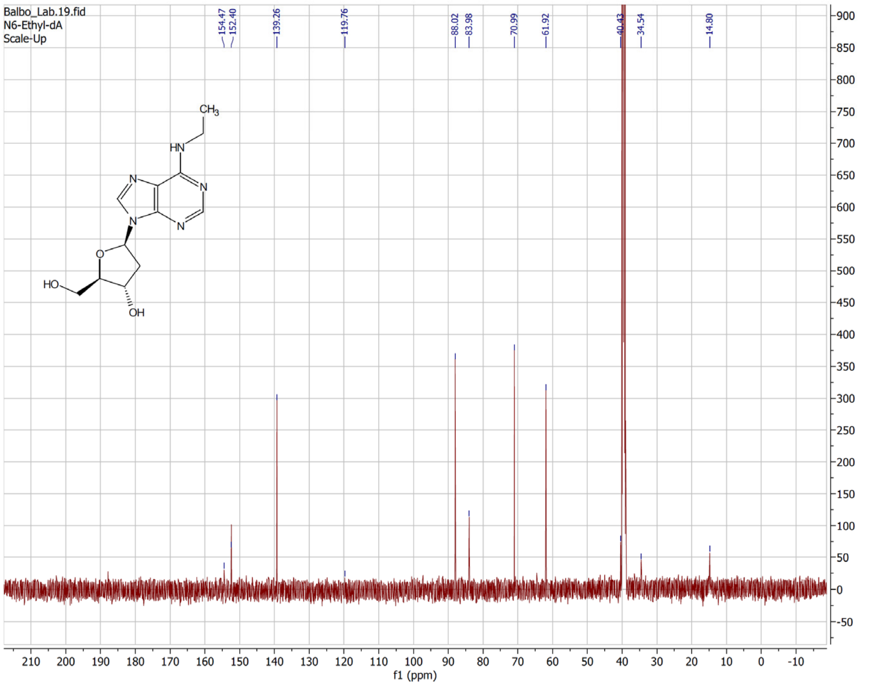

Figure S4. ^{1}H (top) and ^{13}C (bottom) NMR spectra of N^6-ethyl-dA.

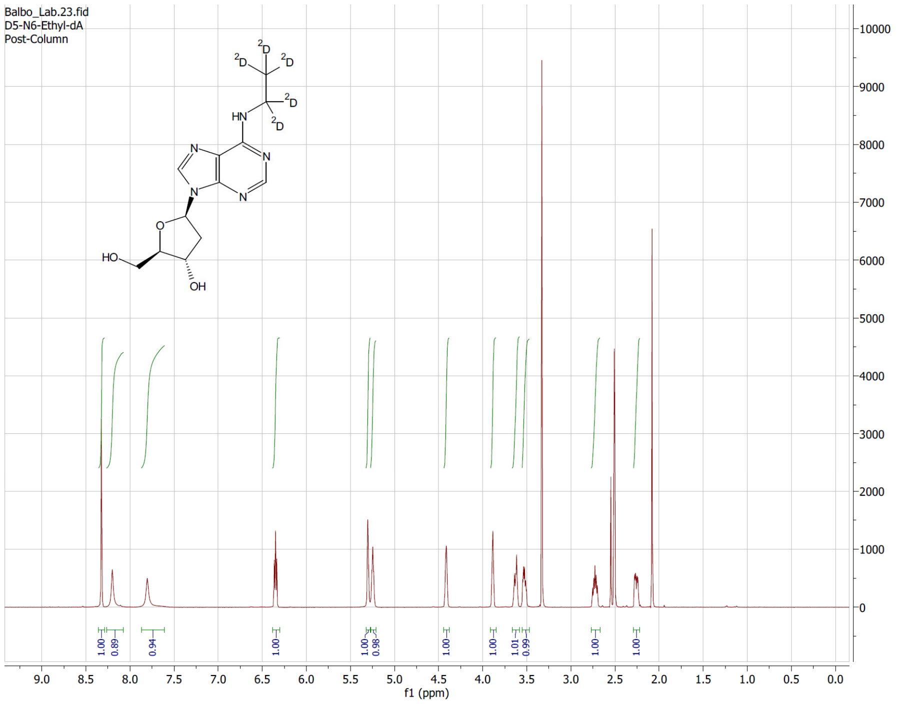

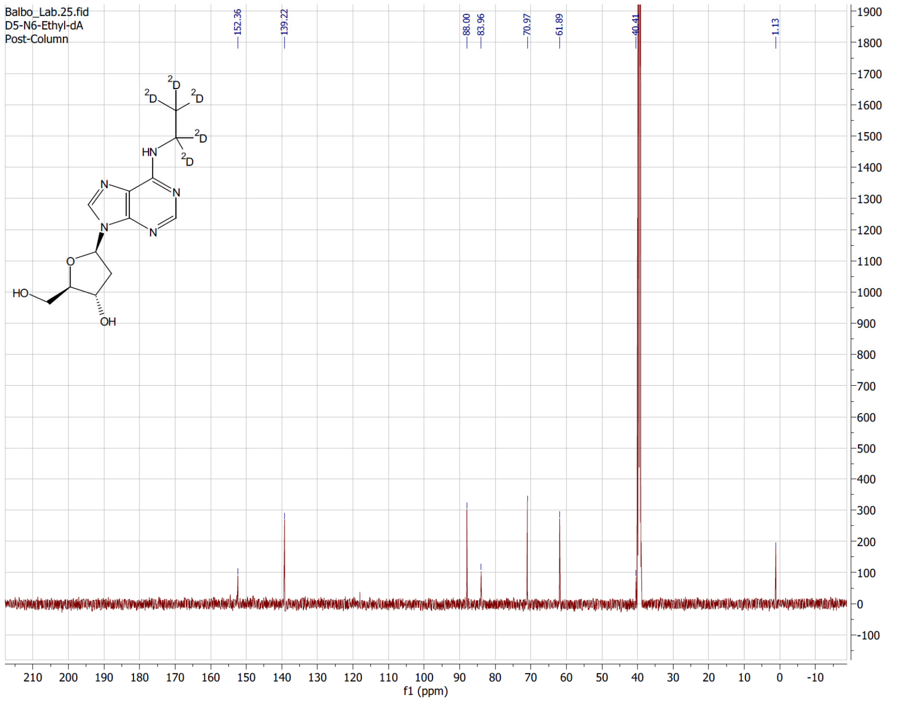

Figure S5. ^{1}H (top) and ^{13}C (bottom) NMR spectra of [D$_5$]*N*^{6}-ethyl-dA.

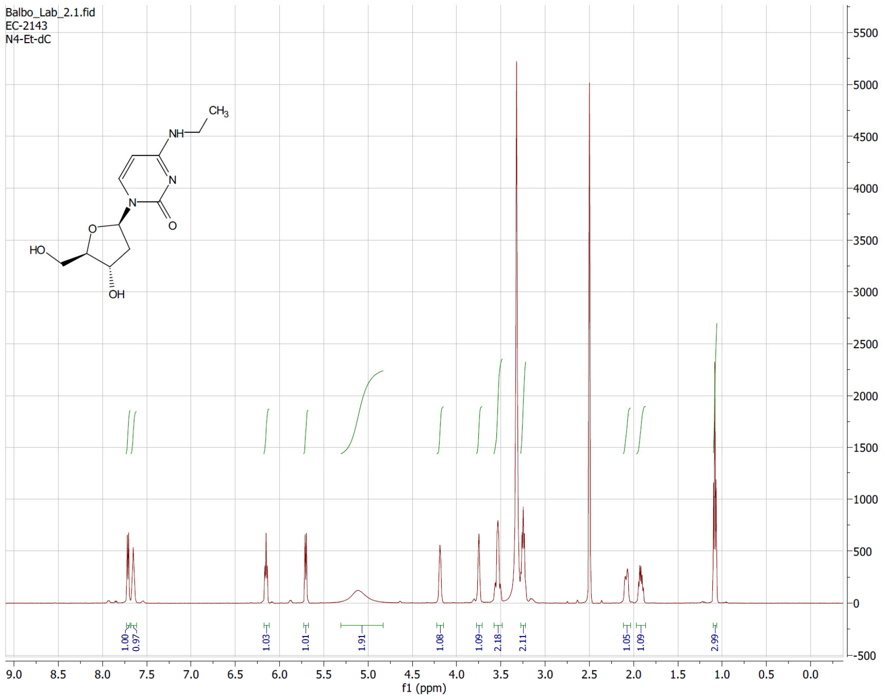

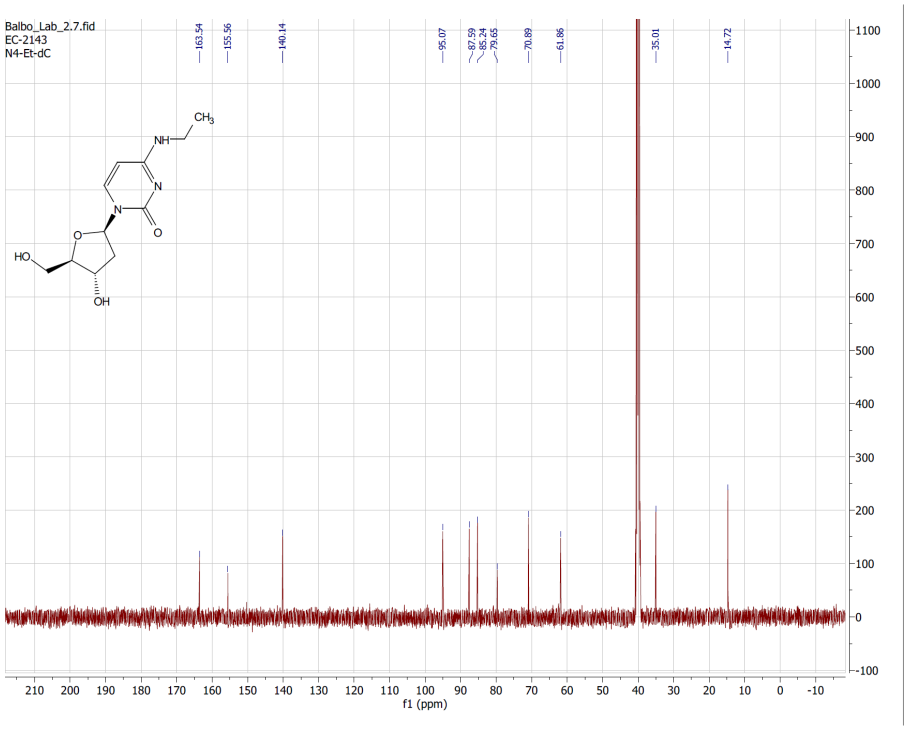

Figure S6. ^{1}H (top) and ^{13}C (bottom) NMR spectra of N^4-ethyl-dC.

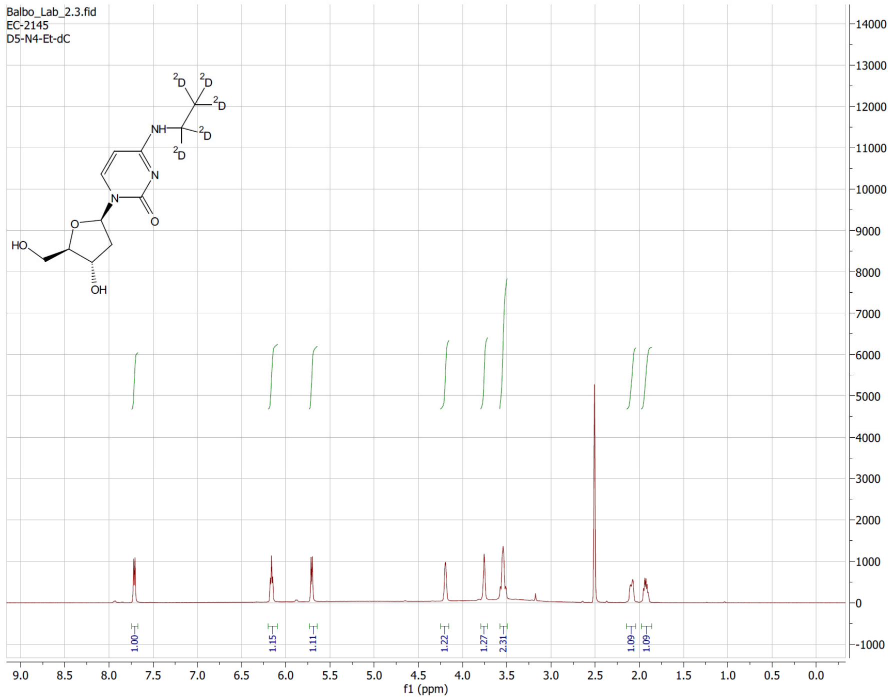

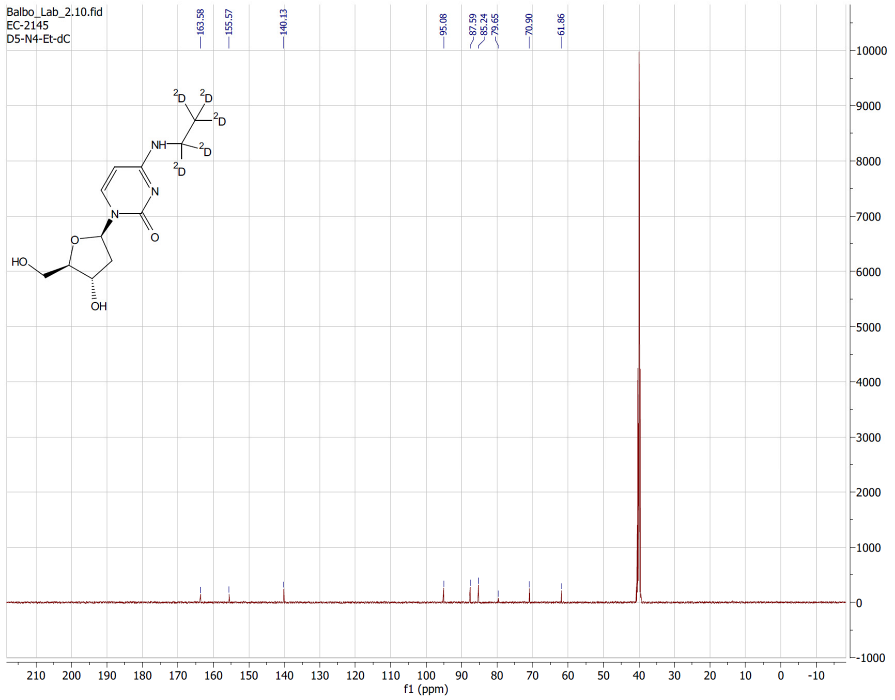

Figure S7. ^{1}H (top) and ^{13}C (bottom) NMR spectra of [D_5]N^4-ethyl-dC.

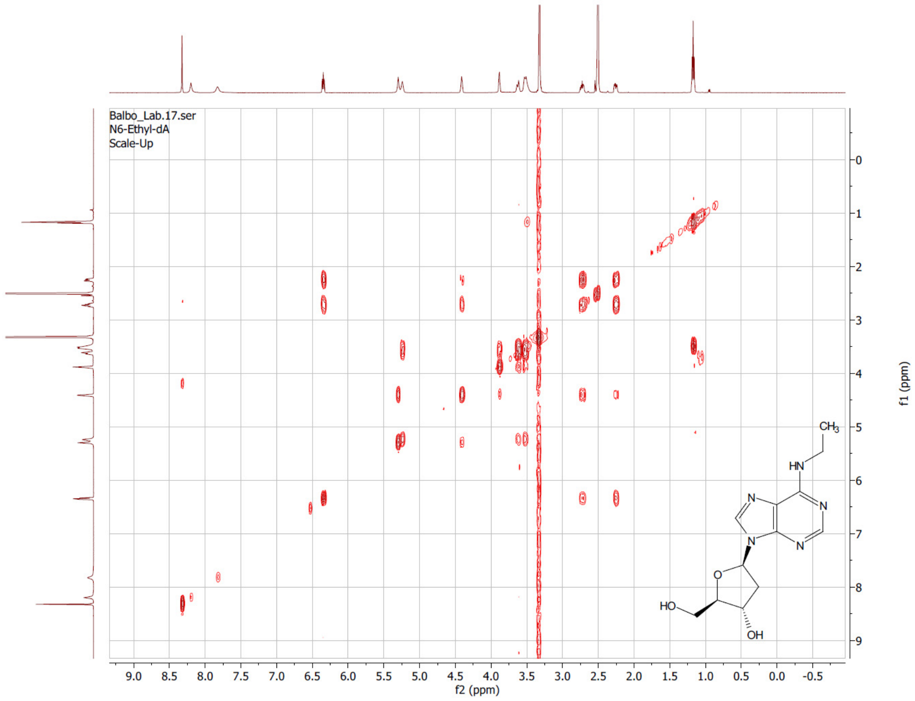

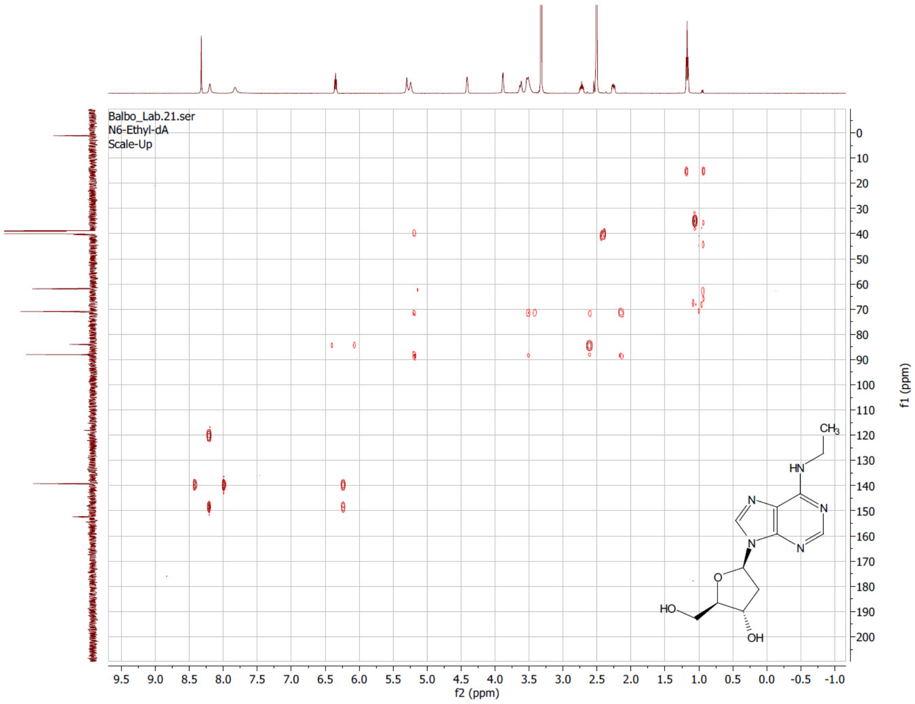

Figure S8. COSY (top) and HSQC (bottom) NMR spectra of N^6-ethyl-dA.

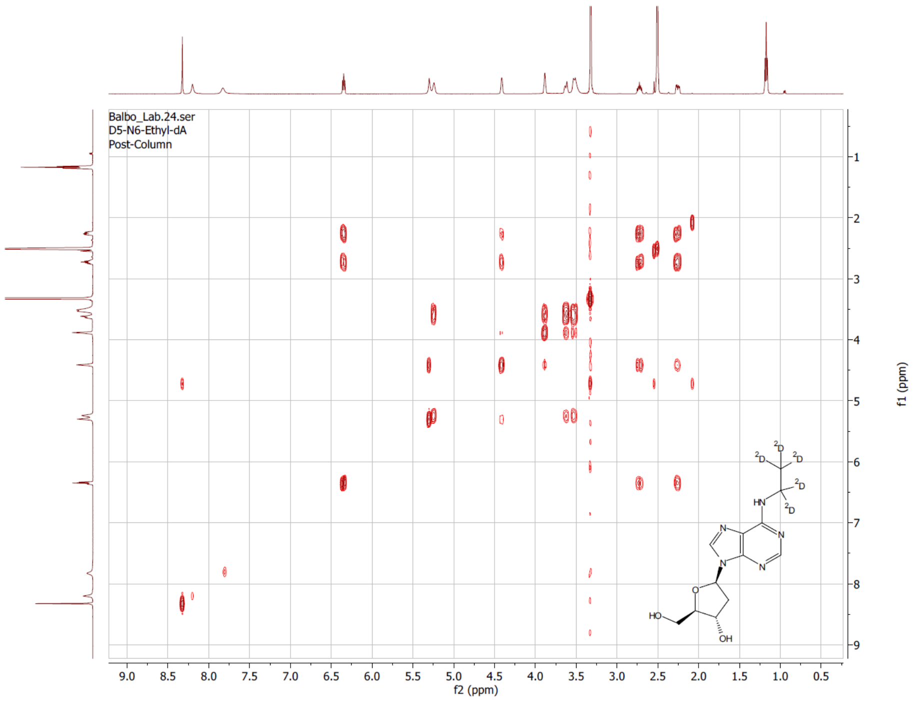

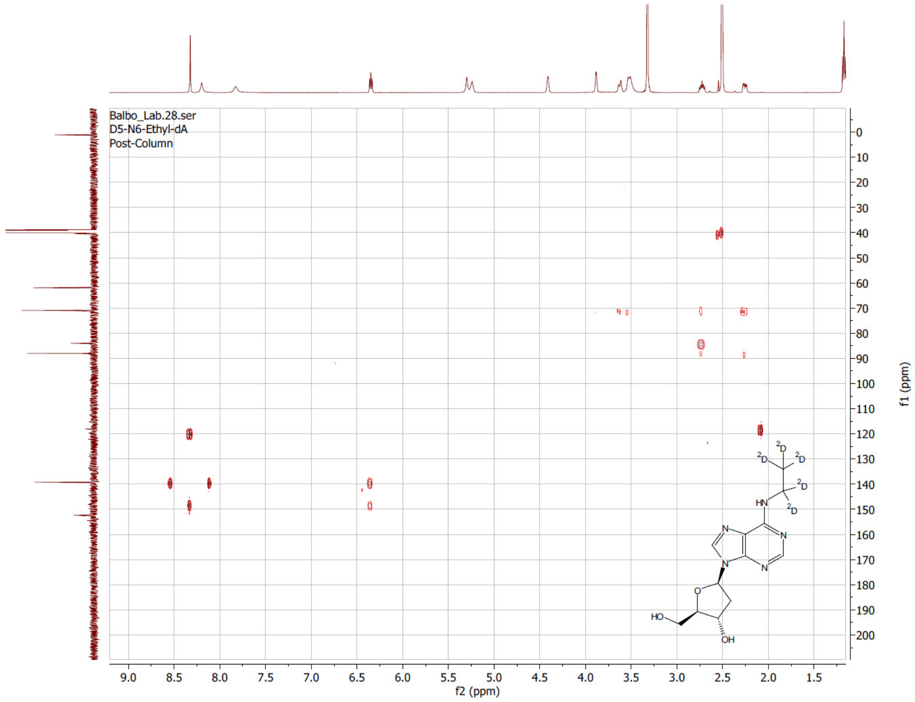

Figure S9. COSY (top) and HSQC (bottom) NMR spectra of [D5]N^6-ethyl-dA.

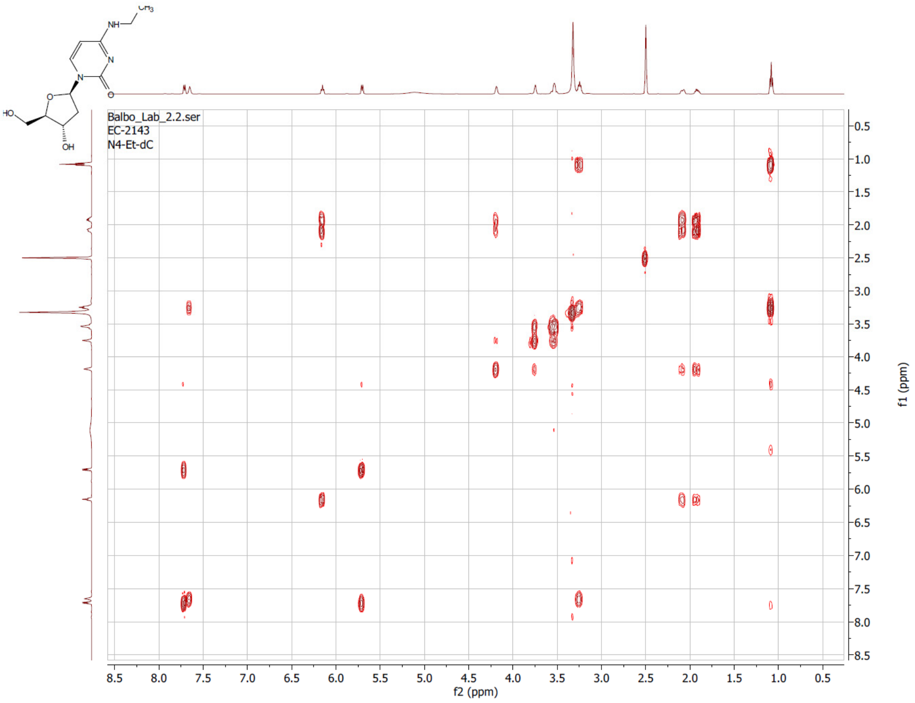

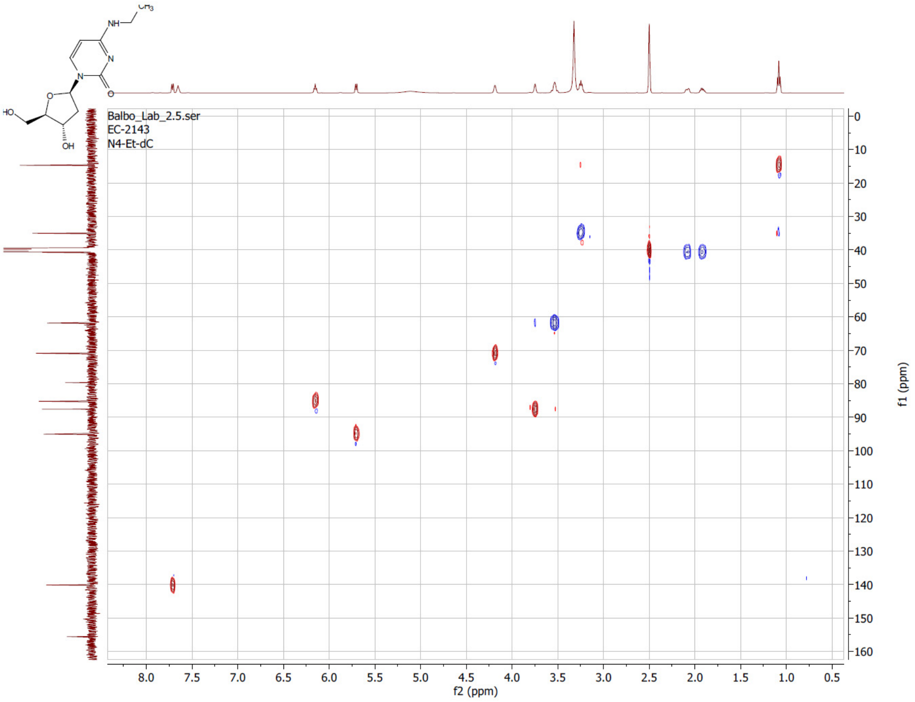

Figure S10. COSY (top) and HSQC (bottom) NMR spectra of *N*[4]-ethyl-dC.

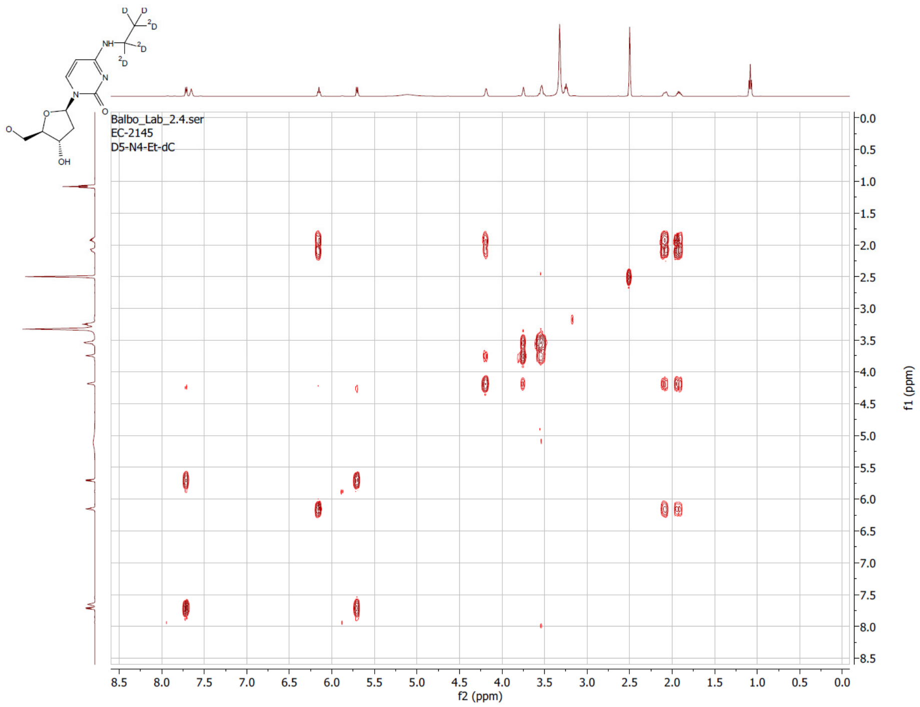

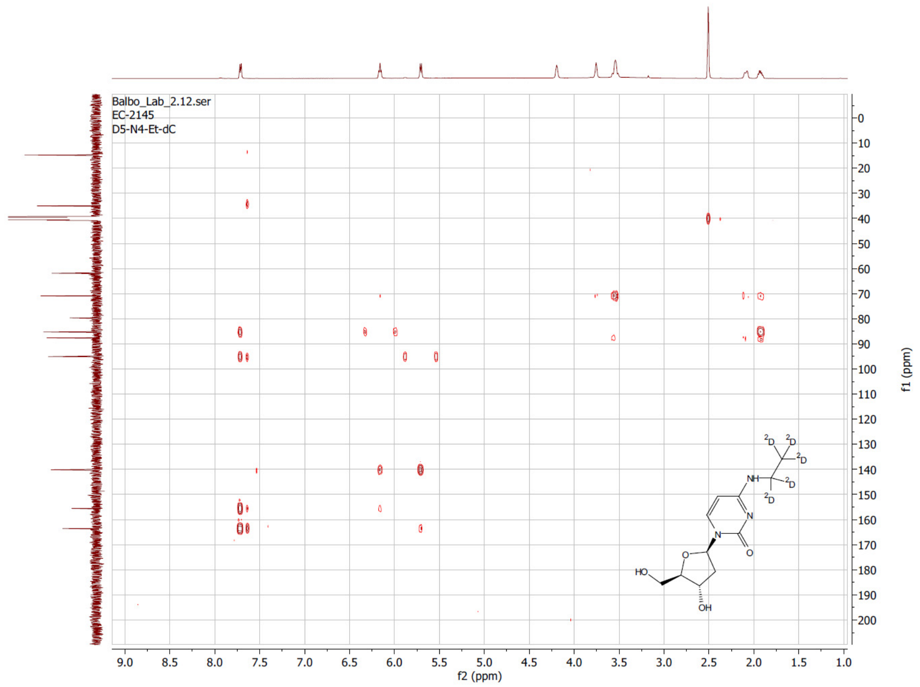

Figure S11. COSY (top) and HSQC (bottom) NMR spectra of [D$_5$]*N*^{4}-ethyl-dC.

Table S1. *N*[4]-ethyl-dC, [D$_5$]*N*[4]-ethyl-dC, *N*[6]-ethyl-dA, [D$_5$]*N*[6]-ethyl-dA proton and carbon NMR signals in DMSO-d$_6$.

**Compound**	**^{1}H-NMR (500 MHz; DMSO-d$_6$)**	**^{13}C-NMR (126 MHz; DMSO)**
N[4]-ethyl-dC	δ 7.71 (d, J = 7.3 Hz, 1H), 7.65 (s, 1H), 6.16 (d, J = 6.8 Hz, 1H), 5.71 (d, J = 7.5 Hz, 1H), 5.20-5.03 (m, 2H), 4.19-4.19 (m, 1H), 3.75 (s, 1H), 3.55-3.53 (m, 2H), 3.25 (t, J = 6.8 Hz, 2H), 2.10-2.06 (m, 1H), 1.94-1.90 (m, 1H), 1.08 (t, J = 6.8 Hz, 3H).	δ 163.1, 155.1, 139.7, 94.6, 87.1, 84.8, 70.4, 61.4, 40.3, 34.5, 14.3
[D$_5$]*N*[4]-ethyl-dC	δ 7.71 (d, J = 7.4 Hz, 1H), 7.63 (s, 1H), 6.17-6.14 (m, 1H), 5.70 (d, J = 7.5 Hz, 1H), 5.17-4.97 (m, 2H), 4.19-4.18 (m, 1H), 3.75 (s, 1H), 3.57-3.50 (m, 2H), 2.10-2.06 (m, 1H), 1.94-1.89 (m, 1H)	δ 163.1, 155.1, 139.6, 94.6, 87.1, 84.8, 70.4, 61.4, 40.3, 34.1, 13.2
N[6]-ethyl-dA	δ 8.31 (s, 1H), 8.19 (s, 1H), 7.82 (s, 1H), 6.34 (t, J = 6.9 Hz, 1H), 5.27 (d, J = 22.0 Hz, 2H), 4.40 (t, J = 2.5 Hz, 1H), 3.87 (t, J = 3.2 Hz, 1H), 3.57 (m, J = 3.7 Hz, 4H), 2.74-2.69 (m, 1H), 2.27-2.23 (m, 1H), 1.16 (t, J = 7.1 Hz, 3H)	δ 154.9, 152.8, 148.8, 139.7, 120.4, 88.5, 84.4, 71.4, 62.4, 40.1, 35.0, 15.2.

[D5]*N*6-ethyl-dA	δ 8.32 (s, 1H), 8.19 (t, J = 0.3 Hz, 1H), 7.79 (t, J = 0.5 Hz, 1H), 6.34 (t, J = 7.0 Hz, 1H), 5.30-5.29 (m, 1H), 5.25-5.23 (m, 1H), 4.41 (dt, J = 1.1, 0.5 Hz, 1H), 3.88 (d, J = 2.4 Hz, 1H), 3.64-3.60 (m, 1H), 3.54-3.50 (m, 1H), 2.75-2.69 (m, 1H), 2.25 (ddd, J = 13.1, 5.9, 2.5 Hz, 1H)	δ 154.9, 152.8, 148.5, 120. 3, 139.7, 88.5, 84.4, 71.5, 62.4, 40.9

Table S2. 3′,5′-*bis*-*O*-acetyl-2′-deoxyuridine, 4-chloro-1-*N*-(3',5'-*bis*-*O*-acetyl-2'-deoxyribosyl)-2-pyrimidinone proton and carbon NMR signals in $CDCl_3$.

**Compound**	**^{1}H-NMR (500 MHz; $CDCl_3$)**	**^{13}C-NMR (126 MHz; $CDCl_3$)**
3′, 5′-*bis*-*O*-acetyl-2′-deoxyuridine	δ 9.48 (s, 1H), 7.48 (d, J = 8.2 Hz, 1H), 6.27 (dd, J = 8.0, 5.9 Hz, 1H), 5.77 (d, J = 8.1 Hz, 1H), 5.20 (d, J = 6.5 Hz, 1H), 4.31 (m, J = 3.4 Hz, 3H), 2.52 (dd, J = 14.2, 5.6 Hz, 1H), 2.15 (dt, J = 14.5, 7.3 Hz, 1H), 2.12 (s, 6H).	δ 170.28, 163.53, 150.51, 138.99, 103.0, 85.3, 82.3, 74.1, 63.8, 37.7, 20.88, 20.78
4-chloro-1-*N*-(3', 5'-*bis*-*O*-acetyl-2'-deoxyribosyl)-2-pyrimidinone	δ 8.00 (d, J = 7.1 Hz, 1H), 6.43 (d, J = 7.1 Hz, 1H), 6.15 (dd, J = 7.4, 5.9 Hz, 1H), 5.21 (dd, J = 4.2, 2.2 Hz, 1H), 4.39-4.36 (m, 3H), 2.91 (ddd, J = 14.5, 5.6, 2.1 Hz, 1H), 2.11 (s, 3H), 2.07 (q, J = 6.7 Hz, 4H)	δ 170.33, 170.15, 167.0, 153.0, 142.7, 105.2, 88.1, 83.5, 74.0, 63.6, 39.1, 20.86, 20.78

HRMS characterization of *N*6-ethyl-dA, [D5]*N*6-ethyl-dA, *N*4-ethyl-dC and [D5]*N*4-ethyl-dC

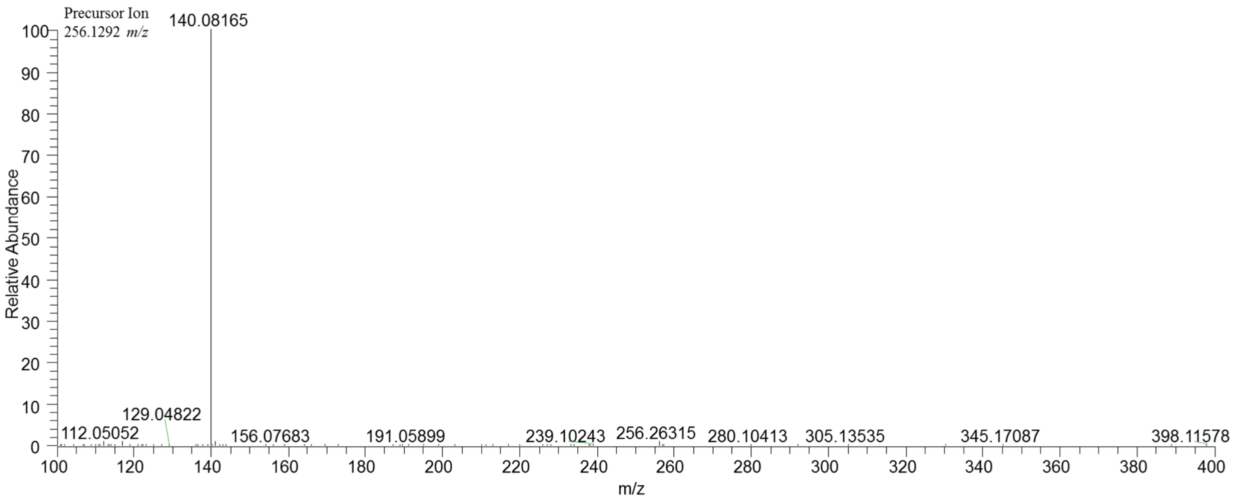

Figure S12. MS^2 spectrum of N^4-ethyl-dC (256.1292 *m*/*z*).

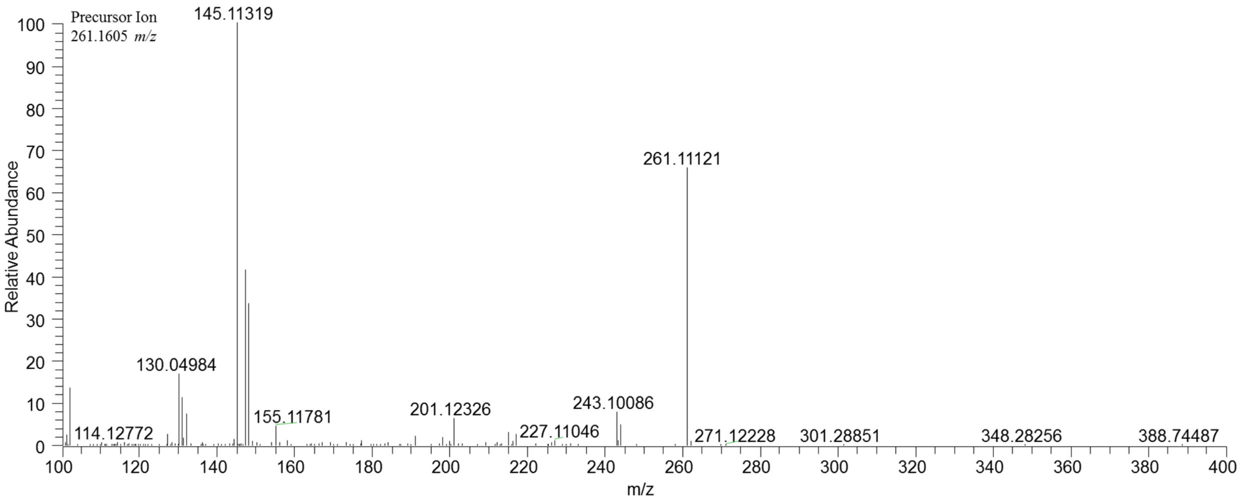

Figure S13. MS^2 spectrum of [D_5]N^4-ethyl-dC (261.1605 *m*/*z*).

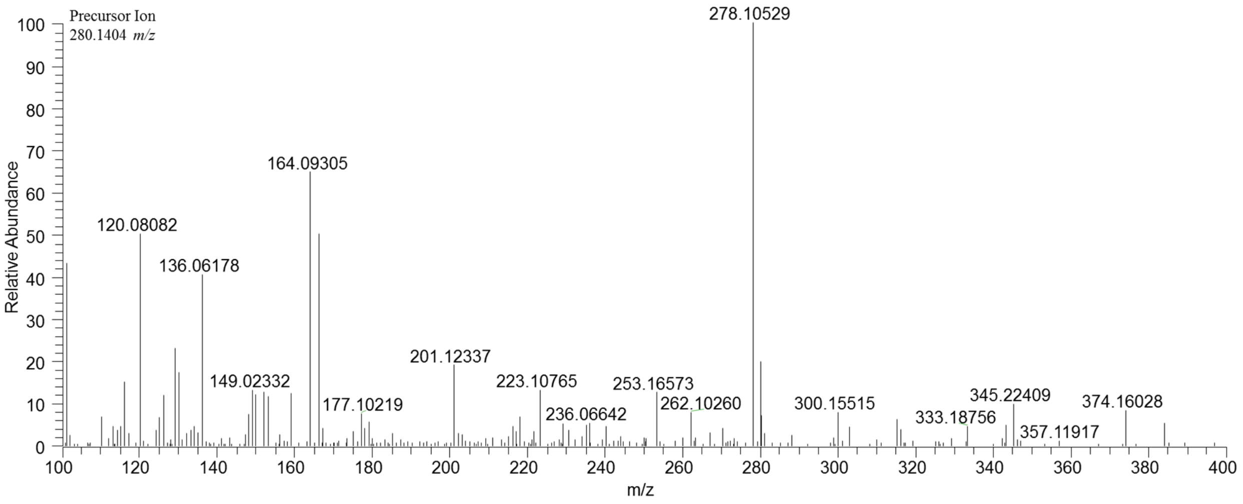

Figure S14. MS^2 spectrum of N^6-ethyl-dA (280.1404 *m/z*).

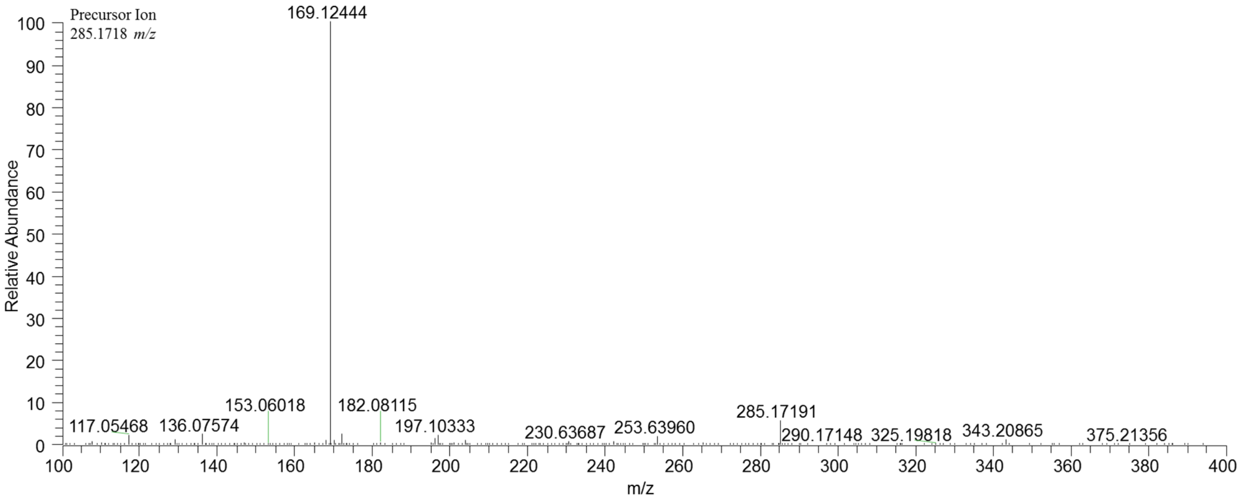

Figure S15. MS^2 spectrum of $[D_5]N^6$-ethyl-dA (285.1718 *m/z*).

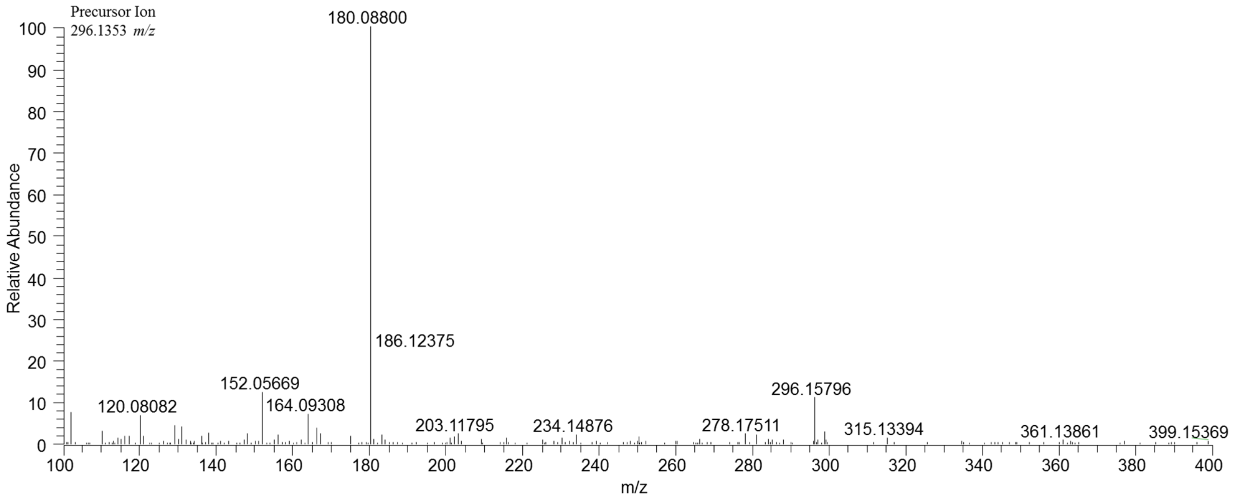

Figure S16. MS^2 spectrum of N^2-ethyl-dG (296.1353 *m/z*)

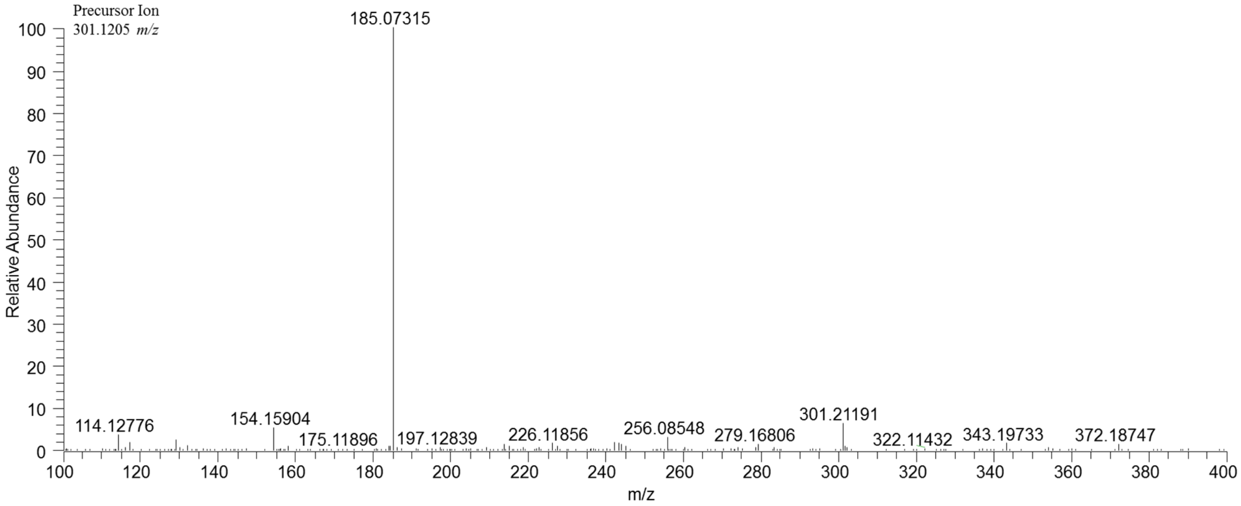

Figure S17. MS^2 spectrum of $[^{15}N_5]N^2$-ethyl-dG (301.1205 *m/z*).

Quantitation of N^4-ethyl-dC, N^6-ethyl-dA and N^2-ethyl-dG in human oral cells

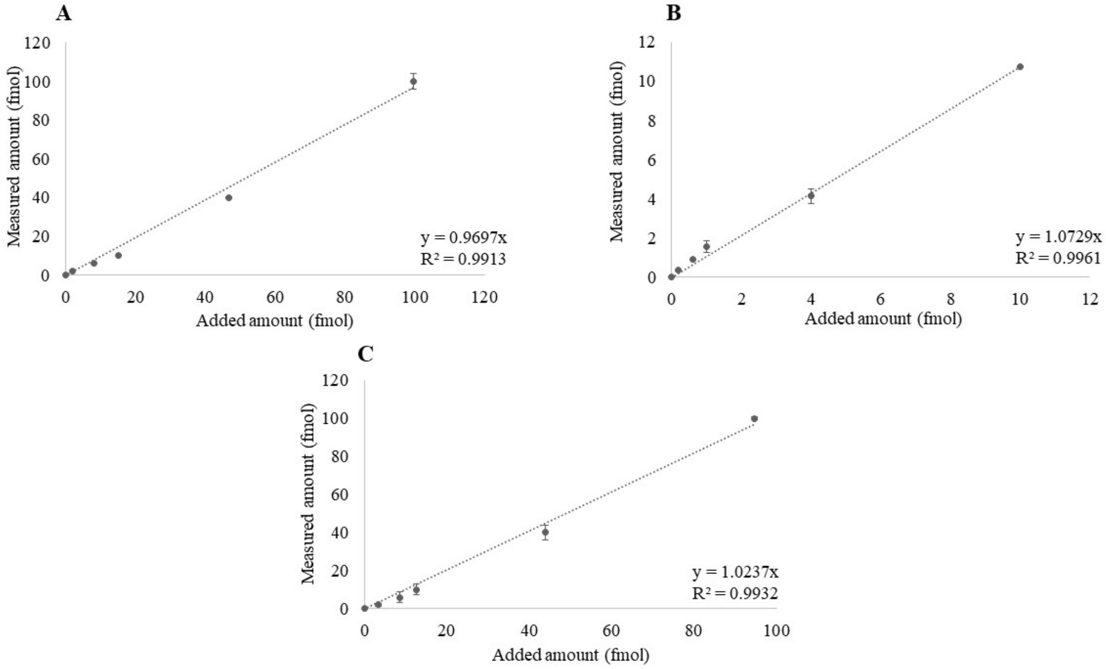

Figure S18. Relationship between added amount and measured amount in CT-DNA of A: N^2-ethyl-dG, B: N^6-ethyl-dA and C: N^4-ethyl-dC.

Table S3. Peak area of ethyl-adducts in alcohol-exposed and non-exposed oral cells.

	**Alcohol**	**Alcohol**	**Alcohol**	**CTRL**	**CTRL**	**CTRL**
**N^4-ethyl-dC**	2.85E+04	6.29E+04	3.13E+04	1.03E+05	1.41E+04	2.39E+04
**[D_5]N^4-ethyl-dC**	3.00E+03	6.02E+03	2.72E+05	1.81E+04	9.43E+04	1.76E+05
**N^6-ethyl-dA**	5.22E+04	8.00E+04	4.36E+04	6.20E+04	3.02E+04	1.04E+05
**[D_5]N^6-ethyl-dA**	5.48E+05	6.29E+05	4.61E+06	5.56E+05	1.82E+06	1.01E+07
**N^2-ethyl-dG**	7.80E+05	1.99E+06	1.67E+06	2.86E+06	4.39E+05	3.30E+06
**[$^{15}N_5$]N^2-ethyl-dG**	7.33E+03	1.41E+04	8.67E+05	1.40E+04	3.69E+05	1.11E+06

Table S4. Average (fmol/µmol dG), standard deviation (STD) and p-value of ethyl-adducts in alcohol-exposed and non-exposed oral cells (CTRL).

	**Alcohol (fmol/µmol dG)**	**CTRL (fmol/µmol dG)**	**STD Alcohol**	**STD CTRL**	**p-value**
**N^4-ethyl-dC**	1.18E+04	8.01E+01	4.17E+03	2.16E+01	0.0166
**N^6-ethyl-dA**	1.55E+02	7.06E+00	4.57E+01	1.80E+00	0.0103
**N^2-ethyl-dG**	2.08E+05	1.30E+03	6.92E+04	7.59E+02	0.0134

References

1. Dator, R.; Carrà, A.; Maertens, L.; Guidolin, V.; Villalta, P.W.; Balbo, S. A High Resolution/Accurate Mass (HRAM) Data-Dependent MS. *J Am Soc Mass Spectrom* **2017**, *28*, 608-618, doi:10.1007/s13361-016-1521-y.
2. Brooks, P.J.; Enoch, M.A.; Goldman, D.; Li, T.K.; Yokoyama, A. The alcohol flushing response: an unrecognized risk factor for esophageal cancer from alcohol consumption. *PLoS Med* **2009**, *6*, e50, doi:10.1371/journal.pmed.1000050.
3. Nakano, Y.; Ochi, H.; Onohara, Y.; Sairaku, A.; Tokuyama, T.; Matsumura, H.; Tomomori, S.; Amioka, M.; Hironomobe, N.; Motoda, C., et al. Genetic variations of aldehyde dehydrogenase 2 and alcohol dehydrogenase 1B are associated with the etiology of atrial fibrillation in Japanese. *J Biomed Sci* **2016**, *23*, 89, doi:10.1186/s12929-016-0304-x.
